# Genetic inhibition of serum glucocorticoid kinase 1 prevents obesity-related atrial fibrillation

**DOI:** 10.1172/jci.insight.160885

**Published:** 2022-10-10

**Authors:** Aneesh Bapat, Guoping Li, Ling Xiao, Ashish Yeri, Maarten Hulsmans, Jana Grune, Masahiro Yamazoe, Maximilian J. Schloss, Yoshiko Iwamoto, Justin Tedeschi, Xinyu Yang, Matthias Nahrendorf, Anthony Rosenzweig, Patrick T. Ellinor, Saumya Das, David Milan

**Affiliations:** 1 Cardiovascular Research Center, Massachusetts General Hospital, Harvard Medical School, Boston, Massachusetts, USA.; 2 Demoulas Family Foundation Center for Cardiac Arrhythmias, Massachusetts General Hospital, Boston, Massachusetts, USA.; 3 Center for Systems Biology, Massachusetts General Hospital Research Institute and Harvard Medical School, Boston, Massachusetts, USA.; 4 Department of Radiology, Massachusetts General Hospital, Boston, Massachusetts, USA.; 5 German Centre for Cardiovascular Research, Berlin, Germany.; 6 Fangshan Hospital of Beijing, University of Traditional Chinese Medicine, Beijing, China.; 7 Department of Internal Medicine I, University Hospital Wuerzburg, Wuerzburg, Germany.; 8 Leducq Foundation, Boston, Massachusetts, USA.

**Keywords:** Cardiology, Metabolism, Arrhythmias

## Abstract

Obesity is an important risk factor for atrial fibrillation (AF), but a better mechanistic understanding of obesity-related atrial fibrillation is required. Serum glucocorticoid kinase 1 (SGK1) is a kinase positioned within multiple obesity-related pathways, and prior work has shown a pathologic role of SGK1 signaling in ventricular arrhythmias. We validated a mouse model of obesity-related AF using wild-type mice fed a high-fat diet. RNA sequencing of atrial tissue demonstrated substantial differences in gene expression, with enrichment of multiple SGK1-related pathways, and we showed upregulated of SGK1 transcription, activation, and signaling in obese atria. Mice expressing a cardiac specific dominant-negative SGK1 were protected from obesity-related AF, through effects on atrial electrophysiology, action potential characteristics, structural remodeling, inflammation, and sodium current. Overall, this study demonstrates the promise of targeting SGK1 in a mouse model of obesity-related AF.

## Graphical abstract

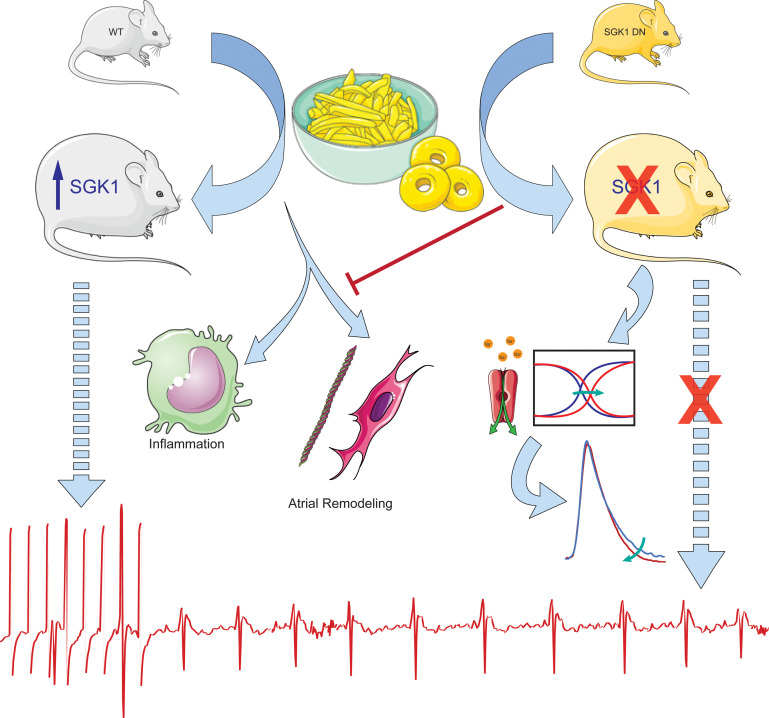

## Introduction

The rate of obesity in the United States has risen steadily since 1960 and reached a prevalence of 42% in 2017 to 2018 (1). With a pediatric obesity prevalence of 19% in 2017 to 2018 (2), we can reasonably expect obesity to persist as a significant public health issue. Atrial fibrillation (AF) is a common cardiac arrhythmia that affects about 5 million Americans and is associated with significant morbidity and mortality (3). Although many risk factors have been identified for AF, obesity has come to the forefront as a prominent but potentially modifiable one. Epidemiological studies demonstrate that nearly 1 in 5 cases of AF are associated with obesity (4). Importantly, data from the Women’s Health Study showed that obese individuals who lost weight to BMI less than 30 kg/m^2^ over a 5-year follow up period were found to have a reduced AF risk when compared to their persistently obese counterparts (5). Thus, not only is obesity a strong AF risk factor, but it may be one that is reversible.

The mechanistic relationship between obesity and AF is not completely delineated, and thus no specific therapies exist. However, a plethora of inciting factors have been proposed and include alterations in hemodynamics, neurohormonal axis, inflammation, metabolism, and adipokines (6). Animal models of prolonged high-fat diet feeding suggest a role for electroanatomic remodeling (7–10), inflammation (11), and decreased connexin expression (12). In addition, obesity is associated with activation of the Nod-like receptor family pyrin domain containing 3 (NLRP3) inflammasome (13), which can be manipulated in cardiomyocytes to produce an atrial substrate susceptible to AF (14).

Serum glucocorticoid kinase 1 (SGK1) is a PI3K-dependent kinase, with structure similar to AKT (15). SGK1 expression and activation lies downstream of both insulin signaling pathways and mineralocorticoid receptor activation (16), both of which are associated with human AF (17–24). Additionally, systemic upregulation of both upstream pathways have been noted in multiple models of diet-induced obesity (25–28), positioning SGK1 as a potential key mediator of AF pathogenesis in the context of obesity. Initial studies of SGK1 demonstrated that the signaling pathway can be activated in the heart by transverse aortic constriction, where acute activation promotes cardiomyocyte survival (29). However, SGK1 was subsequently shown to be activated in human heart failure, pointing to its role in maladaptive cardiac remodeling. Cardiac specific expression of constitutively active SGK1 in mice was associated with worsened transverse aortic constriction–induced (TAC-induced) cardiac dysfunction and lethal ventricular arrhythmias through electrical remodeling caused by the late sodium current (*I_Na_*), while genetic inhibition was protective (30). SGK1 signaling has also been implicated in murine cardiac inflammation (31) and NLRP3 inflammasome activation (32) resulting from angiotensin II infusion–induced hypertension. SGK1 signaling therefore presents a unique and attractive therapeutic target given its presumed involvement in multiple obesity-related pathways and its ability to directly modulate cardiac electrophysiology through effects on the *I_Na_*.

Based on its position downstream of metabolic pathways and its known role in electrophysiology, our a priori hypothesis was that obesity may induce pathologic SGK1 signaling. We sought to support our hypothesis with a comprehensive assessment of the effects of obesity on atrial gene expression and found multiple SGK1-related pathways to be upregulated, including insulin and mineralocorticoid signaling. We then postulated that genetic inhibition of SGK1 would be protective in obesity-related AF through attenuation of obesity-related atrial electroanatomic remodeling and inflammation.

## Results

### Diet-induced obesity results in increased AF susceptibility and marked transcriptional changes.

**Figure 1:**
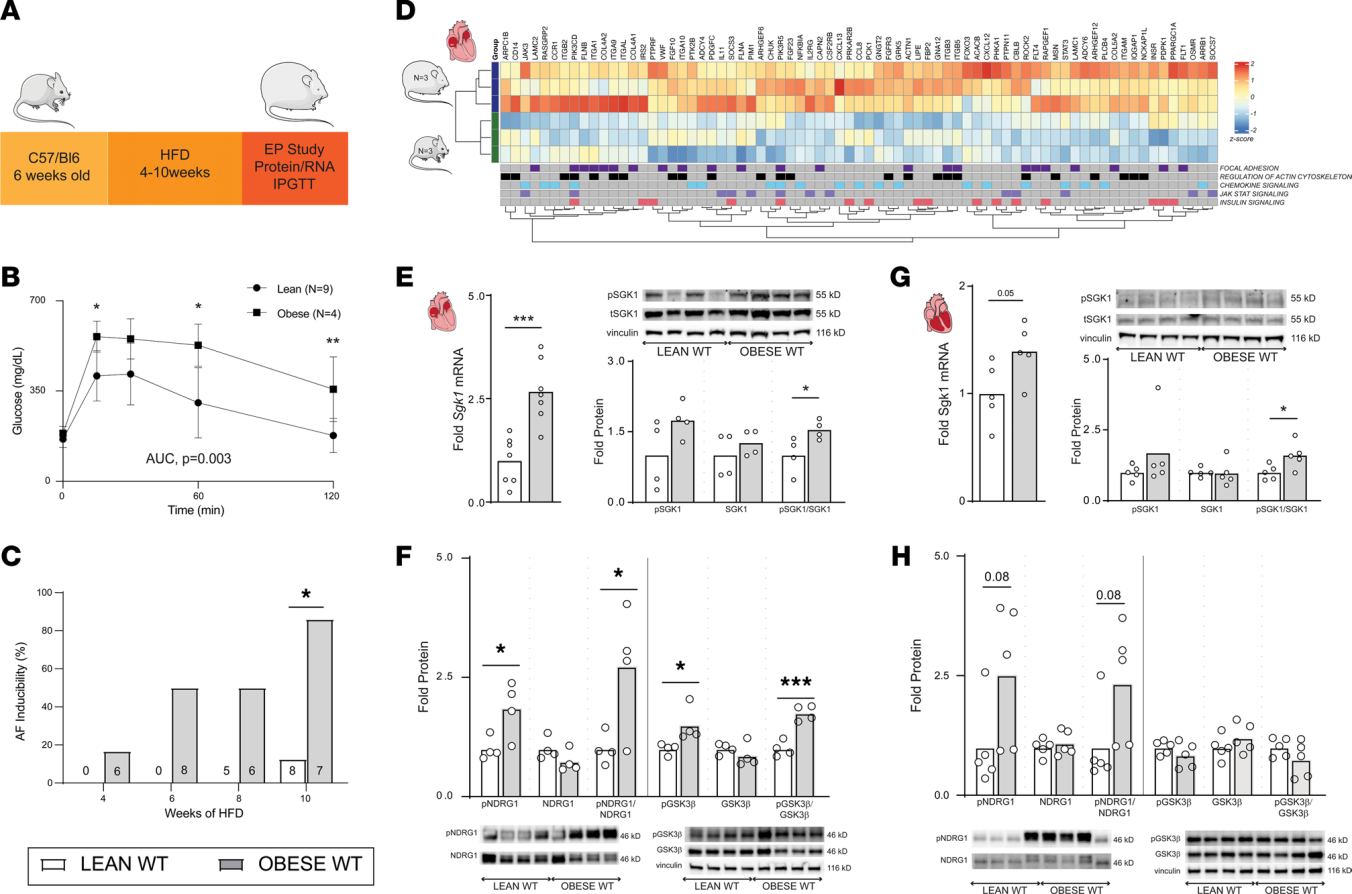
Diet-induced obesity results in increased AF inducibility and is associated with upregulation of SGK1 signaling. (**A**) Schematic of HFD feeding in WT mice to generate obese WT mice. IPGTT, intraperitoneal glucose tolerance testing. (**B**) Glucose levels after glucose tolerance test in lean and obese mice. (**C**) AF inducibility in lean and obese mice after specified period of feeding control or HFD chow. (**D**) Heatmap derived from RNA-sequencing data demonstrating GSEA core enriched genes in SGK1-related pathways (FWER *P* < 0.05) that are differentially expressed (nominal *P* < 0.05). (**E**) Atrial *Sgk1* mRNA expression in obese versus lean mice (left) and expression of phosphorylated (pSGK1), total SGK1 protein, and ratio as quantified by Western blotting (right). (**F**) Ventricular *Sgk1* mRNA expression in obese versus lean mice (left) and expression of pSGK1, total SGK1 protein, and ratio as quantified by Western blotting (right). (**G**) Atrial expression of SGK1 phosphorylation targets NDRG1 and GSK3β in obese versus lean mice. The NDRG1 and GSK3β blots were from the same gel and so share a vinculin loading control. (**H**) Ventricular expression of SGK1 phosphorylation targets NDRG1 and GSK3β in obese versus lean mice. The NDRG1 and GSK3β blots were from the same gel and so share a vinculin loading control. For all parts of Figure 1, unpaired Student’s *t* test. *P < 0.05, **P < 0.01, and ***P < 0.001. Number of mice in each group provided in the legend or bar graph or represented by the number of dots in individual figure.

We used diet-induced obesity to create a mouse model of obesity-related AF. C57BL/6J wild-type (WT) mice were fed a high-fat diet (HFD) starting at 6 weeks of age (Figure 1A). We performed IPGTT in lean (average weight 30 ± 2.8 g) and obese (average weight 42 ± 4.1 g) WT mice after 10 weeks of the appropriate diet. This assessment supported a significant difference in glucose tolerance as quantified by AUC, as well as significant differences in blood glucose 15, 60, and 120 minutes after glucose challenge, overall suggesting insulin resistance (Figure 1B). We then performed terminal electrophysiology studies to assay arrhythmogenesis after 4 to 10 weeks of HFD. Although only 1 out of the 6 mice studied was inducible for AF greater than 1 second after 4 weeks of HFD (average weight 31 ± 2.2 g), 6 out of 7 were inducible for AF after 10 weeks of HFD. In comparison, among age-matched mice fed a control diet for 10 weeks, only 1 out of 8 was inducible for AF during electrophysiology studies (Figure 1C). Given this stark phenotype at 10 weeks of HFD, we proceeded to use 10 weeks of HFD as a model for obesity-related AF.

In addition to electrophysiology studies, a separate group of WT mice were euthanized after 10 weeks of HFD to assess the transcriptional effects of obesity in atrial tissue. RNA sequencing was performed in atrial tissue of lean and obese WT C57BL/6 mice, followed by gene set enrichment analysis (GSEA) with Kyoto Encyclopedia of Genes and Genomes (KEGG) pathways to identify affected biological processes. There was marked divergence in the transcriptional profile of lean and obese atria, with 444 differentially expressed genes (279 upregulated, 165 downregulated) between the 2 groups (*P*_adj_ < 0.05). (Supplemental Table 1; supplemental material available online with this article; https://doi.org/10.1172/jci.insight.160885DS1). All positively and negatively enriched KEGG pathways at a family-wise error rate (FWER) *P* < 0.05 are listed in Supplemental Tables 2 and 3.

Notable among these were KEGG pathways that either directly involve SGK1 signaling or lie upstream or downstream of SGK1. Using a broader catchment with a false discovery rate (FDR) < 0.25, we identified 10 SGK1-related KEGG pathways, which are presented in Table 1. Among these, SGK1 is directly involved in MTOR SIGNALING and ALDOSTERONE REGULATED SODIUM ABSORPTION. Beyond this, SGK1 lies downstream of a number of obesity-regulated pathways, including CHEMOKINE SIGNALING, INSULIN SIGNALING, and MAPK SIGNALING. The 5 SGK1-related pathways with FWER *P* < 0.05 are shown in Figure 1D, with differentially expressed (nominal *P* < 0.05), core, enriched genes depicted in the heatmap. Besides significant upregulation in multiple pathways associated with SGK1 signaling, the RNA-sequencing data also suggested an increase in expression of *Sgk1* itself, with a log_2_ fold change of 0.285 and a nominal *P* value of 0.046. These data supported our hypothesis that obesity-related pathophysiology may overlap with or involve SGK1 signaling. In addition to these findings, the potentially unique role of SGK1 signaling in *ventricular* proarrhythmia mediated by electrical remodeling presented it as an opportunistic target for treatment of obesity-related *atrial* arrhythmias.

### Diet-induced obesity activates SGK1 signaling.

The above-noted increase in *Sgk1* gene expression was verified with focused quantitative PCR, as HFD resulted in a significant increase in atrial SGK1 gene expression, with a similar trend in the ventricles (Figure 1, E and F). In addition, in both atrial and ventricular tissue, HFD feeding led to a significant increase in the ratio of phosphorylated to total SGK1 as determined by Western blotting (Figure 1, E and F). To further ascertain SGK1 signaling, we quantified phosphorylation of known SGK1 targets. In fact, there was a marked increase in phosphorylation of SGK1-specific target N-myc downstream regulated 1 (NDRG1) in both obese atrial and ventricular tissue. Similarly, in obese atrial tissue, there was an increase in phosphorylated glycogen synthase kinase 3 beta (GSK3β); there was no difference in phosphorylated GSK3β in ventricular tissue (Figure 1, G and H). Overall, these data demonstrate that diet-induced obesity resulted both in increased AF inducibility, as well as an increase in SGK1 transcription, SGK1 activation through phosphorylation, and activity assayed by phosphorylation of known SGK1 targets. Interestingly, these findings appear more pronounced in atrial, rather than ventricular, tissue. We then investigated the possibility of a dose response relating HFD/obesity with atrial SGK1 signaling, by comparing mice fed HFD for 6 weeks (which have ~50% AF inducibility) with mice fed HFD for 10 weeks. As would be expected, we found an increase in SGK1 and downstream target phosphorylation in mice fed HFD for 10 weeks as compared with their counterparts fed for only 6 weeks (Supplemental Figure 1). Given the known role of SGK1 in electrical/structural remodeling, we sought to investigate the role of SGK1 in obesity-induced atrial pathology.

### Genetic SGK1 inhibition alters atrial electrophysiology and prevents obesity-related AF.

**Figure 2:**
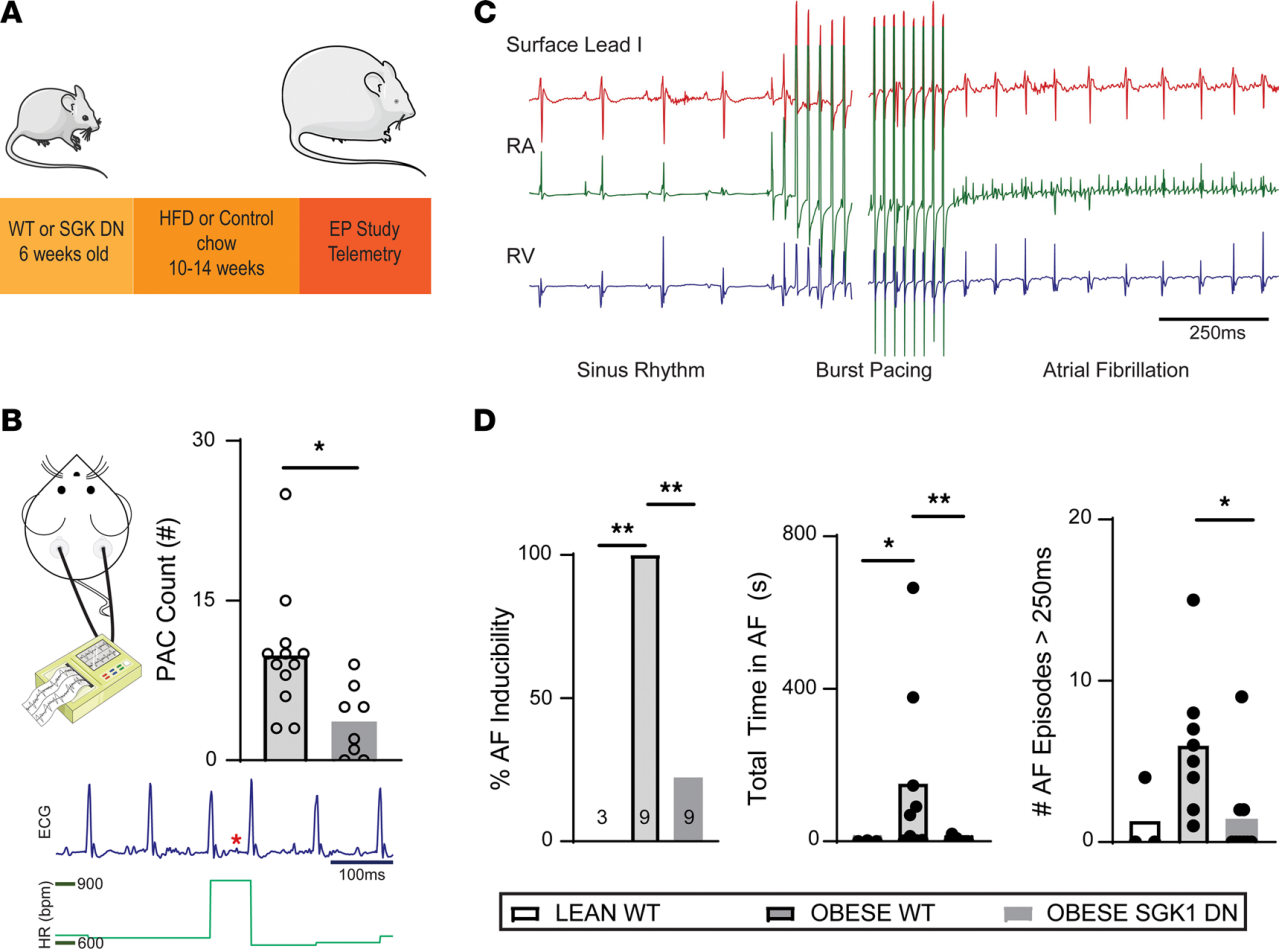
SGK1 DN mice are protected from obesity-induced AF. (**A**) Schematic of mouse model used in these studies. (**B**) Frequency of premature atrial complexes (PACs) recorded during continuous telemetry recordings over an 8-hour period. Below the quantified plot is a representative telemetry tracing; the red star identifies an ectopic atrial P wave; and the dark blue scale bar is 100 ms. The green plot graphs heart rate (bpm) during the tracing of interest. Unpaired Student’s *t* test. *P < 0.05. (**C**) Example surface EKG and intracardiac tracing showing pacing-induced AF. (**D**) AF inducibility, by percentage inducible, of lean and obese mice (left); total summed duration of all AF episodes during each electrophysiology study (middle); and number of episodes of induced AF > 250 ms in duration by mouse (right). Fisher’s exact test for AF inducibility and Kruskal-Wallis test with post hoc Dunn’s test for duration/burden. *P < 0.05, **P < 0.01. Number of mice in each group is provided in bar graph or represented by the number of dots in individual figure.

To test whether SGK1 plays a role in obesity-induced AF, we employed a previously described transgenic mouse model that overexpresses a dominant-negative form of SGK1 in cardiomyocytes (SGK1 dominant negative, DN) (30). After 10–14 weeks of HFD, 79% of SGK1 DN (23 out of 29) and 73% of WT littermates (22 out of 30) achieved a minimum 34 g weight (Figure 2A). Over the course of feeding, there were no differences in weight gain or the final weight between SGK1 DN and WT littermates; both were significantly heavier than the WT mice fed a normal chow at the end of the feeding period (*P* < 5 × 10^–5^ for both). Tail cuff blood pressure measurements showed no difference in systolic, diastolic, or mean arterial pressure between the lean WT, obese WT, or obese SGK1 DN mice. Two-dimensional transthoracic echocardiography was performed and did not reveal any differences in left ventricular (LV) dimensions, LV systolic or diastolic function, or left atrial size between the 3 groups of mice. Both obese WT and obese SGK1 DN mice demonstrated impaired glucose tolerance as compared with lean WT mice, but there were no significant differences between the 2 obese groups (Supplemental Figure 2). Importantly, assessment of downstream phosphorylation targets in SGK1 DN atria showed a significant decrease in the phosphorylation of NDRG1 and a trend toward the same with respect to GSK3β (Supplemental Figure 3).

Ambulatory telemetry was performed in obese SGK1 DN and WT mice. Eight-hour periods (from midnight to 8 am) were analyzed to assess for baseline characteristics as well as spontaneous arrhythmias. There were no significant differences in baseline ambulatory heart rate or the root mean square of the successive differences, a marker of heart rate variability, between the 2 groups (Supplemental Figure 2). No sustained spontaneous arrhythmias (atrial or ventricular) were recorded during the analyzed periods. However, there was a difference in the number of premature atrial complexes (PACs), which was significantly higher in the obese WT group as compared with the obese SGK1 DN group (Figure 2B). The latter is suggestive of an increased atrial excitability that may serve to trigger AF.

Terminal electrophysiology studies were performed to determine electrophysiology parameters with focus on AF inducibility. Parameters related to sinus node function, atrioventricular (AV) node function, and refractory periods in atria and ventricles are presented in Table 2. There were no significant differences in baseline parameters among the 3 groups. Programmed electrical stimulation with up to 2 extrastimuli and rapid burst pacing were performed in both the atria and ventricles. AF inducibility was significantly higher in obese (9 out of 9) versus lean mice (0 out of 3), but this difference was abrogated in obese SGK1 DN mice (2 out of 9). There were similar differences in number of AF episodes (>250 ms) and total AF burden between lean and obese WT mice, both of which were prevented by SGK1 genetic inhibition (Figure 2, C and D).

**Figure 3:**
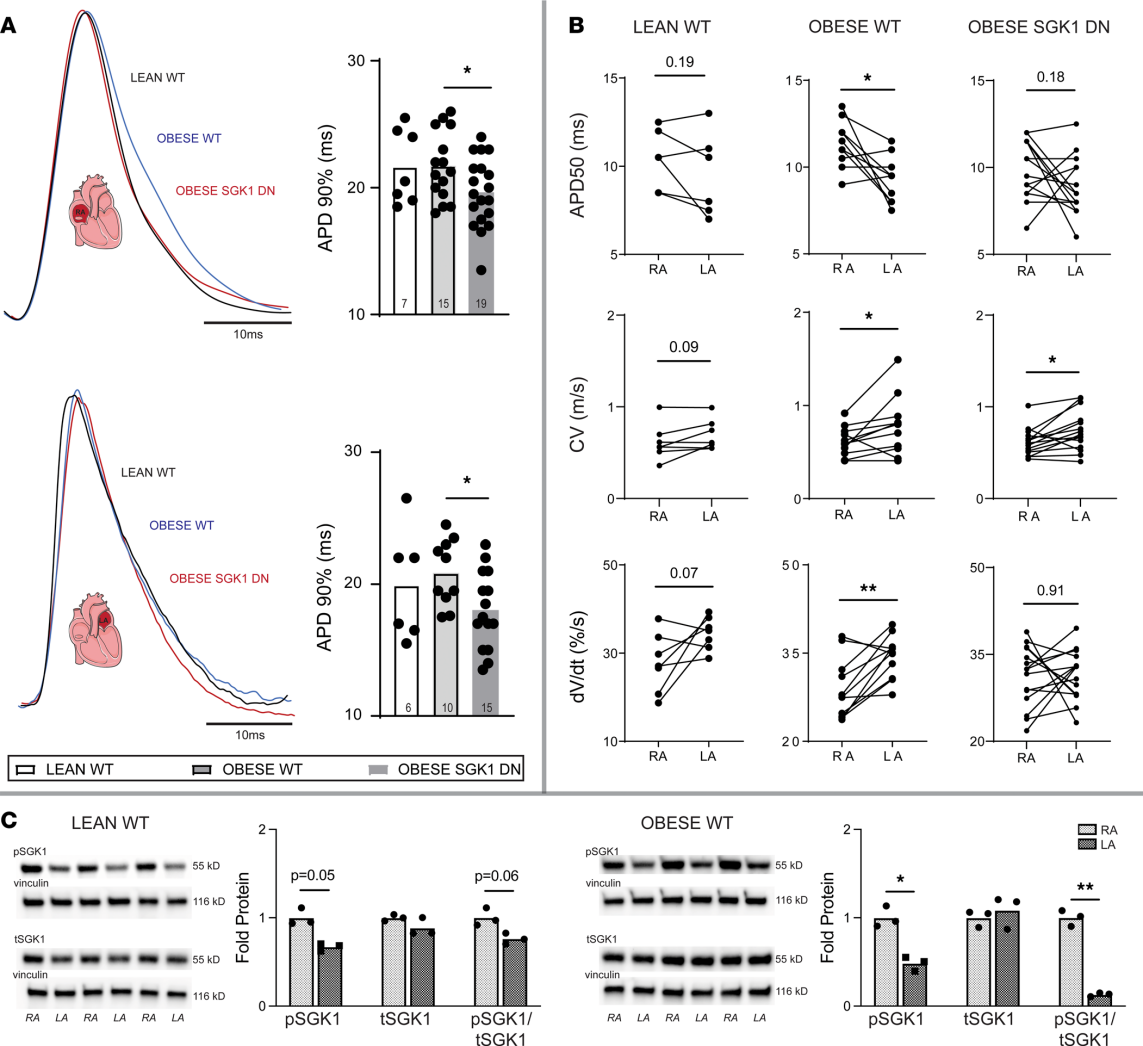
Atrial electrophysiologic effects of SGK1 genetic inhibition on obese mice. (**A**) Right (upper) and left (lower) atrial APD90 in lean and obese WT and obese SGK1 DN mice. Tracings show representative optical AP tracings. One-way ANOVA, Dunnett’s test. *P < 0.05. (**B**) Interatrial differences in APD50 (top), CV (middle), and upstroke velocity (*dV/dt*, bottom) in lean WT (left), obese WT (middle), and SGK1 DN (right) mouse atrial chambers. Paired Student’s *t* test. *P < 0.05, **P < 0.01. (**C**) Representative blots and quantification of right atrial (RA) and left atrial (LA) expression of phosphorylated SGK1, total SGK1, and the ratio of phosphorylated to total SGK1 (pSGK1/tSGK1) in lean (left) and obese (right) atria. Paired Student’s *t* test. *P < 0.05, **P < 0.05. Number of mice in each group is represented by the number of dots in individual figure.

Optical mapping of right atrial and left atrial appendages was performed on these 3 groups of mice using a voltage-sensitive dye (Table 3). There were no significant differences in lean versus obese WT mouse action potential duration (APD), conduction velocity (CV), or action potential (AP) upstroke velocity in either atrial chamber. Overall, the APD was shorter in the obese SGK1 DN as compared with the obese WT mice in both atrial chambers and met statistical significance APD90 (Figure 3A). There were no significant differences in CV between the 2 obese mouse genotypes. Interestingly, within the WT obese mice, there were significant inter-atrial differences in APD50, CV, and *dV/dt*; lean WT mice had no significant analogous inter-atrial differences, and SGK1 DN mice only had inter-atrial differences in CV, with no significant inter-atrial differences in APDs or *dV/dt* (Figure 3B). To interrogate this further, we assessed expression and phosphorylation status of SGK1 protein in right and left atria of lean and obese mice (Figure 3C). Pairwise comparisons revealed generally higher SGK1 phosphorylation in right versus left atria, but this difference was exaggerated and met statistical significance in the obese mice.

**Figure 4:**
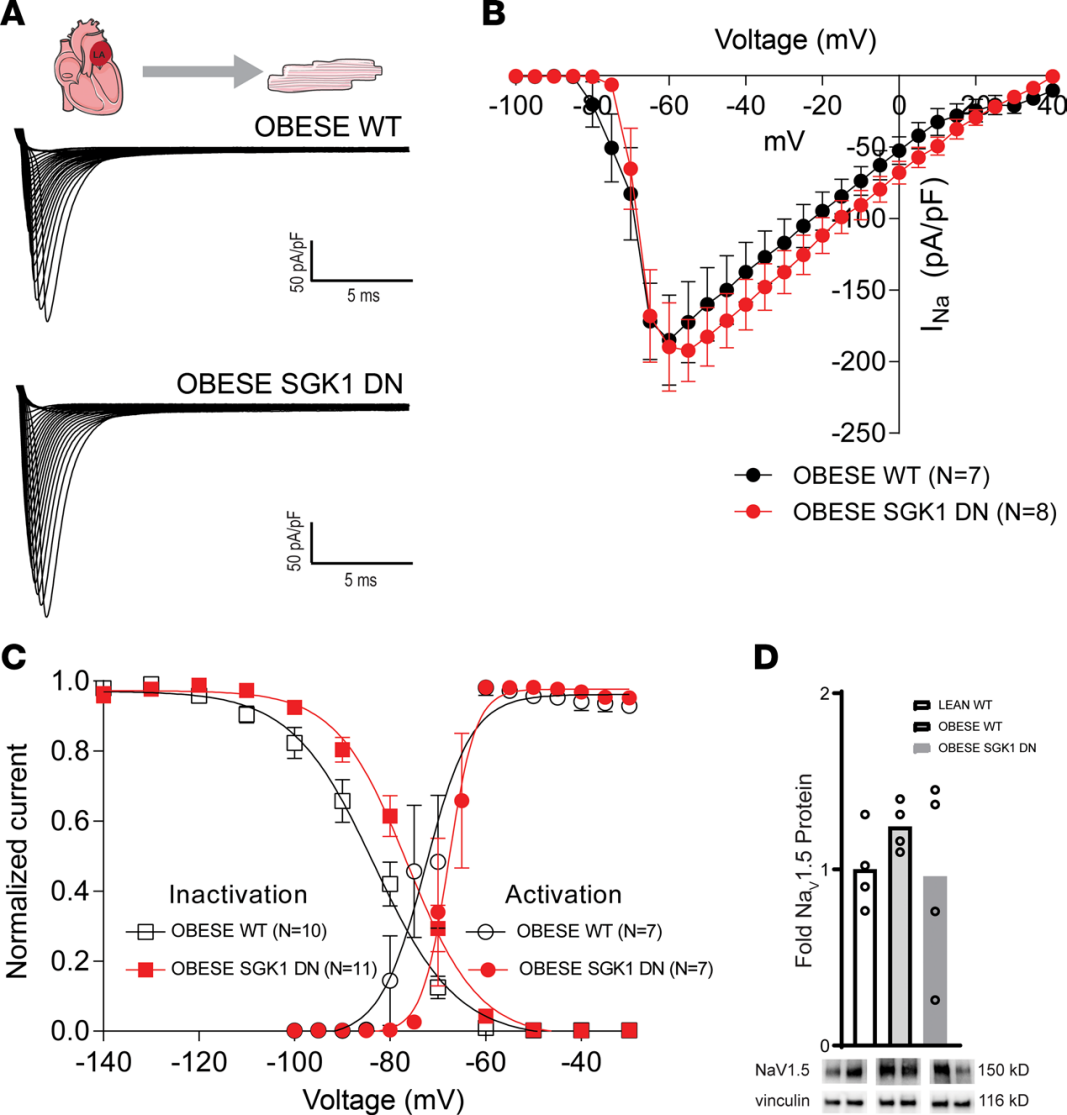
Effect of SGK1 genetic inhibition on *I_Na_* in obese LA cardiomyocytes. (**A**) Example *I_Na_* recordings from isolated LA cardiomyocytes of obese WT and SGK1 DN mice. (**B**) Current-voltage relations in WT (black) and SGK1 DN (red) isolated left atria. (**C**) *I_Na_* activation-inactivation voltage dependences for WT (black) and SGK1 DN (red) LA cardiomyocytes, demonstrating a rightward depolarizing shift in the SGK1 DN myocytes. (**D**) Atrial expression of Na_V_1.5 subunit with representative blots below. In **B** and **C**, data depicted as mean ± SEM. Number of mice in each group provided in legend or represented by the number of dots in individual figure.

We performed focused patch-clamping on atrial myocytes to further investigate the shorter APDs in setting of SGK1 inhibition. Adipokines such as leptin have been shown to increase the late *I_Na_* in LA myocytes (33). Prior work has demonstrated the effect of SGK1 activation on *I_Na_* via alteration of channel biophysical properties, as well as trafficking and localization of Na_V_1.5, the pore-forming subunit of the channel (30). We suspected that the protective electrophysiologic consequences of SGK1 genetic inhibition may result from its effects on the *I_Na_*. Whole-cell patch-clamp was performed on obese WT and obese SGK1 DN LA myocytes (Figure 4A). There were no significant differences in peak *I_Na_* between the 2 groups (Figure 4B). However, SGK1 DN myocytes demonstrated a rightward depolarizing shift in activation-inactivation kinetics of *I_Na_* in SGK1 DN myocytes (inactivation V_1/2_ SEM: WT, –84 ± 2 mV, *n* = 10, SGK1 DN, –77 ± 2 mV, *n* = 11, *P* < 0.05 WT versus DN). However, there were no significant differences in slope factors (mean ± SEM: WT 0.3 ± 0.1, SGK1 DN 0.4 ± 0.1, *P* = 0.62) or activation V_1/2_ (mean ± SEM: WT –75 ± 2.3 mV, SGK1 DN –70.75 ± 1.8 mV, *P* = 0.145) (Figure 4C). This change in the activation-inactivation curves is diametrically opposed to existing literature on SGK1 constitutively active (CA) cardiomyocytes, which have a *leftward* shift in activation-inactivation kinetics as compared with WT cells (30). The rightward shift in activation-inactivation kinetics observed in our study would be expected to decrease the persistent (late) Na current (34). As the effects of SGK1 signaling on *I_Na_* are mediated by posttranslational modification and trafficking, there were unsurprisingly no differences in expression of the Na_V_1.5 protein subunit as assessed by Western blotting in lean WT, obese WT, or obese SGK1 DN mouse atria (Figure 4D). These data are consistent with previously published data regarding the effect of SGK1 signaling on *I_Na_* in ventricular myocytes (30) and may account for the differences between obese WT and SGK1 DN mouse atria determined through optical mapping. In addition, these data suggest that inter-atrial differences in activation of SGK1 (via phosphorylation) may contribute to the correlating differences in electrophysiology, particularly APD, between the 2 atrial chambers.

### Genetic inhibition of SGK1 prevents cardiac remodeling.

**Figure 5:**
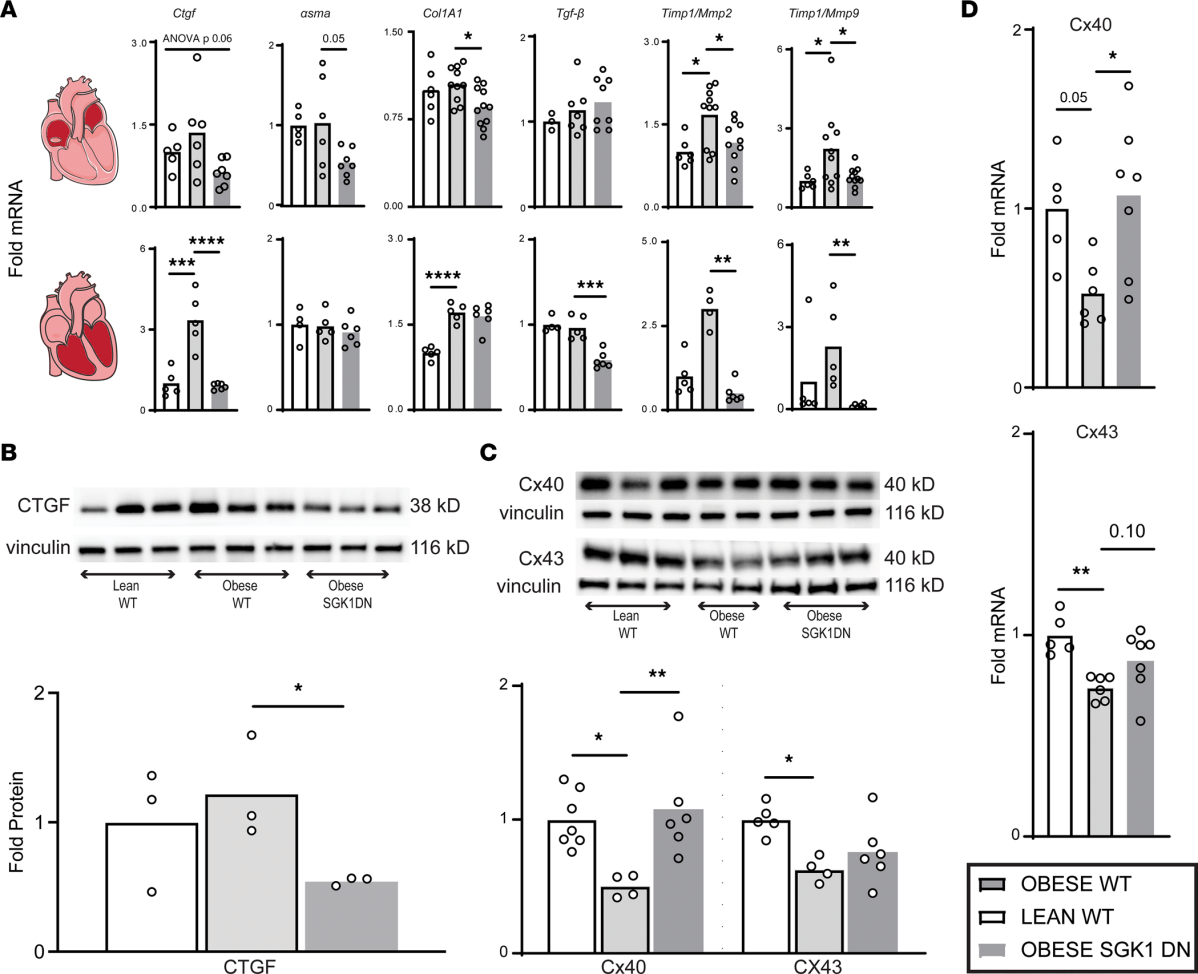
SGK1 genetic inhibition prevents obesity-induced atrial fibrotic signaling. (**A**) Expression of fibrosis-related genes in lean and obese atria and ventricles. One-way ANOVA, Dunnett’s test or Kruskal-Wallis, Dunn’s test (only LV *Timp1*/*Mmp2*, LV *Timp1*/*Mmp9*). *Timp1*, tissue inhibitor of matrix metalloproteinase 1. *P < 0.05, **P < 0.01, ***P < 0.001, ****P < 0.0001. (**B**) Atrial CTGF protein expression with representative blots. One-way ANOVA, Dunnett’s test. *P < 0.05. (**C**) Connexin protein expression measured by Western blot, with representative blots. One-way ANOVA, Dunnett’s test. *P < 0.05, **P < 0.01. (**D**) Expression of connexin 40 (above) and 43 (below) mRNA transcripts. One-way ANOVA, Dunnett’s test. *P < 0.05, **P < 0.01. Number of mice in each group is represented by the number of dots in individual figure.

SGK1 DN mouse ventricles are resistant to TAC-induced fibrosis (30), so we hypothesized that SGK1 inhibition may protect from obesity-induced structural remodeling in the atria. We initially performed Masson’s trichrome staining to assess for obesity-related atrial fibrosis. Although obesity-induced atrial fibrosis is described in literature (35, 36), this is an insensitive technique susceptible to sampling bias, and we did not, in fact, find a difference in fibrosis (Supplemental Figure 4). However, given the relatively short duration of HFD, we suspected that genetic expression may better reflect the early profibrotic consequences of obesity. Given the association between both atrial and ventricular fibrosis and atrial fibrillation, reverse transcription quantitative PCR (RT-qPCR) of fibrosis-related genes was performed in both tissues. There seemed to be a generalized obesity-induced increase in profibrotic gene expression in the ventricles but no significant obesity-induced differences in atrial tissue. However, SGK1 inhibition did result in generally decreased expression of these profibrotic genes in both atria and ventricles (Figure 5A). Activity — and ratio — of MMPs and TIMPs are known to affect the maintenance and turnover of the ECM (37–39). HFD feeding was associated with a significantly increased atrial/ventricular expression of *Timp1* and plasminogen activator inhibitor 1 (*Pai1*) in both atria (*Timp1* *P* = 0.03; *Pai1* *P* = 0.01) and the left ventricle (*Timp1* *P* = 0.06; *Pai1* *P* = 0.0005), both of which are associated with increased ECM turnover. These obesity-induced profibrotic changes were significantly reduced in ventricular (*Timp1* *P* = 0.01; *Pai1* *P* = 0.0005) — but not atrial — tissue of SGK1 DN mice. However, the ratios of *Timp1/Mmp2* and *Timp1/Mmp9* were increased in obese WT atria/ventricles as compared with lean WT mouse atria/ventricles, but these ratios were significantly lower in the obese SGK1 DN mice as compared with obese WT mice (Figure 5A). In addition to differences in gene transcription, Western blotting revealed a significant decrease in atrial protein expression of connective tissue growth factor (CTGF) in SGK1 DN as compared with WT obese mouse atria (Figure 5B). Overall, these data demonstrate decreased profibrotic signaling in SGK1 DN cardiac tissue even in the relatively short time studied, consistent with the previously described finding of reduced ventricular fibrosis in the TAC model.

Multiple animal models have demonstrated the obesity-related downregulation of cardiac connexin proteins (11, 12). We supported these results in our study by determining the level of gene and protein expression. RT-qPCR showed a decrease in atrial expression of both connexin 40 and 43 in obese as compared with lean mice, but the decrease was mitigated or reversed by SGK1 inhibition. These findings extended to protein translation, as immunoblotting revealed obesity-related decrease in atrial protein expression of connexin 40 and 43, which were ameliorated with SGK1 inhibition (Figure 5, C and D). The data presented here suggest a protective effect of SGK1 inhibition in terms of profibrotic signaling and cell-cell connectivity.

### Genetic inhibition of SGK1 prevents obesity-induced inflammation.

**Figure 6:**
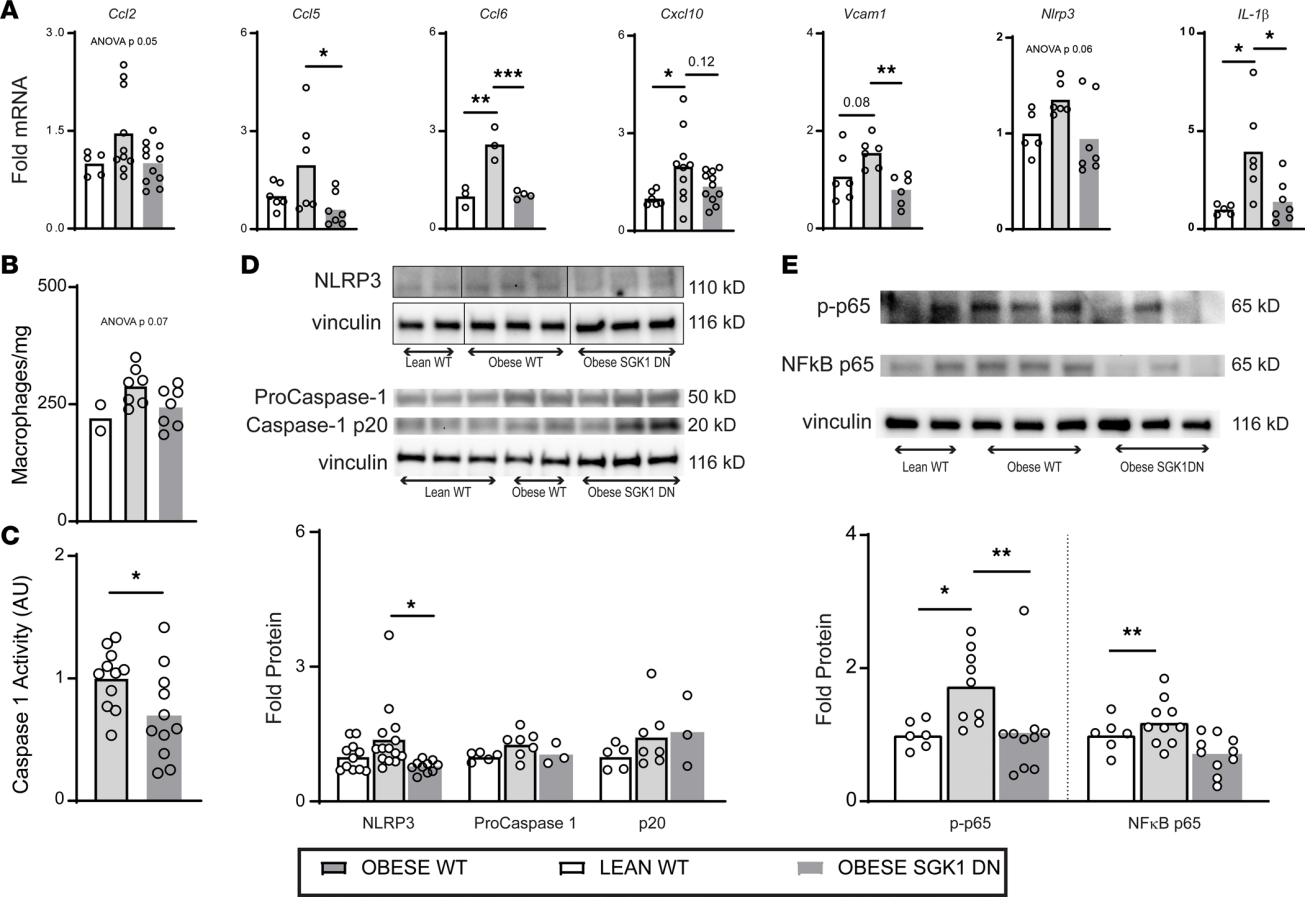
SGK1 genetic inhibition prevents obesity-induced atrial inflammatory signaling. (**A**) Atrial inflammatory gene transcript levels. One-way ANOVA, Dunnett’s test. *P < 0.05, **P < 0.01, ***P < 0.001. (**B**) Atrial macrophage content in lean and obese atria as measured by flow cytometry. One-way ANOVA. (**C**) Caspase-1 activity in obese atrial tissue as measured with a commercially available assay. Unpaired Student’s *t* test. *P < 0.05. (**D**) Western blots of atrial NLRP3 and caspase subunits with quantification below. One-way ANOVA, Dunnett’s test. *P < 0.05. (**E**) Western blots of atrial NF-κB subunit p65 and its phosphorylated isoform with quantification below. One-way ANOVA, Dunnett’s test. *P < 0.05, **P < 0.01. Number of mice in each group is represented by the number of dots in individual figure.

Inflammation is thought to be involved in obesity-induced pathology (including fibrosis) (40), and we suspected that SGK1 signaling may contribute to proinflammatory pathways in the heart. We therefore evaluated the effect of SGK1 inhibition on atrial inflammation caused by obesity with RT-qPCR of inflammatory genes in WT lean, WT obese, and SGK1 DN obese mice. The genes of interest were either cytokines known to be involved in obesity-related inflammation or those thought to lie downstream of either mineralocorticoid or SGK1 signaling (32, 41–44). Once again, cardiomyocyte-specific genetic inhibition of SGK1 minimized obesity-induced increases in genes related to inflammation (Figure 6A). Given that SGK1 inhibition is restricted to cardiomyocytes, it is not surprising that plasma levels of circulating cytokines such as IL-6 and C-reactive protein were not impacted (Supplemental Figure 5).

Of note, there was a marked obesity-induced atrial *Il-1β* expression, which was abolished with genetic inhibition of SGK1. SGK1 activation has been shown to affect expression of *Ccl2*, which is involved in the recruitment of inflammatory cells, such as macrophages (41). Our data demonstrated a trend toward an increase in obesity-induced *Ccl2* expression, which was prevented by SGK1 genetic inhibition (ANOVA *P* = 0.05). We interrogated this pathway further with flow cytometry and determined that SGK1 genetic inhibition abrogated a trend toward an increased atrial macrophage content caused by obesity (Figure 6B). Although one could presume sheer difference in quantity of inflammatory cells as the source of the increase in *Il-1β*, we pursued this avenue further by investigating downstream pathways.

We studied 2 interrelated pathways that we suspected may have been inhibited with SGK1 inhibition: the NLRP3 inflammasome and NF-κB signaling. The NLRP3 inflammasome has been implicated in various obesity-related pathologies, including AF, and has been shown to be inhibited by pharmacologic SGK1 inhibition (32). Additionally, SGK1 activity has been shown to provoke nuclear translocation and hence activation of NF-κB (45–47). We assayed inflammasome activity with a fluorometric measurement of caspase activity, which was found to be significantly lower in atrial tissue of SGK1 DN mice compared with WT (Figure 6C). Similar to *Nlrp3* gene expression, NLRP3 protein expression showed a trend toward an increase with HFD feeding, which was significantly reduced with genetic SGK1 inhibition. There were not, however, differences in caspase-1 protein expression (Figure 6D). Finally, electrophysiology studies were performed in age-matched obese NLRP3^–/–^ mice, which demonstrated an intermediate AF phenotype, with 4 out of 10 mice inducible for AF greater than 1 second in duration (Supplemental Table 4).

NLRP3 expression is closely linked to NF-κB activation, which is mechanistically downstream of SGK1 activation (48, 49). Western blotting of the p65 subunit of NF-κB revealed an obesity-related significant increase in the phosphorylated — and hence transactivated (50, 51) — form of the protein, which was reversed by SGK1 genetic inhibition. Although there was no significant difference in the total expression of NF-κB p65 between WT lean and obese mice, there was significantly less NF-κB p65 protein expression in obese SGK1 DN mouse atria as compared with WT counterparts (Figure 6E). Overall, these data suggest that the protective effect of SGK1 inhibition in *Il-1β* expression may be mediated, in part, by its role in modulating the NLRP3 and NF-κB pathways.

### Constitutive SGK1 activation may modulate AF susceptibility in HFD-induced obesity.

**Figure 7:**
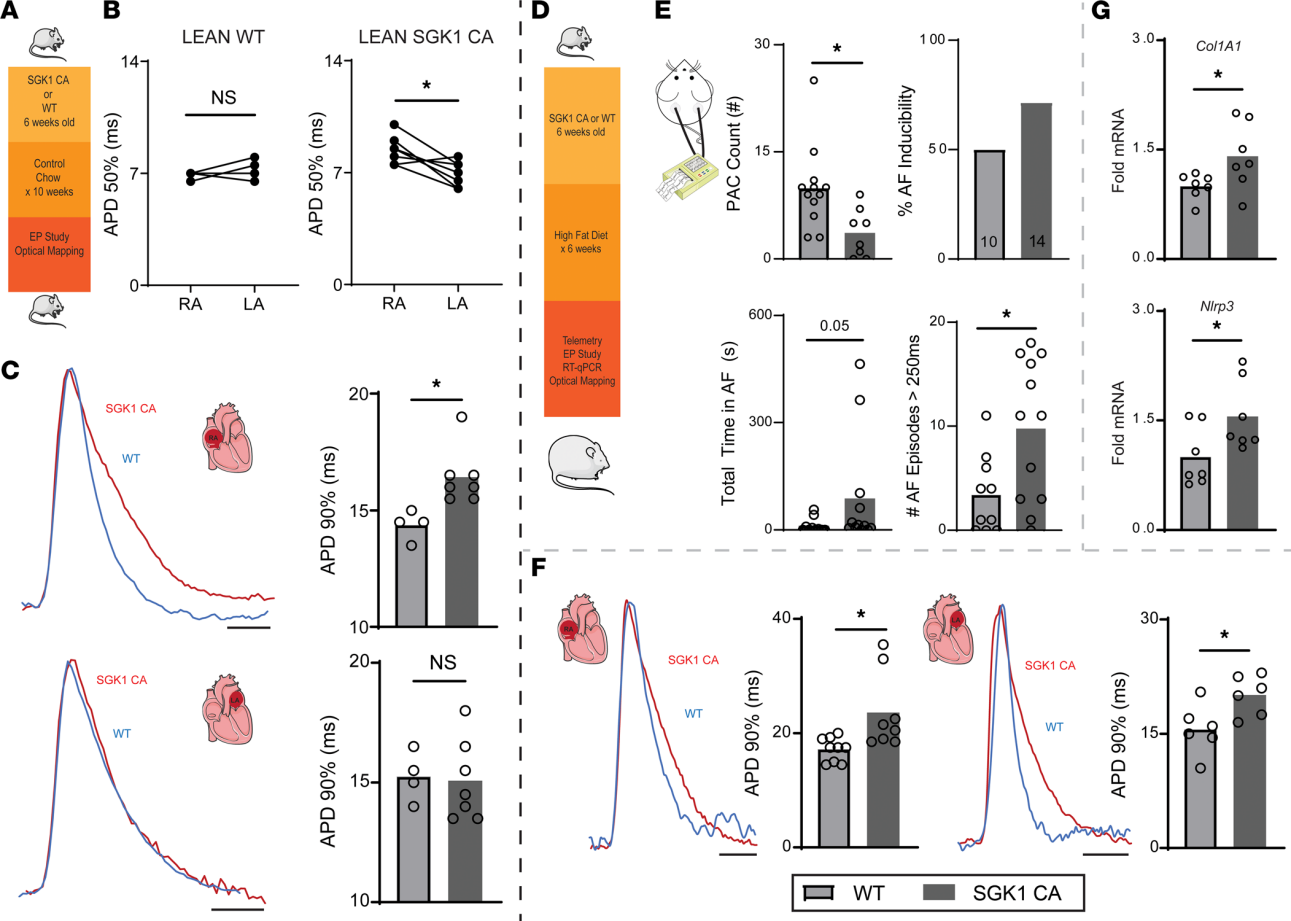
Constitutive SGK1 activation may increase susceptibility to obesity-induced AF. (**A**) Lean SGK1 CA and lean WT littermates were studied with electrophysiology studies and optical mapping. (**B**) Inter-atrial difference in APD50 in SGK1 CA atria. Paired Student’s *t* test. *P < 0.05. (**C**) Optical mapping–derived APD90 in right (top) and left (bottom) atria with representative AP tracings. Unpaired Student’s *t* test. *P < 0.05. (**D**) SGK1 CA and WT mice were fed an HFD for 6 weeks and then underwent electrophysiologic and biochemical studies. (**E**) Electrophysiologic assessment of WT and SGK1 CA mice fed HFD for 6 weeks with telemetry for PAC quantification (top left) and invasive electrophysiologic studies to determine AF inducibility (>1 second) (top right), total AF burden during electrophysiology study (bottom left), and total number of AF episodes > 250 ms (bottom right). *P* values obtained with unpaired Student’s *t* test for PAC count, Fisher’s exact test for AF inducibility, and Mann-Whitney test for AF burden and frequency. *P < 0.05. (**F**) Optical mapping–derived APD90 in right (left) and left (right) atria with representative AP tracings. Unpaired Student’s *t* test. *P < 0.05. (**G**) qRT-PCR–derived expression of *Col1A1* and *Nlrp3* atrial tissue. Unpaired Student’s *t* test. *P < 0.05. Number of mice in each group provided in bar graph or represented by the number of dots in individual figure.

To determine whether SGK1 overactivation on its own affects AF susceptibility, we performed electrophysiology studies and optical mapping on age-matched lean SGK1 CA and WT mice (Figure 7A). There were no differences in AF inducibility noted between the 2 groups. Interestingly, SGK1 CA mice demonstrated a significantly longer PR interval and had a trend (*P* = 0.14) toward a longer Wenckebach cycle length than their WT counterparts, suggesting an effect on AV node function. Otherwise, there were no significant differences in electrophysiology parameters between the 2 lean groups (Supplemental Table 5). Optical mapping of lean SGK1 CA mouse hearts reproduced the inter-atrial heterogeneity seen with obese WT mice, as the RA APD50 was significantly longer than LA APD50; there was no correlating inter-atrial difference in lean WT mouse atria (Figure 7B). In addition, SGK1 CA mice demonstrated a significant increase in RA CV, APDs (50%, 70%, and 90%), and *dV/dt* as compared with WT mouse atria (Figure 7C and Supplemental Table 6).

To further interrogate SGK1 overactivation, we then utilized the HFD mouse model again. In this case, we fed WT and SGK1 CA mice an HFD for 6 weeks (Figure 7D), then proceeded with basic characterization as well as electrophysiologic and biochemical studies (Supplemental Figure 6). Again, telemetry was done and 8-hour periods (midnight to 8 am) were analyzed in WT and SGK1 CA mice. Although there were no spontaneous sustained arrhythmias in either group, there were significantly more PACs in the SGK1 CA mice as compared with the WT mice, again suggesting a role for SGK1 signaling in atrial electrical excitability (Figure 7E). During invasive electrophysiology studies, SGK1 CA mice demonstrated slowed AV nodal conduction, with a significantly longer Wenckebach cycle length and PR interval (Supplemental Table 7). Although there was no difference in the proportion of mice inducible for AF greater than 1 second, there was a significant increase in the number of inducible AF episodes in the SGK1 CA mice as compared with their WT counterparts, and a similar trend was seen in AF burden (Figure 7E). To better assess the general mechanism of arrhythmogenesis, optical mapping was performed on these 2 groups of mice. SGK1 CA left and right atria demonstrated an overall increase in APD as compared with the WT mouse atria, which met statistical significance at 70% and 90% repolarization (Figure 7F). Interestingly, CV was also generally higher in SGK1 CA mice as compared with WT mice, and this difference met statistical significance in the left atrium (Supplemental Table 8). Finally, qRT-PCR was done in these WT and SGK1 CA atria and revealed a significant increase in *Col1A1* and *Nlrp3* transcripts in CA mouse atria (Figure 7G). Together, our data suggest opposing effects of SGK1 activity on obesity-related AF, with SGK1 activation promoting development of AF coincident with increased fibrosis and inflammation, while SGK1 inhibition was protective against these pathologic changes.

## Discussion

Obesity is a reversible risk factor for AF, and the rate of obesity continues unabated at epidemic proportions. Prior work examining the link between obesity and AF has implicated fibrosis, connexin dysregulation, inflammation, and ion channel alterations in arrhythmogenesis. Here, we demonstrate marked alteration in atrial gene expression brought on by obesity and an obesity-induced upregulation of atrial SGK1 transcription, activation, and activity. SGK1 inhibition is associated with a reduction in spontaneous atrial ectopy as well as AF inducibility in a mouse model of HFD-induced obesity. This protective effect was associated with alterations in atrial electrophysiology as well as an attenuation in the obesity-related structural remodeling and inflammation. Overall, SGK1 inhibition prevents some of the pathologic effects of obesity, making it a necessary signaling pathway and an attractive therapeutic target in this AF model. We posit that SGK1 likely interacts with other obesity-related adverse signaling pathways and that inhibiting SGK1 can therefore mitigate the development of AF.

Despite the established role of obesity in cardiovascular disease, there are limited data regarding the transcriptional effects of obesity on the heart. In a similar mouse model, existing literature has shown that obesity-induced transcriptional changes in the left ventricle largely affect genes and pathways involved in cardiac metabolism, including glycolysis as well as remodeling (52). Here we present the effects of obesity on *atrial* gene expression, which in particular include genes involved in cardiac metabolism, cell-cell connectivity, cardiac remodeling, and cytokine signaling. Importantly, the sequencing data presented here supported our preexisting hypothesis that obesity may result in activation of SGK1-related pathways, and we demonstrated increased insulin signaling, mineralocorticoid signaling, and mTOR signaling.

While cardiac SGK1 activity may be protective in acute pressure overload (29), persistent activation during chronic pressure overload has been shown to be maladaptive (30). In models of metabolic syndrome, SGK1 signaling in hepatocytes (53) and adipocytes (54) has been shown to exacerbate insulin resistance. SGK1 is transcriptionally upregulated by circulating factors, including glucocorticoids, mineralocorticoids, and insulin (55). Given the latter, we suspected that obese mice, which are known to have glucose intolerance (56), may have increased SGK1 expression. We demonstrate here that in fact diet-induced obesity does increase both atrial and ventricular SGK1 transcription and signaling. This is a potentially novel finding in the heart but is an extension of existing literature, which demonstrates obesity-related increases in SGK1 signaling in the aorta (25), adipose tissue (26), and hippocampus (27).

From a macroscopic electrophysiologic perspective, we found a marked difference in inducibility of atrial arrhythmias in lean and obese mice. Despite this phenotype, there did not seem to be proportionate differences in surrogate electrophysiology parameters, including measures of tissue refractoriness and optical electrophysiologic parameters. We did find, however, inter-atrial differences in action potential characteristics and impulse propagation that were brought on by obesity but reversed with SGK1 inhibition. While this finding may contribute to AF phenotype, it is not independently sufficient to drive AF, as lean SGK1 CA mice — which develop these inter-atrial differences — are not inducible for AF. Further investigation will be necessary to determine the role of SGK1 signaling in defining atrial “sidedness.”

Obesity has been implicated in the development of both the triggers and substrate that underlie cardiac arrhythmias (6). There are discordant data regarding the effect of obesity on the cardiac sodium current (33, 57–60), and both gain-of-function (61) and loss-of-function (62) mutations in the *SCN5a* gene have been associated with AF in families. A particularly relevant recent study of mice with diet-induced obesity, for example, demonstrated decreased expression of the sodium channel subunit Na_V_1.5 associated with a decrease in *I_Na_* as well as APD shortening and decreased *dV/dt*_max_ in obese, as compared with lean, mouse left atria (35). These data would seem to stand in contrast to our findings, as we did not find an obesity-related reduction in Na_V_1.5 expression. However, despite the apparent contradiction, the same mouse model had 25% reduction in AF burden when treated with flecainide, an *I_Na_* blocker (63). A recent study confirmed the pro-AF potential of excess *I_Na_*, as it was shown to lead to atrial myopathy, remodeling, and arrhythmia (64). We did not specifically study the effects of obesity on atrial *I_Na_* (as compared with lean mice) but found that the protective effect of SGK1 inhibition in obese mice was associated with a depolarizing shift in the “window” current for *I_Na_* in the atria; this may be expected to decrease late *I_Na_* (34). This finding is consistent with prior literature showing a leftward hyperpolarizing shift in the “window” current as the mechanism for proarrhythmia in *constitutive* SGK1 activation (30). In the context of available literature, our results suggest that *I_Na_* regulation may be protective in obesity-related AF, but this only partially explains the benefit derived from SGK1 genetic inhibition.

Substantial data suggest a role for obesity-induced structural remodeling in the pathogenesis of AF. Proposed mechanisms include interstitial fibrosis (7, 8, 10), connexin dysregulation (11, 12), and even fatty infiltration (7). SGK1 activity in particular is associated with a number of fibrosing conditions throughout the body and may be stimulated by TGF-β activity (65). Here we demonstrate the protective effect of SGK1 inhibition in the expression of several profibrotic factors, including collagen, CTGF, and α–smooth muscle actin. Since we did not find a significant difference in histologic fibrosis, it is possible that the transcriptional/translational changes are a more sensitive assay or may be early signs of fibrosis, but we cannot draw any firm conclusions (66). MMPs and TIMPs have generally antagonistic functions and work in concert to maintain the homeostatic balance of the ECM (37, 38). The relative changes seen here with obesity, which mimic those seen in aged, frail mouse atria (39), may suggest a profibrotic transcriptional programming. Finally, the role of SGK1 signaling on cardiomyocyte connexin expression is not described in literature, but there are extensive data regarding the effects of fibrosis and inflammation on connexin expression. Our data are consistent with a secondary effect of fibrosis and inflammation on connexin expression, rather than a primary effect of SGK1 regulation.

The protective effects of SGK1 genetic inhibition are consistent with data examining the effects of cardiomyocyte mineralocorticoid receptor knockout (67). Despite the protective effects of SGK1 inhibition on atrial and ventricular fibrotic signaling, detailed echocardiographic assessment of systolic and diastolic function did not reveal any notable effects of obesity or SGK1 inhibition. Thus, importantly, the protective effect of SGK1 inhibition on AF inducibility in this model is unlikely to derive from hemodynamic effects or effects on LA pressure secondary to the beneficial effects of SGK1 on ventricular remodeling, but rather suggest a primary beneficial effect of SGK1 inhibition on AF pathogenesis.

SGK1 activation has been linked to inflammation via both the NLRP3 inflammasome as well as NF-κB signaling. With respect to the latter, SGK1 directly phosphorylates IκB kinase alpha, resulting in increased NF-κB activity (49). SGK1-dependent NF-κB activation has been demonstrated in renal collecting ducts (68) and aortic vascular smooth muscle cells (69). Recent literature has posited cardiomyocyte NLRP3 overexpression as a sufficient trigger for AF (14), and our data regarding atrial NLRP3 expression are compelling in this context. Prior studies have demonstrated an association between SGK1 and inflammasome activity, but the exact mechanism has not been clearly delineated. In a model of hypoxia-induced pulmonary arterial hypertension, SGK1 activity was shown to be associated with hypoxic pulmonary macrophage infiltration, whereby knockout of SGK1 reduced macrophage content (70). Meanwhile, a specific SGK1 inhibitor was shown to reduce NLRP3 inflammasome expression and mitigate angiotensin II–induced cardiac inflammation and fibrosis (32). In our model, obesity resulted in a marked elevation in IL-1, a cytokine frequently cited as proarrhythmic, which was prevented by SGK1 inhibition, perhaps through its effects on the NF-κB and NLRP3 axes.

An interesting aspect of this study to take note of — as in the Das et al. (30) study regarding TAC-induced cardiac remodeling — is that our model of SGK1 knockdown is restricted to cardiomyocytes via the MHC promoter. Yet, the effects seemingly extend to functions classically thought to be driven by noncardiomyocytes; ECM maintenance is generally attributed to activated fibroblasts and inflammation to monocytes. However, in vitro, cardiomyocytes have been shown to be capable of expressing fibrosis-related transcripts (71) and even do so in response to leptin exposure (72). Murine in vivo models of cardiac fibrosis due to pressure overload have demonstrated an essential role for cardiomyocyte-dependent TGF-β signaling (73). There are limited data regarding the role of cardiomyocyte-derived signaling in inflammation, but cardiomyocyte calmodulin kinase — through both NLRP3 and NF-κB — is essential to pressure overload–induced cardiac inflammation (74, 75). A particularly relevant study from Rickard et al. (67) demonstrated cardiomyocyte mineralocorticoid receptors as essential in mineralocorticoid- and salt-induced cardiac fibrosis and inflammation. This is of critical interest in the context of our data, as the SGK1 axis lies downstream of mineralocorticoid receptor signaling via transcriptional regulation.

We acknowledge several limitations in our study. While bulk RNA sequencing is valuable in identifying perturbed pathways through differences in gene expression, it does not account for the complex cellular composition of cardiac tissue and the changes therein that ultimately affect gene expression. Future experiments assessing transcription at the single-cell level in the heart of obese animals may yield interesting information on the role of different cardiac cells in mediating AF pathogenesis. Human obesity is often associated with hypertension and hypertensive heart disease, but we did not find any evidence of either with tail cuff BP and with echocardiographic assessment of BP. We concede that these approaches have their limitations and may not be as sensitive as cardiac MRI, strain imaging, and/or invasive radio telemetry (76, 77). However, we suspect that the short duration of HFD feeding may not result in increased afterload and that the cardiomyocyte-specificity of SGK1 knockdown would minimize any possible confounding effects of hypertension or vascular resistance in our study. At first glance, it would seem that the stark differences in AF inducibility caused by SGK1 inhibition are incompletely explained by the less pronounced mechanistic differences. We propose that SGK1 inhibition, through its pleiotropic effects, decreases AF susceptibility via multiple contributory mechanisms. Another limitation innate to rodent studies of AF is that a “positive” finding typically represents very brief (on the order of seconds) episodes of atrial tachyarrhythmia that are induced in vivo; i.e., these episodes are neither spontaneous nor sustained. We would argue that mouse models of AF are reasonable models of the substrate, with the trigger provided by pacing/stimulation. Finally, pharmacological inhibition of endogenous SGK1 in this model would have provided complementary data to augment our hypothesis that SGK1 inhibition may be a novel approach to obesity-related AF, but poor bioavailability, potency, and specificity of commercially available SGK1 inhibitors currently limit their utility.

In summary, this study suggests that targeting of SGK1 should be further investigated for its therapeutic potential in obesity-related AF. Through bulk RNA sequencing, we reveal the marked effects of obesity on the atrial transcriptome and describe a number of paths that may lead from obesity to AF beyond ion channel alterations. Notably, SGK1 activity seems to be required for many of these proarrhythmic pathways, suggesting a central role for this kinase in obesity-related AF, and thus making inhibition a potentially attractive target for intervention. Future work will include assessing the role of SGK1 inhibition in other AF-related stressors known to activate SGK1 signaling, such as hypertension, and assessment of pharmacologic SGK1 inhibition.

## Methods

The Supplemental Methods section includes detailed descriptions of the following procedures/techniques: transthoracic echocardiography, tail cuff blood pressure, IPGTT, immunoblotting, flow cytometry, histology and fibrosis quantification, and cytokine ELISAs. The RNA-sequencing data have been uploaded to the NCBI Gene Expression Omnibus database with free accessibility (accession GSE211229).

### Animal studies.

SGK1 DN and CA mice were generated as previously described, with cardiac specific expression of a CA (S422D) or DN (K127M) SGK1 transgene driven by the α-myosin heavy chain promotor (30). Cardiac specificity was confirmed by immunoblotting for an incorporated hemagglutinin epitope tag (Supplemental Figure 7A). Transgene-carrying mice were bred with WT C57BL/6J mice (obtained from The Jackson Laboratory) to generate litters of transgenic and WT mice. Mice were genotyped by PCR for the presence of the transgene using tail DNA and the following primers, obtained from Eton Bioscience Inc.: forward 5′-GGTAGCAATCCTCATCGCTTTC-3′, reverse 5′-CTTCAGGGTGTTTGCATGCA-3′ (Supplemental Figure 7B). Starting at 6 weeks of age, the SGK1 DN and WT littermates were fed an HFD (Research Diets D12492, 60% fat by calories) for at least 10 weeks and up to 14 weeks until body weight was at least 34 g. The weight cutoff was approximately the 95th weight percentile among mice fed a control diet and was used to exclude mice that may be resistant to diet-induced obesity (78). Lean mice were fed a standard control chow. Electrophysiology studies were performed under general anesthesia and terminated with extraction of the heart. Optical mapping and biochemical analyses required heart extraction, which was performed under deep anesthesia.

### Transthoracic echocardiography.

Transthoracic echocardiograms were performed in anesthetized mice using a Vevo 3100 high-resolution Imaging System coupled to an MX250s ultra-high frequency linear array transducer (15–30 MHz, center transmit: 21 MHz, axial resolution: 75 μm) (both FUJIFILM VisualSonics) as described previously (79, 80). A detailed description is available in the Supplemental Methods section.

### Electrophysiology studies.

Electrophysiology studies were performed under general anesthesia induced by administering 5% isoflurane driven by an oxygen source into an induction chamber. Anesthesia was subsequently maintained with 1% to 2% isoflurane in 95% O_2_. A warming blanket was used to maintain a rectal temperature of 35°C to 37°C to avoid hypothermia. An octapolar catheter (EPR-800, Millar) was inserted into the right jugular vein and positioned in the right atrium and ventricle; the position was ascertained with continuous monitoring of the intracardiac electrograms. Sinus node function was determined by measuring the SNRT following 30 seconds of pacing at 3 cycle lengths (120, 100, and 80 ms). The Wenckebach cycle length was determined with progressively faster atrial pacing rates. Atrial, ventricular, and AV nodal refractory periods were measured using programmed electrical stimulation with overdrive pacing trains at 100 ms followed by single extrastimuli. Retrograde conduction Wenckebach cycle length was measured by pacing at progressively faster ventricular pacing rates. Provocative testing for arrhythmia induction was performed with double extrastimuli (S1-S2-S3) at 2 S1 cycle lengths (120 and 100 ms) and progressively decreasing S2 and S3 to 10 ms. Additionally, rapid burst pacing was performed at gradually faster rates (starting at 50 ms) to a pacing cycle length of 20 ms and lasting 3 seconds and 6 seconds. AF was defined as a rapid atrial rhythm with atrial rate greater than ventricular rate and irregular ventricular response (R-R intervals). The duration of AF was measured from the end of the pacing train to the end of the rapid atrial activity. Mice were considered inducible if they had at least 1 episode of AF longer than 1 second.

### Telemetry.

Continuous ambulatory monitoring in obese WT and SGK1 DN mice was achieved with implanted wireless telemetry devices (ETA-F10 transmitter, Data Sciences International) as previously described (81). Under general anesthesia, telemetry devices were implanted into the abdominal cavity, and electrodes were tunneled to a modified lead II position. Telemetry recordings were performed continuously for 3 months. Data analysis was performed using LabChart 8 (AD Instruments).

### Optical mapping.

Isolation and perfusion of the heart were performed as previously described (82, 83). Briefly, the mouse was anesthetized using isoflurane, the heart was excised and perfused via an aortic cannula, and the cannulated heart was perfused with a modified Tyrode solution (128.2 mM NaCl, 4.7 mM KCl, 1.19 mM NaH_2_PO_4_, 1.05 mM MgCl_2_, 1.3 mM CaCl_2_, 20.0 mM NaHCO_3_, 11.1 mM glucose; pH 7.35 ± 0.05) using a Langendorff perfusion setup. Blebbistatin (10 mM, Tocris Bioscience, Bio-Techne) was used to arrest cardiac motion. The heart was stained for 30 minutes with a voltage-sensitive dye (di-4-ANEPPs, 2 mmol/L in dimethyl sulfoxide, obtained from Invitrogen). Custom-made epicardial platinum electrodes and a Medtronic stimulator were used to pace the heart. Pacing was performed at 60 to 120 ms cycle lengths at twice the capture threshold (4 ms square wave stimuli). A halogen light source (X-Cite 150 W, filtered at 520 ± 45 nm) was used to excite fluorescence. Emissions greater than 610 nm were collected and focused onto an 80 × 80 charge-coupled device camera (RedShirt Imaging SMQ Camera and Macroscope IIA) using a 50 mm original magnification ×2.7 lens (numerical aperture 0.4). Data sampling was performed at 2000 frames per second with a filter setting of 1 kHz. A specifically designed Matlab program was used to perform data analysis in order to generate conduction velocities and APD at 50%, 70%, and 90% repolarization.

### Cardiomyocyte isolation.

Mice were anesthetized as described above and injected intraperitoneally with 0.2 cc heparin. Hearts were then dissected and mounted on a Langendorff system (Radnoti) via aortic cannulation. Hearts were perfused with Ca^2+^-free normal Tyrode’s solution containing NaCl 137 mM, KCl 4 mM, MgCl_2_ 1 mM, HEPES 10 mM, NaH_2_PO_4_ 0.33 mM, taurine 5 mM, and dextrose 10 mM (pH 7.4), followed by the same perfusion buffer containing collagenase D and B (0.48 mg/mL and 0.36 mg/mL, respectively; both from Roche) and protease XIV (0.06 mg/mL; MilliporeSigma) at 37˚C. After enzymatic digestion, the LA appendage was dissected into Kraftbrühe buffer containing KCl 25 mM, KH_2_PO_4_ 10 mM, dextrose 20 mM, dl-aspartic acid potassium salt 10 mM, BSA 0.1%, l-glutamic acid potassium salt 100 mM, MgSO_4_ 2 mM, taurine 20 mM, EGTA 0.5 mM, creatine 5 mM, and HEPES 5 mM (pH 7.2). Single LA cardiomyocytes were dispersed by triturating the digested tissue and seeded on laminin-coated 8 mm coverslips. After 1-hour incubation at 37°C in a 5% CO_2_ incubator, cardiomyocytes were treated with a gradually increased Ca^2+^ concentration from 0.06 mM to 1.2 mM in normal Tyrode’s solution at room temperature (RT).

### Patch-clamping.

Sodium currents (*I_Na_*) were recorded by whole-cell patch-clamp techniques in isolated murine LA cardiomyocytes at RT using an established protocol (84). Borosilicate-glass electrodes with tip resistances 1.5 to 3 MΩ were filled with pipette solution. For *I_Na_* recordings, pipette solution contained CsCl 135 mM, NaCl 10 mM, CaCl_2_ 2 mM, EGTA 5 mM, HEPES 10 mM, MgATP 5 mM (pH 7.2 with CsOH); bath solution contained NaCl 50 mM, CaCl_2_ 1.8 mM, MgCl_2_ 1 mM, CsCl 110 mM, dextrose 10 mM, HEPES 10 mM, and nifedipine 0.001 mM (pH 7.4 with CsOH). Currents were low-pass-filtered at 5 kHz with an Axopatch 200B amplifier and digitized at 10 kHz with a Digidata 1440A A/D converter. The *I_Na_* was obtained with 50 ms depolarizations (–100 mV to +40 mV) from a holding potential of –120 mV at 0.1 Hz. Data were recorded with Clampex 10.3 and analyzed with Clampfit 10.3 (Molecular Devices). Cell capacitance and series resistance were compensated by about 70%. Leakage compensation was not used. Currents were normalized to cell capacitance. (Obese SGK1 WT, 56 ± 7 pF, *n* = 7; obese SGK1 DN, 56 ± 4 pF, *n* = 8; *P* = NS.) The *I_Na_* reversal potential was derived by linear regression of the positive slope of the current-voltage relation: SGK1 WT, 37 ± 7 mV, SGK1 DN, 44 ± 7 mV; both were close to the Na^+^ reversal potential by the Nernst equation (~40 mV).

### RT-qPCR of RNA.

Tissue samples were snap-frozen and then lysed using a Tissue Lyser (QIAGEN) in TRIzol (Thermo Fisher Scientific) followed by RNA extraction. A total of 500 ng of RNA was reverse-transcribed using the High Capacity cDNA Reverse Transcription Kit (Thermo Fisher Scientific). RT-qPCR was conducted with SsoAdvance Universal SYBR Green (Bio-Rad) using a QuantStudio 6 Flex Real-Time PCR System (Thermo Fisher Scientific). Quantification of all target gene expression was normalized to β-actin. Primers for genes of interest are presented in Supplemental Table 9.

### RNA sequencing.

RNA isolation and reverse transcription were carried out as described above. Three samples each of lean and obese mouse atria were used to extract RNA, and quality was assessed with the RNA 6000 Pico assay kit using the Agilent Bioanalyzer. Sequencing-ready cDNA libraries were prepared using the NEBNext Ultra RNA Directional Library Prep Kit for Illumina following the manufacturer’s protocol. Bioanalyzer traces were used to confirm library size distribution. The libraries were quantified by quantitative PCR using the KAPA Library Quantification Kit (Roche) and then sequenced as single-end 50 bp reads on an Illumina HiSeq 2000 in high-output mode.

The raw sequence image files from the sequencer were converted to the fastq format and checked for quality to ensure the sequencing quality scores did not deteriorate at the read ends. Useful metrics, such as the per-base sequence quality, per-base sequence content, per-base N content, sequence length distribution, adapter and k-mer content, and sequence duplication levels, were collected for each sequencing run using fastqc (85). At this stage, any samples that did not pass QC were flagged and investigated later. The fastq files were then aligned to the mouse genome (GRCm38, ENSEMBL version 79) using STAR (86) aligner. The alignment files in the form of bam files were indexed for the ability to view them on Integrative Genome Viewer (87). Alignment statistics, such as average read length, percentage of uniquely mapped reads, percentage of multi-mapped reads, and number of splice junctions, were collated. Only reads that were uniquely mapped were considered for read annotation to gene features. Reads were annotated with the ENSEMBL 79 gene transfer format (gtf) file using *S*almon (88). Salmon provides both read counts and transcripts per million reads for each gene feature in the ENSEMBL gtf. The R package DESeq2 (89) was used for differential gene expression analysis. Genes were considered differentially expressed if they were upregulated by log_2_FC > +1 or downregulated by log_2_FC < –1 with an FDR < 0.05 (FC, fold change of average counts per million; FDR, false discovery rate).

Pathway analysis was performed using GSEA (90, 91) (GSEA preranked mode) using the KEGG pathway database. For all genes included in the differential expression analysis using DESeq2 (previously described), a metric was computed as the product of log_2_FC and –log_10_(*P* value). The genes were ranked by this metric, resulting in the most significantly overexpressed genes at the top of the list and the most significantly underexpressed genes at the bottom. A “running sum” statistic was calculated for each gene set in the pathway database, based on the ranks of the members of the set, relative to those of the nonmembers. The enrichment score (ES) was defined as the maximum sum of the running sum, and the genes that made up this maximum ES contributed to the core enrichment in that pathway. All pathways with an FDR < 0.25 were considered significant.

### Statistics.

Data are reported as mean ± SD, unless specifically noted otherwise. All statistical analyses were conducted with GraphPad Prism software. Animal group sizes were as low as possible. Categorical variables with binary outcomes were compared with Fisher’s exact test. The Shapiro-Wilk test of normality was utilized, and normality was rejected for *P* < 0.01. For a comparison of 2 groups, a 2-tailed Student’s *t* test (for normally distributed data) or a 2-tailed Mann-Whitney *U* test (where normality was rejected) were used to determine statistical significance. One-way ANOVA was performed, followed by post hoc Dunnett’s test, for comparisons of 3 normally distributed groups. For comparisons of 3 groups where normal distribution could not be assumed, a Kruskal-Wallis test was performed, and if significant, post hoc Dunn testing was performed. A *P* < 0.05 was used to denote statistical significance.

### Study approval.

All studies were approved by the Institutional Animal Care and Use Committee at Massachusetts General Hospital and were in accordance with the NIH *Guide for the Care and Use of Laboratory Animals* (National Academies Press, 2011).

## Author contributions

AB, SD, and DM conceived the studies; AB, MN, AR, PTE, SD, and DM designed the experiments; AB, GL, LX, AY, MH, JG, MY, MJS, YI, JT, and XY performed the experiments; AB, GL, LX, AY, and MH analyzed the data; and AB, SD, and DM wrote the manuscript.

## Supplementary Material

Supplemental data

## Figures and Tables

**Table 1:**
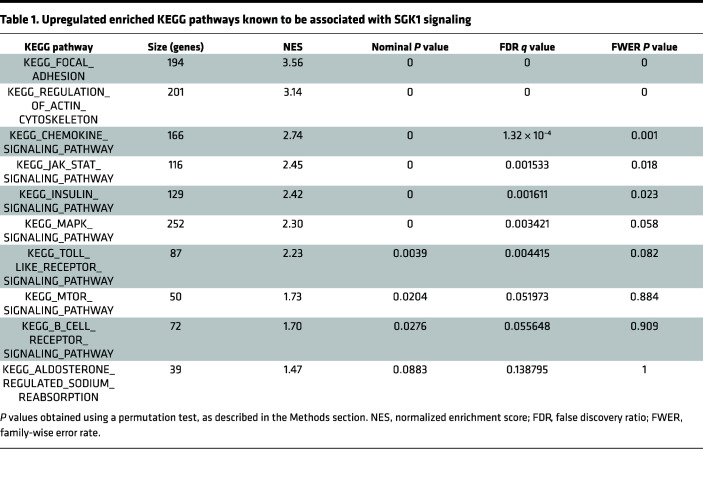
Upregulated enriched KEGG pathways known to be associated with SGK1 signaling

**Table 2:**
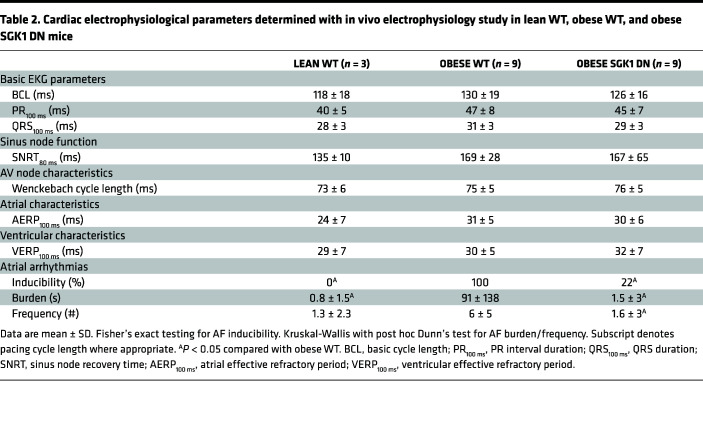
Cardiac electrophysiological parameters determined with in vivo electrophysiology study in lean WT, obese WT, and obese SGK1 DN mice

**Table 3:**
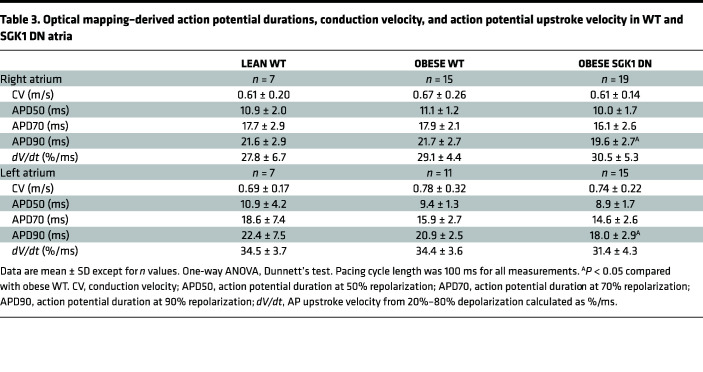
Optical mapping–derived action potential durations, conduction velocity, and action potential upstroke velocity in WT and SGK1 DN atria

## Version 1 08/23/2022

In-Press Preview

## Version 2 10/10/2022

Electronic publication
