# Supplementary material for: Genetic inhibition of serum glucocorticoid kinase 1 prevents obesity-related atrial fibrillation
Source: JCI Insight. 2022 Oct 10;7(19):e160885. doi: 10.1172/jci.insight.160885 (PMC9675459; doi:10.1172/jci.insight.160885)
Supplement: Supplemental data [file jciinsight-7-160885-s159.pdf]

SUPPLEMENTARY MATERIALS

Supplemental Methods

Transthoracic Echocardiography

Transthoracic echocardiograms were performed in anesthetized mice using a Vevo® 3100 high-resolution Imaging System coupled to a MX250s ultra-high frequency linear array transducer (15-30 MHz, center transmit: 21 MHz, axial resolution: 75 µm) (both FUJIFILM VisualSonics, Toronto, Ontario, Canada) as described previously.(79, 80) Briefly, mice were exposed to 3% isoflurane (Baxter International, Deerfield, USA) and fixed in a dorsal position on a heating pad to maintain physiological body temperatures. Left ventricular systolic function was measured using 2D areas in the parasternal long axis view. Dimensions were measured with M-mode imaging of the mid-chamber in short axis. Tissue velocity profiles were measured in the apical four chamber view at the septal mitral annulus. Trans-mitral flow patterns were recorded in the apical four chamber view by pulsed-wave Doppler using color doppler guidance to identify the max. flow rates. All acquired images were digitally stored in raw format (DICOM) for further offline analyses. Image analyses were performed using the dedicated software package VevoLAB Version 3.1.0 (FUJIFILM VisualSonics).

Tail-Cuff Blood Pressure

Systolic, diastolic, and mean arterial pressures (SBP, DBP, MAP) were measured using a validated tail-cuff method that relies on volume pressure recording technology (Kent Scientific Corporation). Blood pressure measurements were made by using ten acclimation cycles followed by fifteen measurement cycles; the latter were averaged.

Intra-peritoneal Glucose Tolerance Testing

Mice were fasted for 6 hours in the morning, immediately following the dark cycle. After the fast, they were intraperitoneally injected with glucose at 2g/kg body weight. Glucose was measured using a commercially available glucometer (Contour Next EZ, Bayer) at baseline, and then at 15, 30, 60, and 120 minutes after glucose injection. Glucose levels were plotted against time, and using Graphpad Prism, area under the curve (AUC) could be measured.

Immunoblotting

Western blot analysis was performed on snap-frozen atrial tissue. Tissue samples were lysed using a Tissue Lyser (Qiagen) in protein lysis buffer supplemented with protease and phosphatase inhibitors (Boston BioProducts). Protein content was measured using a commercially available kit (DC Protein Assay, Bio-Rad). Equal amounts (15-25 μg) were treated with Laemmli buffer and β-mercaptoethanol and incubated at 100° C for 5 minutes. The lysate was then electrophoresed on a 4-20% SHS-polyacrylamide resolving gel and transferred to a PVDF membrane. The membrane was incubated overnight at 4° C with relevant antibodies, and then hybridization was completed with secondary antibody for 2 hours at room temperature. Antibody signal detection was achieved by employing the Clarity Western ECL Substrate (BioRad #1705061). Imaging and image quantification were done via BioRad Chemidoc Touch Imaging System and ImageLab, respectively. A detailed list of antibodies is provided in supplemental table 10.

Flow Cytometry

Mice were perfused through the left ventricle with 10 mL of ice-cold PBS. Hearts were excised and atrial tissue was micro-dissected using a dissection microscope. After harvest, tissues were minced into small pieces and subjected to enzymatic digestion with 450 U/mL collagenase I, 125 U/mL collagenase XI, 60 U/mL DNase I and 60 U/mL hyaluronidase (all Sigma-Aldrich) for 40 minutes at 37°C under agitation. Tissues were then triturated, and cells were filtered through a 40-µm nylon mesh, washed and centrifuged to obtain single-cell suspensions. For a myeloid cell staining on processed heart samples, isolated cells were stained at 4°C in FACS buffer (PBS supplemented with 0.5% BSA) with mouse hematopoietic lineage markers including PE-conjugated anti-mouse antibodies directed against B220 (clone RA3-6B2, 1:600), CD49b (clone DX5, 1:1200), CD90.2 (clone 53–2.1, 1:3000), CD103 (clone 2E7, 1:600), Ly6G (clone 1A8, 1:600), NK1.1 (clone PK136, 1:600), and Ter-119 (clone TER-119, 1:600). This was followed by a second staining for CD11b (clone M1/70, 1:600), CD45 (clone 30-F11, 1:600), F4/80 (clone BM8, 1:600) and Ly6C (clone HK1.4, 1:600). DAPI was used as a cell viability marker. Neutrophils were identified as CD45highCD11bhigh(B220/CD49b/CD90.2/CD103/NK1.1/Ter-119)lowLy6Ghigh and cardiac macrophages as CD45highCD11bhigh(B220/CD49b/CD90.2/CD103/Ly6G/NK1.1/Ter-119)lowF4/80highLy6Clow/int. Antibodies were purchased from BioLegend and BD Biosciences. Data were acquired on an LSRII (BD Biosciences) and analyzed with FlowJo software.

Histology and Fibrosis Quantification

Mice hearts were dissected and perfused with PBS. Hearts were then embedded in Tissue-Tek O.C.T. compound (Sakura Finetek), snap-frozen in 2-methylbutane on dry ice and sectioned into 10 µm slices using CryoJane Tape-Transfer System (Leica). Masson's trichrome stain for cardiac

fibrosis was performed according to the manufacturer's instructions (Sigma). Quantification of fibrosis in the atria was performed using the BZX Analyzer software.

Cytokine ELISAs

Cardiac puncture was performed during heart extraction to collect plasma. Commercially available kits were utilized to determine levels of IL-6 and CRP (R&D systems).

Supplemental Figures

Supplemental Figure 1: Dose response of HFD-induced SGK1 activation. ***A,*** Body weights for mice fed HFD for 6 weeks versus 10 weeks. ***B,*** Relative protein expression of phosphorylated and total SGK1 as well as downstream SGK1 targets NDRG1 and GSK3β and representative blots shown below. Unpaired Student's t-test. *P<0.05.

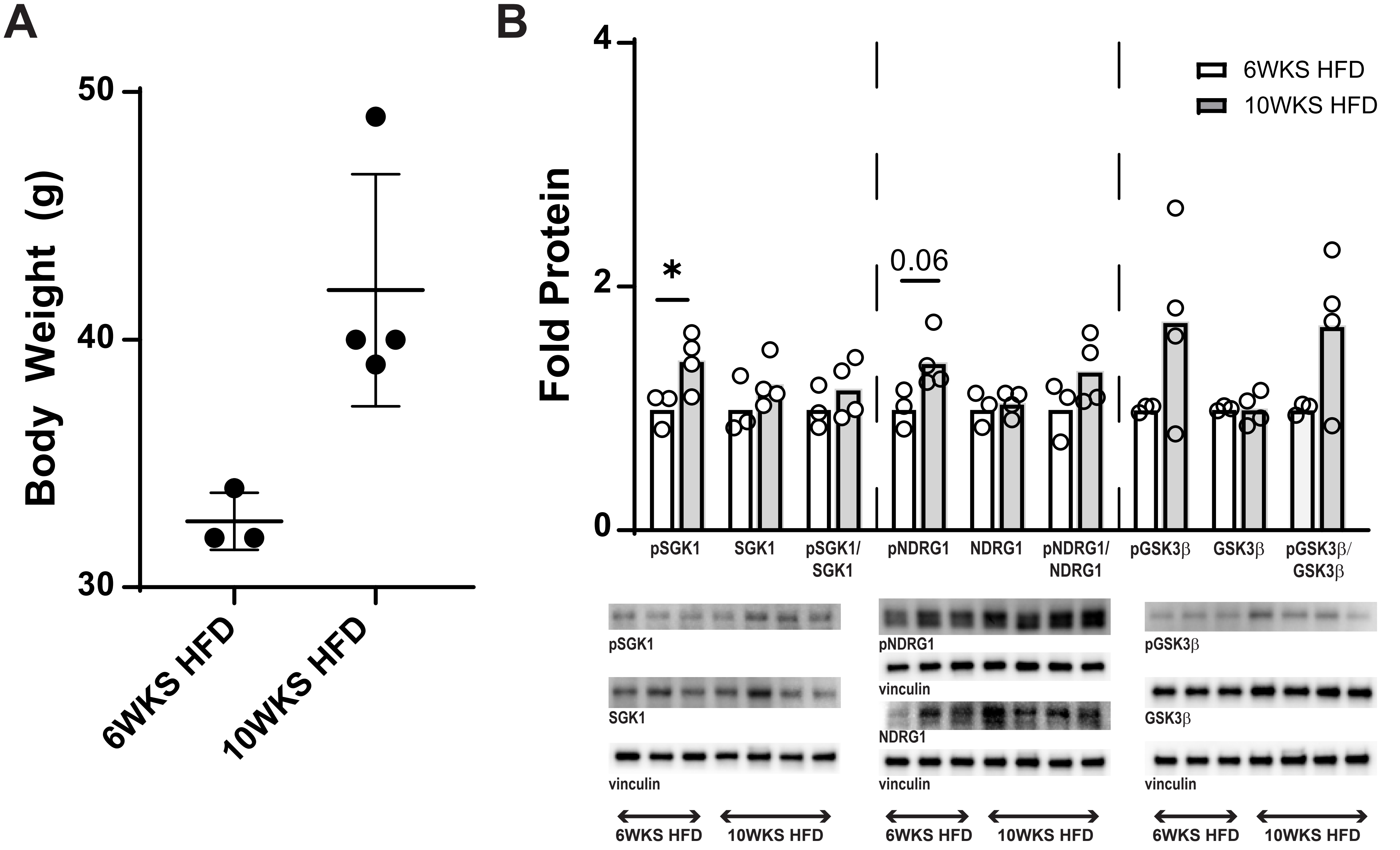
A
B
Body Weight (g)
50
40
30
6WKS HFD
10WKS HFD
Fold Protein
4
2
0
6WKS HFD
10WKS HFD
*
0.06
pSGK1
SGK1
pSGK1/SGK1
pNDRG1
NDRG1
pNDRG1/NDRG1
pGSK3β
GSK3β
pGSK3β/GSK3β
pSGK1
SGK1
vinculin
pNDRG1
vinculin
NDRG1
vinculin
pGSK3β
GSK3β
vinculin
6WKS HFD
10WKS HFD
6WKS HFD
10WKS HFD
6WKS HFD
10WKS HFD

Supplemental Figure 2: Characterization of lean WT, obese WT and obese SGK1 DN mice. *A* Mouse body weight during HFD or control chow feeding. ***B*** Heart weight and heart weight indexed to tibial length (HW/TL) in mice. ***C*** Systolic (SBP), diastolic (DBP), and mean arterial (MAP) pressure measured via tail-cuff. ***D*** Transthoracic echocardiography measured cardiac structural/functional parameters in lean and obese mice. LVEDD, left ventricular end diastolic diameter. ***E*** Blood glucose levels measured after intra-peritoneal glucose tolerance test. One-way ANOVA, Dunnett's test. *P<0.05 for comparison of lean WT (versus obese WT). No significant differences between the two obese groups. ***F*** Ambulatory Holter recording derived heart rate (HR) and root mean square of the successive differences (RMSSD) obtained over 8-hour periods.

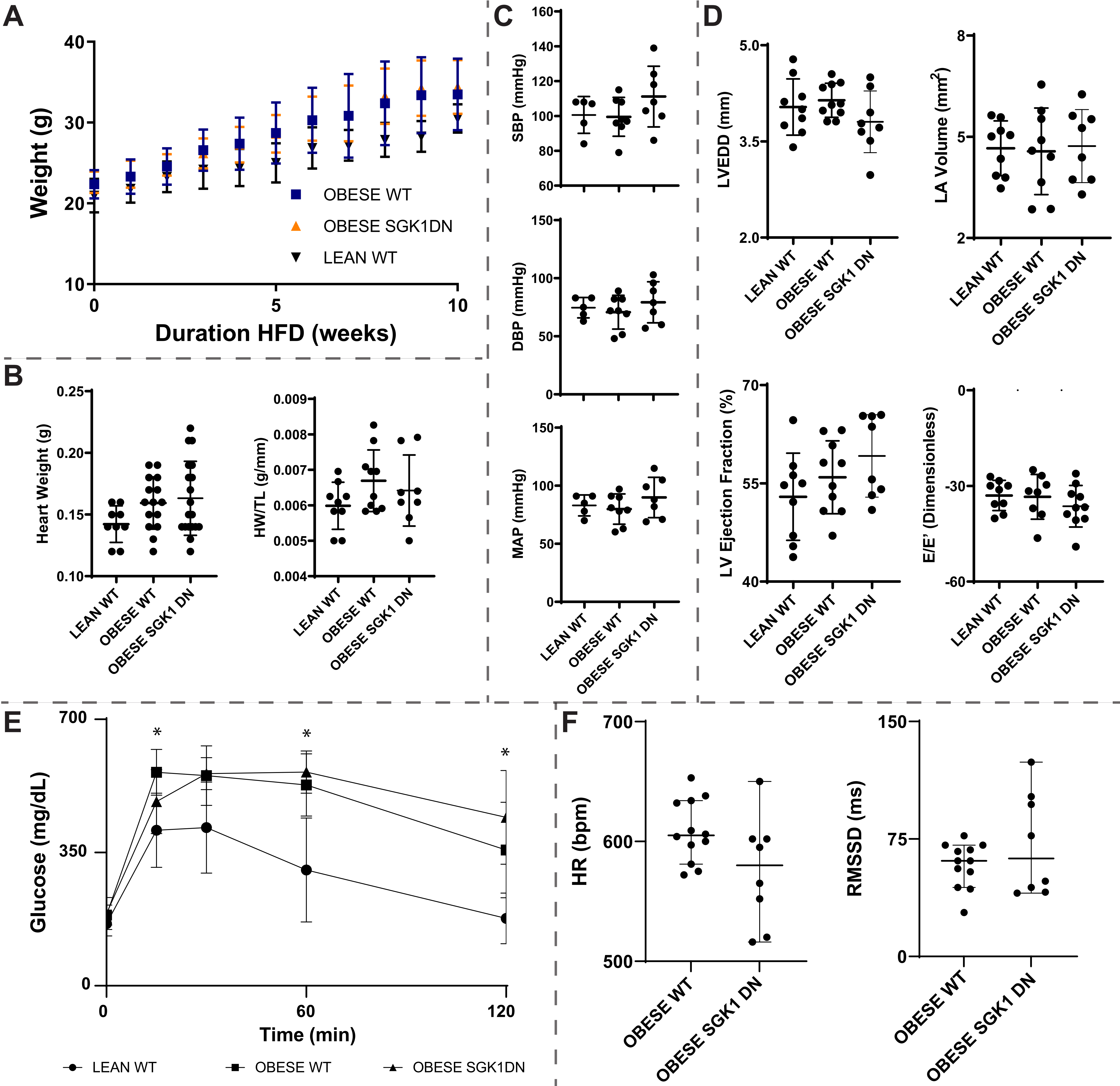

A
Weight (g)
Duration HFD (weeks)
OBESE WT
OBESE SGK1DN
LEAN WT
B
Heart Weight (g)
HW/TL (g/mm)
LEAN WT
OBESE WT
OBESE SGK1 DN
C
SBP (mmHg)
DBP (mmHg)
MAP (mmHg)
D
LVEDD (mm)
LA Volume (mm2)
LV Ejection Fraction (%)
E/E' (Dimensionless)
E
Glucose (mg/dL)
Time (min)
LEAN WT
OBESE WT
OBESE SGK1DN
F
HR (bpm)
RMSSD (ms)
OBESE WT
OBESE SGK1 DN

Supplemental Figure 3: SGK1 target phosphorylation in lean WT, obese WT, and obese SGK1 DN atrial tissue. *This data is also presented in Figure 1F, but here includes SGK1 DN atria.* Relative expression of phosphorylated and total SGK1, NDRG1, and GSK3β in lean WT, obese WT, and obese SGK1 DN mouse atria. Representative blots shown below. One-way ANOVA, Dunnett's test. *$P<0.05$, ***$P<0.001$.

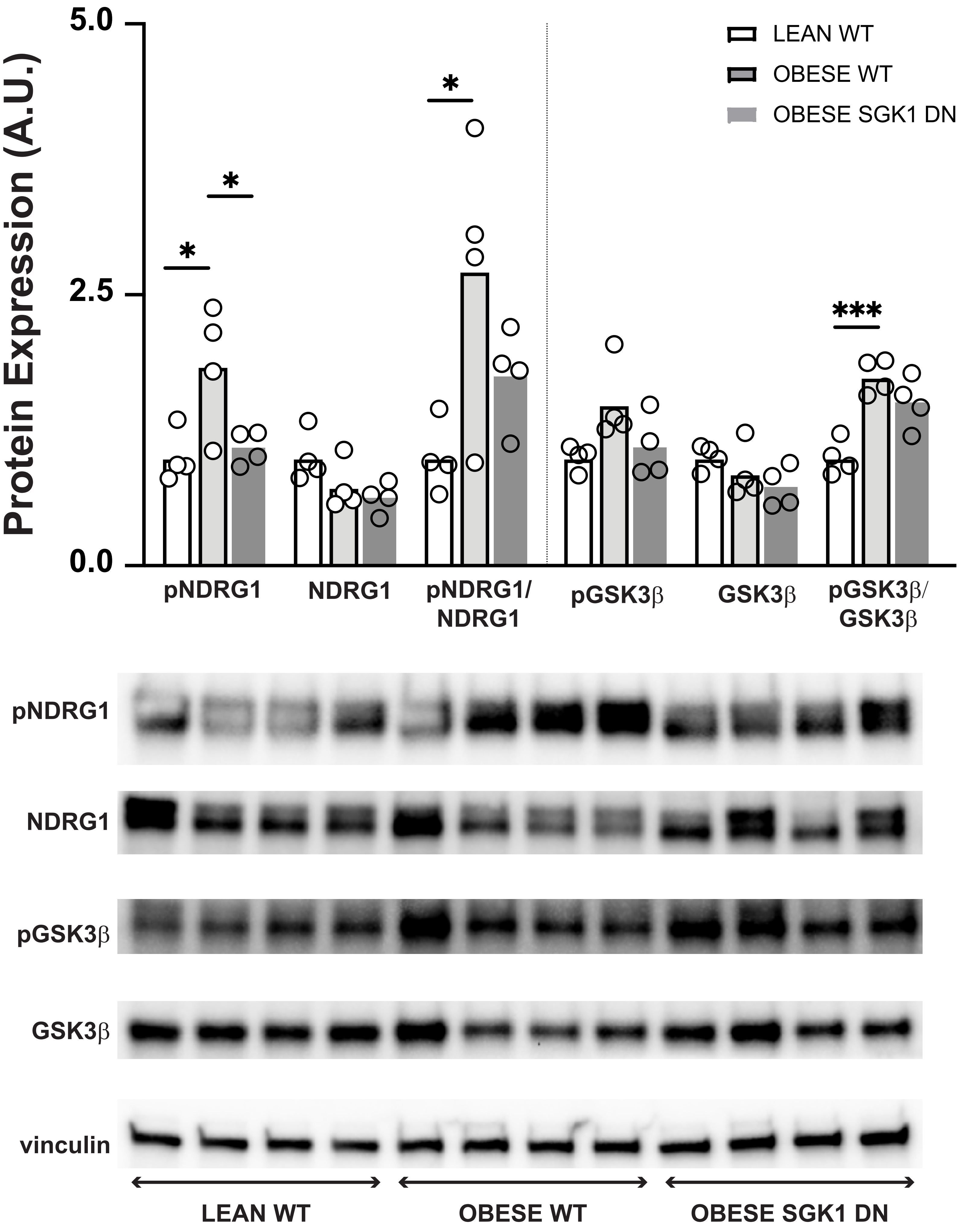

Protein Expression (A.U.)
5.0
2.5
0.0
LEAN WT
OBESE WT
OBESE SGK1 DN
*
*
*
***
pNDRG1
NDRG1
pNDRG1/NDRG1
pGSK3β
GSK3β
pGSK3β/GSK3β
pNDRG1
NDRG1
pGSK3β
GSK3β
vinculin
LEAN WT
OBESE WT
OBESE SGK1 DN

Supplemental Figure 4: Masson-trichrome staining for fibrosis. A Representative histologic sections of obese WT (above) and obese SGK1 DN (below) mouse hearts used for fibrosis quantification. **B** Atrial fibrosis, by % area, based on Masson-Trichrome staining. **C** Ventricular fibrosis, by % area, based on Masson-Trichrome staining.

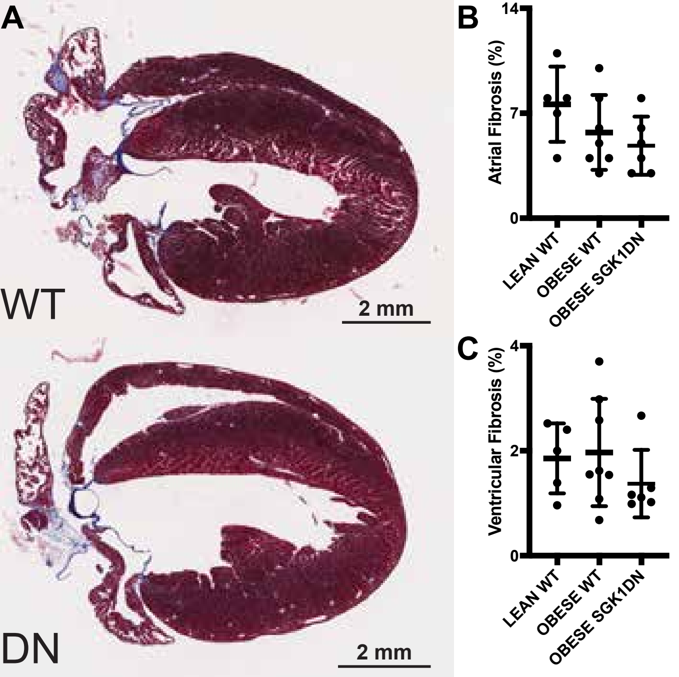
A
WT
2 mm
DN
2 mm
B
Atrial Fibrosis (%)
14
7
0
LEAN WT
OBESE WT
OBESE SGK1DN
C
Ventricular Fibrosis (%)
4
2
0
LEAN WT
OBESE WT
OBESE SGK1DN

Supplemental Figure 5: Plasma markers of systemic inflammation. ***A*** Plasma levels of C-reactive protein (CRP). ***B*** Plasma levels of interleukin 6 (IL-6).

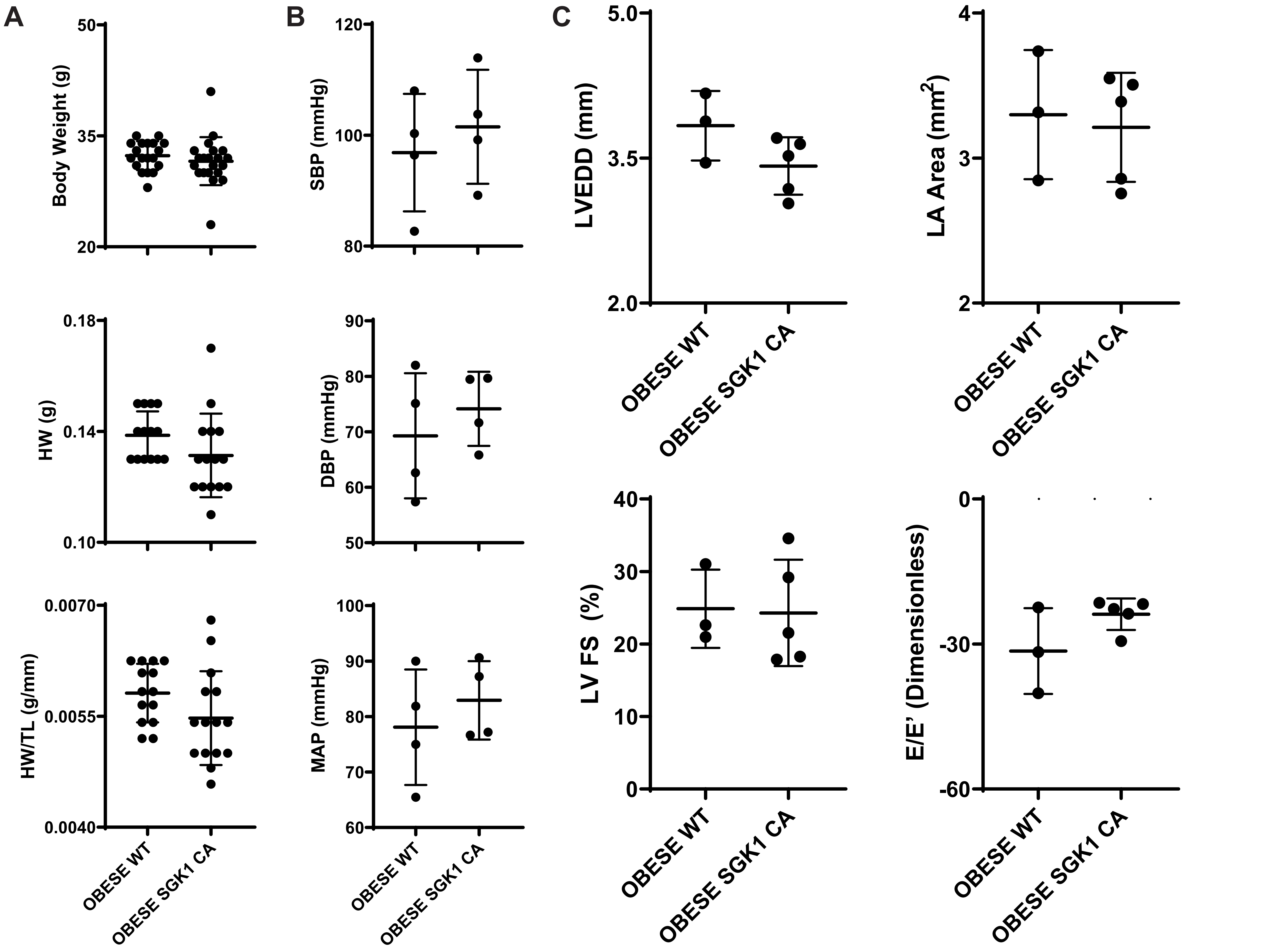
A
B
C
Body Weight (g)
HW (g)
HW/TL (g/mm)
SBP (mmHg)
DBP (mmHg)
MAP (mmHg)
LVEDD (mm)
LA Area (mm2)
LV FS (%)
E/E' (Dimensionless)
OBESE WT
OBESE SGK1 CA

Supplemental Figure 6: Characterization of obese WT and obese SGK1 CA littermates. ***A*** Body weight (top), heart weight (middle) and heart weight normalized to tibial length (bottom). ***B*** Systolic (top), diastolic (middle) and mean arterial (bottom) blood pressures in WT and SGK1 CA mice fed HFD. ***C*** Transthoracic echocardiography measured cardiac structural/functional parameters in obese WT and SGK1 CA mice. LVEDD, left ventricular end diastolic diameter; FS, fractional shortening.

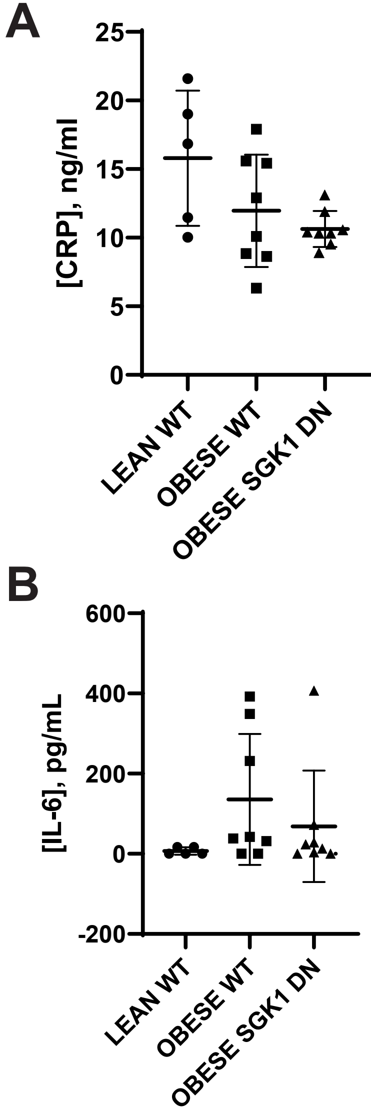

A
25
20
15
10
5
0
[CRP], ng/ml
LEAN WT
OBESE WT
OBESE SGK1 DN
B
600
400
200
0
-200
[IL-6], pg/mL
LEAN WT
OBESE WT
OBESE SGK1 DN

Supplemental Figure 7: Cardiac specificity of SGK1 DN transgene. ***A*** Western blots of hemagglutinin (HA) epitope tag present in only the atrial and ventricles of SGK1 DN mice, but not in non-cardiac tissues or in WT mice. ***B*** Example DNA blot demonstrating presence of SGK1 transgene in SGK1 DN, but not WT, mice.

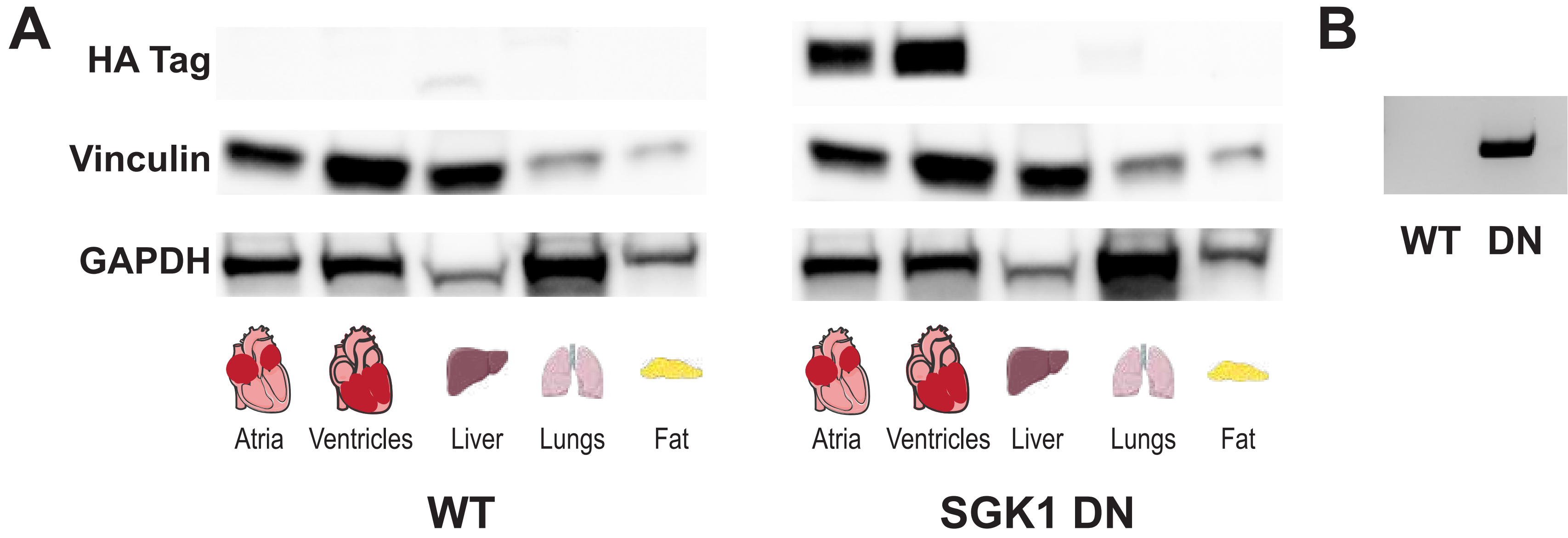
A
HA Tag
Vinculin
GAPDH
Atria
Ventricles
Liver
Lungs
Fat
WT
Atria
Ventricles
Liver
Lungs
Fat
SGK1 DN
B
WT
DN

Supplemental Table 1: Differentially expressed genes identified in RNA sequencing of lean and obese mouse atria.

Ensembl Gene ID	Associated Gene Name	Description	Chromosome Name	Gene Start (bp)	Gene End (bp)	Strand	Gene type	Base Mean	log2 Fold Change	lfcSE	Stat	P-value	Padj
ENSMUSG00000002289	ANGPTL4	angiopoietin-like 4 [Source:MGI Symbol;Acc:MGI:1888999]	17	33773750	33781575	-1	protein_coding	804.6233	5.02171722	0.249222	20.14958	2.71E-90	4.33E-86
ENSMUSG00000019577	PDK4	pyruvate dehydrogenase kinase, isoenzyme 4 [Source:MGI Symbol;Acc:MGI:1351481]	6	5483351	5496309	-1	protein_coding	27992.59	3.23206885	0.213354	15.14886	7.71E-52	6.14E-48
ENSMUSG00000023067	CDKN1A	cyclin-dependent kinase inhibitor 1A (P21) [Source:MGI Symbol;Acc:MGI:104556]	17	29090979	29100722	1	protein_coding	1501.125	2.32500937	0.164271	14.15348	1.78E-45	9.44E-42
ENSMUSG00000031762	MT2	metallothionein 2 [Source:MGI Symbol;Acc:MGI:97172]	8	94172618	94173567	1	protein_coding	546.8068	2.75868892	0.207502	13.29474	2.48E-40	9.90E-37
ENSMUSG00000019082	SLC25A22	solute carrier family 25 (mitochondrial carrier, glutamate), member 22 [Source:MGI Symbol;Acc:MGI:1915517]	7	141429739	141437892	-1	protein_coding	1156.869	1.32357095	0.134466	9.843196	7.33E-23	2.34E-19
ENSMUSG00000030433	SBK2	SH3-binding domain kinase family, member 2 [Source:MGI Symbol;Acc:MGI:2685925]	7	4956134	4964390	-1	protein_coding	3918.516	-1.0795827	0.118736	-9.09231	9.70E-20	2.58E-16
ENSMUSG00000038393	TXNIP	thioredoxin interacting protein [Source:MGI Symbol;Acc:MGI:1889549]	3	96557957	96561883	1	protein_coding	12047.44	1.65403344	0.185122	8.934848	4.08E-19	9.28E-16
ENSMUSG00000027313	CHAC1	ChaC, cation transport regulator 1 [Source:MGI Symbol;Acc:MGI:1916315]	2	119351229	119354381	1	protein_coding	151.4858	2.58063991	0.301228	8.567071	1.06E-17	2.12E-14
ENSMUSG00000044006	CILP2	cartilage intermediate layer protein 2 [Source:MGI Symbol;Acc:MGI:1915959]	8	69880369	69887687	-1	protein_coding	3293.716	-1.1702195	0.140853	-8.30808	9.73E-17	1.72E-13
ENSMUSG00000021226	ACOT2	acyl-CoA thioesterase 2 [Source:MGI Symbol;Acc:MGI:2159605]	12	83987861	83993873	1	protein_coding	1491.671	1.62906926	0.208646	7.807827	5.82E-15	9.27E-12
ENSMUSG00000005667	MTHFD2	methylenetetrahydrofolate dehydrogenase (NAD+ dependent), methenyltetrahydrofolate cyclohydrolase [Source:MGI Symbol;Acc:MGI:1	6	83305691	83317606	-1	protein_coding	253.5138	1.73636588	0.223127	7.781965	7.14E-15	1.03E-11
ENSMUSG00000003123	LIPE	lipase, hormone sensitive [Source:MGI Symbol;Acc:MGI:96790]	7	25379527	25398710	-1	protein_coding	1617.026	1.56053445	0.210039	7.429742	1.09E-13	1.45E-10
ENSMUSG00000073418	C4B	complement component 4B (Chido blood group) [Source:MGI Symbol;Acc:MGI:88228]	17	34728380	34743882	-1	protein_coding	2886.994	1.13757278	0.153815	7.3957	1.41E-13	1.69E-10
ENSMUSG00000020427	IGFBP3	insulin-like growth factor binding protein 3 [Source:MGI Symbol;Acc:MGI:96438]	11	7206086	7213923	-1	protein_coding	951.1028	-1.1877668	0.160751	-7.38885	1.48E-13	1.69E-10
ENSMUSG00000086320	GM12840	predicted gene 12840 [Source:MGI Symbol;Acc:MGI:3650917]	4	117700188	117700923	1	lincRNA	139.2374	2.33477158	0.319252	7.313257	2.61E-13	2.77E-10
ENSMUSG00000019122	CCL9	chemokine (C-C motif) ligand 9 [Source:MGI Symbol;Acc:MGI:104533]	11	83572919	83578636	-1	protein_coding	382.1655	1.55549051	0.215089	7.231848	4.76E-13	4.75E-10
ENSMUSG00000018822	SFRP5	secreted frizzled-related sequence protein 5 [Source:MGI Symbol;Acc:MGI:1860298]	19	42197971	42202252	-1	protein_coding	1741.266	-1.3371518	0.185194	-7.22027	5.19E-13	4.84E-10
ENSMUSG00000040283	BTNL9	butyrophilin-like 9 [Source:MGI Symbol;Acc:MGI:2442439]	11	49165585	49187159	-1	protein_coding	505.2259	1.43547452	0.199004	7.213298	5.46E-13	4.84E-10
ENSMUSG00000031167	RBM3	RNA binding motif protein 3 [Source:MGI Symbol;Acc:MGI:1099460]	X	8138975	8145880	-1	protein_coding	1262.189	-0.9708624	0.136562	-7.10933	1.17E-12	9.78E-10
ENSMUSG00000059824	DBP	D site albumin promoter binding protein [Source:MGI Symbol;Acc:MGI:94866]	7	45705088	45710155	1	protein_coding	350.4828	-1.4408056	0.205008	-7.02803	2.09E-12	1.67E-09
ENSMUSG00000031765	MT1	metallothionein 1 [Source:MGI Symbol;Acc:MGI:97171]	8	94179089	94180325	1	protein_coding	2165.076	1.44821882	0.206359	7.017962	2.25E-12	1.67E-09
ENSMUSG00000018927	CCL6	chemokine (C-C motif) ligand 6 [Source:MGI Symbol;Acc:MGI:98263]	11	83587882	83593087	-1	protein_coding	792.1702	1.32931207	0.189504	7.01468	2.30E-12	1.67E-09
ENSMUSG00000037035	INHBB	inhibin beta-B [Source:MGI Symbol;Acc:MGI:96571]	1	119415465	119422248	-1	protein_coding	147.962	1.8977025	0.28205	6.728238	1.72E-11	1.19E-08
ENSMUSG00000085272	SBK3	SH3 domain binding kinase family, member 3 [Source:MGI Symbol;Acc:MGI:2685924]	7	4965260	4971168	-1	protein_coding	11919.67	-0.9808495	0.146735	-6.68449	2.32E-11	1.54E-08
ENSMUSG00000029752	ASNS	asparagine synthetase [Source:MGI Symbol;Acc:MGI:1350929]	6	7675169	7693254	-1	protein_coding	169.3972	1.78499626	0.267939	6.661943	2.70E-11	1.72E-08
ENSMUSG00000043079	SYNPO	synaptopodin [Source:MGI Symbol;Acc:MGI:1099446]	18	60593973	60660142	-1	protein_coding	2460.897	0.92578793	0.139415	6.640511	3.13E-11	1.92E-08
ENSMUSG00000048416	MLF1	myeloid leukemia factor 1 [Source:MGI Symbol;Acc:MGI:1341819]	3	67374097	67400003	1	protein_coding	1635.094	-0.9232833	0.140839	-6.55559	5.54E-11	3.27E-08
ENSMUSG00000024866	ACY3	aspartoacylase (aminoacylase) 3 [Source:MGI Symbol;Acc:MGI:1918920]	19	3986661	3990007	1	protein_coding	636.9942	-1.0066099	0.156853	-6.41752	1.39E-10	7.89E-08
ENSMUSG00000057123	GJA5	gap junction protein, alpha 5 [Source:MGI Symbol;Acc:MGI:95716]	3	97032416	97053634	1	protein_coding	2052.627	-1.1276711	0.178451	-6.3192	2.63E-10	1.45E-07
ENSMUSG00000048489	8430408G22RIK	RIKEN cDNA 8430408G22 gene [Source:MGI Symbol;Acc:MGI:1918730]	6	116650684	116652847	1	protein_coding	413.2504	1.48492776	0.237586	6.250051	4.10E-10	2.18E-07
ENSMUSG00000020653	KLF11	Kruppel-like factor 11 [Source:MGI Symbol;Acc:MGI:2653368]	12	24651371	24662774	1	protein_coding	292.6886	1.45133579	0.233452	6.216839	5.07E-10	2.61E-07
ENSMUSG00000037411	SERPINE1	serine (or cysteine) peptidase inhibitor, clade E, member 1 [Source:MGI Symbol;Acc:MGI:97608]	5	137061504	137072272	-1	protein_coding	328.6786	1.72466699	0.279354	6.173764	6.67E-10	3.32E-07
ENSMUSG00000024222	FKBP5	FK506 binding protein 5 [Source:MGI Symbol;Acc:MGI:104670]	17	28399095	28517524	-1	protein_coding	741.1681	1.56080977	0.253156	6.165411	7.03E-10	3.40E-07
ENSMUSG00000036913	TRIM67	tripartite motif-containing 67 [Source:MGI Symbol;Acc:MGI:3045323]	8	124793019	124834713	1	protein_coding	478.211	1.35170383	0.220414	6.132578	8.65E-10	4.05E-07
ENSMUSG00000038539	ATF5	activating transcription factor 5 [Source:MGI Symbol;Acc:MGI:2141857]	7	44812257	44816658	-1	protein_coding	2011.542	0.98721024	0.161774	6.102422	1.04E-09	4.76E-07
ENSMUSG00000029657	HSPH1	heat shock 105kDa/110kDa protein 1 [Source:MGI Symbol;Acc:MGI:105053]	5	149614287	149636376	-1	protein_coding	1804.959	1.14808202	0.189296	6.06501	1.32E-09	5.84E-07
ENSMUSG00000030787	LYVE1	lymphatic vessel endothelial hyaluronan receptor 1 [Source:MGI Symbol;Acc:MGI:2136348]	7	110850607	110862953	-1	protein_coding	1257.974	0.88382546	0.146254	6.043067	1.51E-09	6.51E-07
ENSMUSG00000075602	LY6A	lymphocyte antigen 6 complex, locus A [Source:MGI Symbol;Acc:MGI:107527]	15	74994877	74998031	-1	protein_coding	1491.696	1.02358	0.170534	6.002205	1.95E-09	8.17E-07
ENSMUSG00000022270	FAM134B	family with sequence similarity 134, member B [Source:MGI Symbol;Acc:MGI:1913520]	15	25843264	25973687	1	protein_coding	2482.821	0.7747132	0.131075	5.910439	3.41E-09	1.39E-06
ENSMUSG00000032942	UCP3	uncoupling protein 3 (mitochondrial, proton carrier) [Source:MGI Symbol;Acc:MGI:1099787]	7	100472990	100486432	1	protein_coding	846.2819	2.88216129	0.49048	5.876203	4.20E-09	1.67E-06
ENSMUSG00000085779	ATCAYOS	ataxia, cerebellar, Cayman type homolog (human), opposite strand [Source:MGI Symbol;Acc:MGI:1916928]	10	81194609	81210877	1	antisense	549.3462	-1.3201457	0.226034	-5.84049	5.20E-09	2.02E-06
ENSMUSG00000035673	SBNO2	strawberry notch homolog 2 (Drosophila) [Source:MGI Symbol;Acc:MGI:2448490]	10	80057416	80102698	-1	protein_coding	445.0976	1.08280216	0.186907	5.793275	6.90E-09	2.62E-06
ENSMUSG00000035357	PDZRN3	PDZ domain containing RING finger 3 [Source:MGI Symbol;Acc:MGI:1933157]	6	101149609	101377897	-1	protein_coding	1475.169	0.85582692	0.150064	5.703081	1.18E-08	4.36E-06
ENSMUSG00000028862	MAP3K6	mitogen-activated protein kinase kinase kinase 6 [Source:MGI Symbol;Acc:MGI:1855691]	4	133240818	133252929	1	protein_coding	349.8396	1.13743036	0.201146	5.654759	1.56E-08	5.65E-06
ENSMUSG00000026822	LCN2	lipocalin 2 [Source:MGI Symbol;Acc:MGI:96757]	2	32384633	32388252	-1	protein_coding	96.89466	2.56793452	0.457779	5.60955	2.03E-08	7.19E-06
ENSMUSG00000057580	GM10012	predicted pseudogene 10012 [Source:MGI Symbol;Acc:MGI:3642454]	17	25901856	25902227	1	transcribed_processed_pse	590.7477	1.14155393	0.203894	5.598753	2.16E-08	7.48E-06
ENSMUSG00000030786	ITGAM	integrin alpha M [Source:MGI Symbol;Acc:MGI:96607]	7	128062640	128128160	1	protein_coding	205.4576	1.19122015	0.213635	5.575949	2.46E-08	8.18E-06
ENSMUSG00000059325	HOPX	HOP homeobox [Source:MGI Symbol;Acc:MGI:1916782]	5	77086988	77115121	-1	protein_coding	2167.565	-0.8980733	0.161062	-5.57594	2.46E-08	8.18E-06
ENSMUSG00000021922	ITIH4	inter alpha-trypsin inhibitor, heavy chain 4 [Source:MGI Symbol;Acc:MGI:109536]	14	30886476	30901981	1	protein_coding	214.7741	1.84735051	0.333616	5.537359	3.07E-08	9.99E-06
ENSMUSG00000047592	NXPE5	neurexophilin and PC-esterase domain family, member 5 [Source:MGI Symbol;Acc:MGI:3584036]	5	138225898	138253363	1	protein_coding	42.48076	2.66719502	0.483181	5.520069	3.39E-08	1.08E-05
ENSMUSG00000040010	SLC7A5	solute carrier family 7 (cationic amino acid transporter, y+ system), member 5 [Source:MGI Symbol;Acc:MGI:1298205]	8	121881150	121907694	-1	protein_coding	268.1376	1.08644695	0.197803	5.492584	3.96E-08	1.24E-05
ENSMUSG00000038642	CTSS	cathepsin S [Source:MGI Symbol;Acc:MGI:107341]	3	95526786	95556403	1	protein_coding	689.3358	0.88259362	0.161277	5.472533	4.44E-08	1.36E-05
ENSMUSG00000008845	CD163	CD163 antigen [Source:MGI Symbol;Acc:MGI:2135946]	6	124304651	124330527	1	protein_coding	784.0656	0.97299103	0.177923	5.468612	4.54E-08	1.36E-05
ENSMUSG00000020363	GFPT2	glutamine fructose-6-phosphate transaminase 2 [Source:MGI Symbol;Acc:MGI:1338883]	11	49794178	49838613	1	protein_coding	839.9121	1.00957679	0.185438	5.44429	5.20E-08	1.54E-05
ENSMUSG00000034449	DHRS11	dehydrogenase/reductase (SDR family) member 11 [Source:MGI Symbol;Acc:MGI:2652816]	11	84820856	84828994	-1	protein_coding	1186.332	-0.7703433	0.141618	-5.43957	5.34E-08	1.55E-05
ENSMUSG00000030616	SYTL2	synaptotagmin-like 2 [Source:MGI Symbol;Acc:MGI:1933366]	7	90348699	90410439	1	protein_coding	623.6722	-0.8967012	0.166252	-5.39364	6.90E-08	1.97E-05
ENSMUSG00000028996	RBP7	retinol binding protein 7, cellular [Source:MGI Symbol;Acc:MGI:1890409]	4	149449687	149454978	-1	protein_coding	116.5335	1.50985234	0.282723	5.34039	9.27E-08	2.59E-05
ENSMUSG00000023959	CLIC5	chloride intracellular channel 5 [Source:MGI Symbol;Acc:MGI:1917912]	17	44188572	44280168	1	protein_coding	2866.265	0.82236615	0.154198	5.333186	9.65E-08	2.65E-05
ENSMUSG00000079419	MS4A6C	membrane-spanning 4-domains, subfamily A, member 6C [Source:MGI Symbol;Acc:MGI:2385644]	19	11469366	11482192	1	protein_coding	138.0316	1.34354236	0.253988	5.289789	1.22E-07	3.27E-05
ENSMUSG00000031980	AGT	angiotensinogen (serpin peptidase inhibitor, clade A, member 8) [Source:MGI Symbol;Acc:MGI:87963]	8	124556534	124569706	-1	protein_coding	549.2882	1.24684132	0.235742	5.289012	1.23E-07	3.27E-05
ENSMUSG00000097815	GM26809	predicted gene, 26809 [Source:MGI Symbol;Acc:MGI:5477303]	6	18844220	18847360	1	lincRNA	216.1503	1.27719108	0.242011	5.277414	1.31E-07	3.42E-05
ENSMUSG00000037263	1700055N04RIK	RIKEN cDNA 1700055N04 gene [Source:MGI Symbol;Acc:MGI:1920708]	19	3958645	3972103	1	protein_coding	174.2808	-1.942411	0.369343	-5.2591	1.45E-07	3.68E-05
ENSMUSG00000047085	LRRC4B	leucine rich repeat containing 4B [Source:MGI Symbol;Acc:MGI:3027390]	7	44429018	44463351	1	protein_coding	284.3423	-1.1228489	0.213592	-5.25698	1.46E-07	3.68E-05
ENSMUSG00000033044	DHRS7C	dehydrogenase/reductase (SDR family) member 7C [Source:MGI Symbol;Acc:MGI:1915710]	11	67798187	67816005	1	protein_coding	2247.637	-0.7672727	0.145993	-5.25553	1.48E-07	3.68E-05
ENSMUSG00000018486	WNT9B	wingless-type MMTV integration site family, member 9B [Source:MGI Symbol;Acc:MGI:1197020]	11	103727364	103749821	-1	protein_coding	42.64581	2.43706825	0.464275	5.249189	1.53E-07	3.74E-05
ENSMUSG00000096768	ERDR1	erythroid differentiation regulator 1 [Source:MGI Symbol;Acc:MGI:2384747]	Y	90784738	90816464	1	protein_coding	550.611	1.19552507	0.227864	5.246657	1.55E-07	3.74E-05
ENSMUSG00000024526	CIDEA	cell death-inducing DNA fragmentation factor, alpha subunit-like effector A [Source:MGI Symbol;Acc:MGI:1270845]	18	67343564	67367794	1	protein_coding	256.6024	1.22395614	0.234023	5.230065	1.69E-07	4.03E-05
ENSMUSG00000011305	PLIN5	perilipin 5 [Source:MGI Symbol;Acc:MGI:1914218]	17	56111601	56117596	-1	protein_coding	743.3789	1.03963494	0.198885	5.227311	1.72E-07	4.03E-05
ENSMUSG00000044338	APLNR	apelin receptor [Source:MGI Symbol;Acc:MGI:1346086]	2	85136225	85139923	1	protein_coding	355.0376	-1.089743	0.208875	-5.21721	1.82E-07	4.14E-05
ENSMUSG00000024871	DOC2G	double C2, gamma [Source:MGI Symbol;Acc:MGI:1926250]	19	4003321	4007005	1	protein_coding	6095.003	-0.8940465	0.171367	-5.21714	1.82E-07	4.14E-05
ENSMUSG00000013846	ST3GAL1	ST3 beta-galactoside alpha-2,3-sialyltransferase 1 [Source:MGI Symbol;Acc:MGI:98304]	15	67102875	67113992	-1	protein_coding	1063.427	0.76913588	0.148007	5.196633	2.03E-07	4.56E-05
ENSMUSG00000034533	SCN10A	sodium channel, voltage-gated, type X, alpha [Source:MGI Symbol;Acc:MGI:108029]	9	119608456	119719032	-1	protein_coding	114.8619	-1.5999003	0.309424	-5.17058	2.33E-07	5.17E-05
ENSMUSG00000002944	CD36	CD36 antigen [Source:MGI Symbol;Acc:MGI:107899]	5	17781690	17888959	-1	protein_coding	19606.74	0.73249579	0.143911	5.089913	3.58E-07	7.73E-05
ENSMUSG00000025153	FASN	fatty acid synthase [Source:MGI Symbol;Acc:MGI:95485]	11	120805940	120824178	-1	protein_coding	1271.421	-1.0842316	0.213034	-5.08948	3.59E-07	7.73E-05

ENSMUSG00000049556	LINGO1	leucine rich repeat and Ig domain containing 1 [Source:MGI Symbol;Acc:MGI:1915522]	9	56618475	56685253	-1	protein_coding	320.2452	-0.9608345	0.189527	-5.06965	3.99E-07	8.47E-05
ENSMUSG00000033581	IGF2BP2	insulin-like growth factor 2 mRNA binding protein 2 [Source:MGI Symbol;Acc:MGI:1890358]	16	22059009	22163299	-1	protein_coding	253.4544	1.18747466	0.234362	5.066849	4.04E-07	8.48E-05
ENSMUSG00000053898	ECH1	enoyl coenzyme A hydratase 1, peroxisomal [Source:MGI Symbol;Acc:MGI:1858208]	7	28825217	28832247	1	protein_coding	11785.59	0.6775922	0.133827	5.063204	4.12E-07	8.53E-05
ENSMUSG00000037465	KLF10	Kruppel-like factor 10 [Source:MGI Symbol;Acc:MGI:1101353]	15	38294413	38300707	-1	protein_coding	531.6971	0.90071117	0.178015	5.059739	4.20E-07	8.58E-05
ENSMUSG00000061718	PPP1R1B	protein phosphatase 1, regulatory (inhibitor) subunit 1B [Source:MGI Symbol;Acc:MGI:94860]	11	98348404	98357796	1	protein_coding	809.3729	-0.7968122	0.158518	-5.02665	4.99E-07	0.000101
ENSMUSG00000062257	OPCML	opioid binding protein/cell adhesion molecule-like [Source:MGI Symbol;Acc:MGI:97397]	9	27790775	28925410	1	protein_coding	313.8704	-1.2562427	0.251515	-4.99471	5.89E-07	0.000117
ENSMUSG00000075289	CARNS1	carnosine synthase 1 [Source:MGI Symbol;Acc:MGI:2147595]	19	4164324	4175479	-1	protein_coding	1149.152	-0.7642455	0.153379	-4.98273	6.27E-07	0.000123
ENSMUSG00000053166	CDH22	cadherin 22 [Source:MGI Symbol;Acc:MGI:1341843]	2	165111507	165234853	-1	protein_coding	286.5529	1.15276198	0.231686	4.975546	6.51E-07	0.000126
ENSMUSG00000024900	CPT1A	carnitine palmitoyltransferase 1a, liver [Source:MGI Symbol;Acc:MGI:1098296]	19	3323301	3385732	1	protein_coding	1265.495	0.88768259	0.17844	4.974689	6.54E-07	0.000126
ENSMUSG00000054203	IFI205	interferon activated gene 205 [Source:MGI Symbol;Acc:MGI:101847]	1	174011998	174031810	-1	protein_coding	367.9498	0.95565448	0.193053	4.950221	7.41E-07	0.00014
ENSMUSG00000051262	CML3	camello-like 3 [Source:MGI Symbol;Acc:MGI:2136449]	6	85760649	85765744	-1	protein_coding	353.5159	-0.9451813	0.191011	-4.94831	7.49E-07	0.00014
ENSMUSG00000073731	AI507597	expressed sequence AI507597 [Source:MGI Symbol;Acc:MGI:2140323]	4	141614026	141615604	1	antisense	24.63397	3.49768877	0.708458	4.937042	7.93E-07	0.000147
ENSMUSG00000019951	UHRF1BP1L	UHRF1 (ICBP90) binding protein 1-like [Source:MGI Symbol;Acc:MGI:2442888]	10	89744991	89819869	1	protein_coding	441.0463	0.81859456	0.166348	4.920965	8.61E-07	0.000158
ENSMUSG00000024066	XDH	xanthine dehydrogenase [Source:MGI Symbol;Acc:MGI:98973]	17	73883908	73950182	-1	protein_coding	1709.475	0.82123619	0.167648	4.898578	9.65E-07	0.000174
ENSMUSG00000023092	FHL1	four and a half LIM domains 1 [Source:MGI Symbol;Acc:MGI:1298387]	X	56731787	56793346	1	protein_coding	5045.699	0.9795626	0.200008	4.89761	9.70E-07	0.000174
ENSMUSG00000026064	PTP4A1	protein tyrosine phosphatase 4a1 [Source:MGI Symbol;Acc:MGI:1277096]	1	30940302	30949770	-1	protein_coding	2089.249	0.60162635	0.12337	4.876616	1.08E-06	0.000191
ENSMUSG00000028494	PLIN2	perilipin 2 [Source:MGI Symbol;Acc:MGI:87920]	4	86648386	86670060	-1	protein_coding	718.9873	0.87322188	0.179228	4.872127	1.10E-06	0.000192
ENSMUSG00000030790	ADM	adrenomedullin [Source:MGI Symbol;Acc:MGI:108058]	7	110627661	110629820	1	protein_coding	805.4614	-0.8430181	0.173051	-4.87149	1.11E-06	0.000192
ENSMUSG00000042834	NREP	neuronal regeneration related protein [Source:MGI Symbol;Acc:MGI:99444]	18	33437019	33464029	-1	protein_coding	392.8068	-0.9159925	0.188158	-4.86821	1.13E-06	0.000193
ENSMUSG00000056501	CEBPB	CCAAT/enhancer binding protein (C/EBP), beta [Source:MGI Symbol;Acc:MGI:88373]	2	167688915	167690418	1	protein_coding	647.4718	1.08955498	0.225547	4.830717	1.36E-06	0.000231
ENSMUSG00000037438	GM9763	predicted pseudogene 9763 [Source:MGI Symbol;Acc:MGI:3641869]	15	99898287	99898556	-1	processed_pseudogene	13.29016	-7.0983155	1.472594	-4.82028	1.43E-06	0.000241
ENSMUSG00000003228	GRK5	G protein-coupled receptor kinase 5 [Source:MGI Symbol;Acc:MGI:109161]	19	60889749	61092553	1	protein_coding	932.3509	0.67525001	0.140467	4.807164	1.53E-06	0.000254
ENSMUSG00000014813	STC1	stanniocalcin 1 [Source:MGI Symbol;Acc:MGI:109131]	14	69029289	69041401	1	protein_coding	160.5197	1.60705254	0.334561	4.803466	1.56E-06	0.000256
ENSMUSG00000017778	COX7C	cytochrome c oxidase subunit VIIc [Source:MGI Symbol;Acc:MGI:103226]	13	86044816	86046904	-1	protein_coding	732.9165	-1.0330917	0.215733	-4.78874	1.68E-06	0.000273
ENSMUSG00000043085	TMEM82	transmembrane protein 82 [Source:MGI Symbol;Acc:MGI:2384869]	4	141613376	141618633	-1	protein_coding	111.1707	1.82165007	0.380828	4.78339	1.72E-06	0.000278
ENSMUSG00000037071	SCD1	stearoyl-Coenzyme A desaturase 1 [Source:MGI Symbol;Acc:MGI:98239]	19	44394451	44407709	-1	protein_coding	1783.703	-1.0663774	0.22309	-4.78002	1.75E-06	0.000278
ENSMUSG00000020717	PECAM1	platelet/endothelial cell adhesion molecule 1 [Source:MGI Symbol;Acc:MGI:97537]	11	106654217	106750628	-1	protein_coding	4953.032	0.56912352	0.119093	4.778825	1.76E-06	0.000278
ENSMUSG00000015143	ACTN1	actinin, alpha 1 [Source:MGI Symbol;Acc:MGI:2137706]	12	80167542	80260371	-1	protein_coding	749.6408	0.75538331	0.158296	4.771953	1.82E-06	0.000284
ENSMUSG00000082035	RPL17-PS8	ribosomal protein L17, pseudogene 8 [Source:MGI Symbol;Acc:MGI:3802129]	X	96484859	96485479	1	processed_pseudogene	59.74423	-1.9773695	0.414485	-4.77067	1.84E-06	0.000284
ENSMUSG00000090084	SRPX	sushi-repeat-containing protein [Source:MGI Symbol;Acc:MGI:1858306]	X	10037977	10117709	-1	protein_coding	627.3845	1.02926234	0.217101	4.740945	2.13E-06	0.000326
ENSMUSG00000019124	SCRN1	secernin 1 [Source:MGI Symbol;Acc:MGI:1917188]	6	54505890	54566489	-1	protein_coding	1091.002	-0.6701337	0.141417	-4.7387	2.15E-06	0.000327
ENSMUSG00000033174	MGLL	monoglyceride lipase [Source:MGI Symbol;Acc:MGI:1346042]	6	88724412	88828360	1	protein_coding	2374.848	0.5971945	0.126115	4.735319	2.19E-06	0.000329
ENSMUSG00000002831	PLIN4	perilipin 4 [Source:MGI Symbol;Acc:MGI:1929709]	17	56100591	56109803	-1	protein_coding	4518.68	0.74129033	0.156682	4.731167	2.23E-06	0.000333
ENSMUSG00000021091	SERPINA3N	serine (or cysteine) peptidase inhibitor, clade A, member 3N [Source:MGI Symbol;Acc:MGI:105045]	12	104406729	104414329	1	protein_coding	1546.963	2.63750529	0.558792	4.720012	2.36E-06	0.000348
ENSMUSG00000009185	CCL8	chemokine (C-C motif) ligand 8 [Source:MGI Symbol;Acc:MGI:101878]	11	82115185	82116799	1	protein_coding	108.1816	1.82220959	0.386435	4.71544	2.41E-06	0.000353
ENSMUSG00000035561	ALDH1B1	aldehyde dehydrogenase 1 family, member B1 [Source:MGI Symbol;Acc:MGI:1919785]	4	45799022	45804604	1	protein_coding	2071.878	-0.6138178	0.130465	-4.70485	2.54E-06	0.000368
ENSMUSG00000102260	RP24-281O20.2		18	80362781	80365826	1	TEC	248.1601	-1.081892	0.230136	-4.7011	2.59E-06	0.000372
ENSMUSG00000001663	GSTT1	glutathione S-transferase, theta 1 [Source:MGI Symbol;Acc:MGI:107379]	10	75783813	75798584	-1	protein_coding	640.6119	-0.7715061	0.164552	-4.68852	2.75E-06	0.000392
ENSMUSG00000031790	MMP15	matrix metallopeptidase 15 [Source:MGI Symbol;Acc:MGI:109320]	8	95352268	95374293	1	protein_coding	2507.67	-0.7138442	0.152726	-4.67402	2.95E-06	0.000417
ENSMUSG00000043733	PTPN11	protein tyrosine phosphatase, non-receptor type 11 [Source:MGI Symbol;Acc:MGI:99511]	5	121130533	121191397	-1	protein_coding	4786.365	0.67789581	0.145271	4.666437	3.06E-06	0.000429
ENSMUSG00000071637	CEBPD	CCAAT/enhancer binding protein (C/EBP), delta [Source:MGI Symbol;Acc:MGI:103573]	16	15887286	15888466	1	pseudogene	437.8784	0.9561665	0.205092	4.662127	3.13E-06	0.000434
ENSMUSG00000073490	AI607873	expressed sequence AI607873 [Source:MGI Symbol;Acc:MGI:2138302]	1	173723430	173741809	-1	protein_coding	346.2847	0.90721907	0.195301	4.64523	3.40E-06	0.000467
ENSMUSG00000032487	PTGS2	prostaglandin-endoperoxide synthase 2 [Source:MGI Symbol;Acc:MGI:97798]	1	150100031	150108227	1	protein_coding	105.0805	1.54464051	0.335861	4.599051	4.24E-06	0.000578
ENSMUSG00000034652	CD300A	CD300A antigen [Source:MGI Symbol;Acc:MGI:2443411]	11	114890041	114904654	1	protein_coding	80.20095	1.73144144	0.37799	4.580658	4.64E-06	0.000621
ENSMUSG00000058624	GDA	guanine deaminase [Source:MGI Symbol;Acc:MGI:95678]	19	21391307	21473445	-1	protein_coding	484.6148	0.84517382	0.184511	4.580607	4.64E-06	0.000621
ENSMUSG00000086922	GM13835	predicted gene 13835 [Source:MGI Symbol;Acc:MGI:3650921]	6	31141979	31142663	-1	processed_pseudogene	341.5249	-0.9984169	0.218318	-4.57322	4.80E-06	0.000638
ENSMUSG00000024905	MTL5	metallothionein-like 5, testis-specific (tesmin) [Source:MGI Symbol;Acc:MGI:1340029]	19	3388857	3407823	1	protein_coding	103.3021	1.70031298	0.372247	4.567702	4.93E-06	0.00065
ENSMUSG00000030096	SLC6A6	solute carrier family 6 (neurotransmitter transporter, taurine), member 6 [Source:MGI Symbol;Acc:MGI:98488]	6	91684067	91759063	1	protein_coding	1383.119	0.72147595	0.159598	4.520586	6.17E-06	0.000806
ENSMUSG00000019945	1700040L02RIK	RIKEN cDNA 1700040L02 gene [Source:MGI Symbol;Acc:MGI:1920537]	10	68430953	68541896	-1	protein_coding	238.3908	-0.9510727	0.21061	-4.51581	6.31E-06	0.000817
ENSMUSG00000054966	LMNTD1	lamin tail domain containing 1 [Source:MGI Symbol;Acc:MGI:1921321]	6	145365134	145614319	-1	protein_coding	1626.9	-0.6604807	0.146555	-4.5067	6.58E-06	0.000846
ENSMUSG00000042099	KANK3	KN motif and ankyrin repeat domains 3 [Source:MGI Symbol;Acc:MGI:1098615]	17	33810520	33822918	1	protein_coding	895.8017	0.66090852	0.146896	4.499164	6.82E-06	0.00087
ENSMUSG00000024529	LOX	lysyl oxidase [Source:MGI Symbol;Acc:MGI:96817]	18	52516069	52529867	-1	protein_coding	185.0514	0.98111324	0.219371	4.472385	7.74E-06	0.000979
ENSMUSG00000038583	PLN	phospholamban [Source:MGI Symbol;Acc:MGI:97622]	10	53337686	53345999	1	protein_coding	34390.74	-0.6888665	0.154248	-4.46597	7.97E-06	0.000995
ENSMUSG00000029333	RASGEF1B	RasGEF domain family, member 1B [Source:MGI Symbol;Acc:MGI:2443755]	5	99217426	99252927	-1	protein_coding	331.4013	0.93069057	0.208473	4.464312	8.03E-06	0.000995
ENSMUSG00000027322	SIGLEC1	sialic acid binding Ig-like lectin 1, sialoadhesin [Source:MGI Symbol;Acc:MGI:99668]	2	131069220	131086765	-1	protein_coding	139.4066	1.22601682	0.274654	4.463864	8.05E-06	0.000995
ENSMUSG00000052085	DOCK8	dedicator of cytokinesis 8 [Source:MGI Symbol;Acc:MGI:1921396]	19	24999529	25202432	1	protein_coding	276.2986	0.89387786	0.200336	4.461887	8.12E-06	0.000996
ENSMUSG00000026687	ALDH9A1	aldehyde dehydrogenase 9, subfamily A1 [Source:MGI Symbol;Acc:MGI:1861622]	1	167349991	167368532	1	protein_coding	687.7837	0.80666359	0.181161	4.452748	8.48E-06	0.001032
ENSMUSG00000020262	ADARB1	adenosine deaminase, RNA-specific, B1 [Source:MGI Symbol;Acc:MGI:891999]	10	77290726	77418270	-1	protein_coding	710.8496	0.77980201	0.176037	4.429753	9.43E-06	0.001139
ENSMUSG00000053398	PHGDH	3-phosphoglycerate dehydrogenase [Source:MGI Symbol;Acc:MGI:1355330]	3	98313170	98339990	-1	protein_coding	111.9449	1.46821854	0.333122	4.40745	1.05E-05	0.001254
ENSMUSG00000062515	FABP4	fatty acid binding protein 4, adipocyte [Source:MGI Symbol;Acc:MGI:88038]	3	10204088	10208576	-1	protein_coding	5252.225	0.82984308	0.188458	4.403331	1.07E-05	0.001268
ENSMUSG00000000948	SNRPN	small nuclear ribonucleoprotein N [Source:MGI Symbol;Acc:MGI:98347]	7	59974149	60005064	-1	protein_coding	26.75657	-3.4283116	0.779928	-4.39567	1.10E-05	0.001296
ENSMUSG00000010066	CACNA2D2	calcium channel, voltage-dependent, alpha 2/delta subunit 2 [Source:MGI Symbol;Acc:MGI:1929813]	9	107399612	107529343	1	protein_coding	1411.696	-0.6887185	0.156691	-4.3954	1.11E-05	0.001296
ENSMUSG00000002910	ARRDC2	arrestin domain containing 2 [Source:MGI Symbol;Acc:MGI:1918057]	8	70835155	70839720	-1	protein_coding	331.0077	1.30647419	0.298076	4.383025	1.17E-05	0.001362
ENSMUSG00000039883	LRRC17	leucine rich repeat containing 17 [Source:MGI Symbol;Acc:MGI:1921761]	5	21543527	21575900	1	protein_coding	155.0879	-1.1221649	0.256852	-4.36892	1.25E-05	0.001442
ENSMUSG00000028223	DECR1	2,4-dienoyl CoA reductase 1, mitochondrial [Source:MGI Symbol;Acc:MGI:1914710]	4	15917240	15945507	-1	protein_coding	3699.042	0.57346045	0.131756	4.35243	1.35E-05	0.001544
ENSMUSG00000085590	GM13789	predicted gene 13789 [Source:MGI Symbol;Acc:MGI:3723492]	5	114363415	114365357	1	processed_transcript	63.37388	-2.2112378	0.509003	-4.34425	1.40E-05	0.001591
ENSMUSG00000031137	FGF13	fibroblast growth factor 13 [Source:MGI Symbol;Acc:MGI:109178]	X	59062145	59568071	-1	protein_coding	527.9353	-0.7148279	0.164719	-4.33968	1.43E-05	0.001613
ENSMUSG00000044258	CTLA2A	cytotoxic T lymphocyte-associated protein 2 alpha [Source:MGI Symbol;Acc:MGI:88554]	13	60934155	60936625	-1	protein_coding	588.2498	0.72516941	0.167394	4.332117	1.48E-05	0.001658
ENSMUSG00000022868	AHSG	alpha-2-HS-glycoprotein [Source:MGI Symbol;Acc:MGI:107189]	16	22892042	22899438	1	protein_coding	682.6198	0.77723035	0.17982	4.322279	1.54E-05	0.001721
ENSMUSG00000003949	HLF	hepatic leukemia factor [Source:MGI Symbol;Acc:MGI:96108]	11	90336536	90390895	-1	protein_coding	572.4636	-0.8076022	0.187055	-4.31745	1.58E-05	0.001747
ENSMUSG00000040653	PPP1R14C	protein phosphatase 1, regulatory (inhibitor) subunit 14c [Source:MGI Symbol;Acc:MGI:1923392]	10	3366150	3464975	1	protein_coding	2040.283	-0.7861019	0.18223	-4.31378	1.60E-05	0.001764
ENSMUSG00000079534	GM5640	predicted gene 5640 [Source:MGI Symbol;Acc:MGI:3645126]	X	74639119	74645635	-1	protein_coding	85.28444	-1.4919081	0.346045	-4.31131	1.62E-05	0.001772
ENSMUSG00000031502	COL4A1	collagen, type IV, alpha 1 [Source:MGI Symbol;Acc:MGI:88454]	8	11198423	11312826	-1	protein_coding	7401.912	0.78079272	0.181385	4.304615	1.67E-05	0.001785
ENSMUSG00000026824	KCNJ3	potassium inwardly-rectifying channel, subfamily J, member 3 [Source:MGI Symbol;Acc:MGI:104742]	2	55435970	55598145	1	protein_coding	7841.166	-0.7047887	0.163736	-4.30443	1.67E-05	0.001785
ENSMUSG00000022389	TEF	thyrotroph embryonic factor [Source:MGI Symbol;Acc:MGI:98663]	15	81802421	81826861	1	protein_coding	1318.452	-0.7275047	0.169016	-4.30435	1.67E-05	0.001785
ENSMUSG00000019732	CALR3	calreticulin 3 [Source:MGI Symbol;Acc:MGI:1920566]	8	72424176	72443870	-1	protein_coding	360.0539	1.16588898	0.270906	4.303668	1.68E-05	0.001785
ENSMUSG00000031785	GPR56	G protein-coupled receptor 56 [Source:MGI Symbol;Acc:MGI:1340051]	8	94977109	95014208	1	protein_coding	683.4436	0.66614004	0.155283	4.289838	1.79E-05	0.001888

ENSMUSG00000044934	ZFP367	zinc finger protein 367 [Source:MGI Symbol;Acc:MGI:2442266]	13	64133022	64153202	-1	protein_coding	643.5515	-0.7269747	0.169704	-4.28378	1.84E-05	0.001927
ENSMUSG00000096210	H1F0	H1 histone family, member 0 [Source:MGI Symbol;Acc:MGI:95893]	15	79028212	79030498	1	protein_coding	1594.224	-0.5448177	0.127326	-4.27892	1.88E-05	0.001957
ENSMUSG00000051510	MAFG	v-maf musculoaponeurotic fibrosarcoma oncogene family, protein G (avian) [Source:MGI Symbol;Acc:MGI:96911]	11	120625117	120633600	-1	protein_coding	1191.395	0.56378626	0.131809	4.277299	1.89E-05	0.001958
ENSMUSG00000022994	ADCY6	adenylate cyclase 6 [Source:MGI Symbol;Acc:MGI:87917]	15	98589994	98607633	-1	protein_coding	4762.822	0.63023177	0.147488	4.273111	1.93E-05	0.001982
ENSMUSG00000034684	SEMA3F	sema domain, immunoglobulin domain (Ig), short basic domain, secreted, (semaphorin) 3F [Source:MGI Symbol;Acc:MGI:1096347]	9	107681500	107710475	-1	protein_coding	463.6199	0.87259516	0.204382	4.269436	1.96E-05	0.002002
ENSMUSG00000031438	RNF128	ring finger protein 128 [Source:MGI Symbol;Acc:MGI:1914139]	X	139563316	139673145	1	protein_coding	1028.276	0.63620188	0.149177	4.264759	2.00E-05	0.002032
ENSMUSG00000075296	ALDH3B2	aldehyde dehydrogenase 3 family, member B2 [Source:MGI Symbol;Acc:MGI:2147613]	19	3972328	3981645	1	protein_coding	45.37396	-2.0498603	0.48107	-4.26105	2.03E-05	0.002053
ENSMUSG00000038708	GOLGA4	golgi autoantigen, golgin subfamily a, 4 [Source:MGI Symbol;Acc:MGI:1859646]	9	118506318	118582522	1	protein_coding	5798.36	0.508577	0.119836	4.24394	2.20E-05	0.002202
ENSMUSG00000031880	RRAD	Ras-related associated with diabetes [Source:MGI Symbol;Acc:MGI:1930943]	8	104628066	104631321	-1	protein_coding	5184.386	0.63485366	0.149667	4.241769	2.22E-05	0.002202
ENSMUSG00000018796	ACSL1	acyl-CoA synthetase long-chain family member 1 [Source:MGI Symbol;Acc:MGI:102797]	8	46471041	46536051	1	protein_coding	9988.507	0.55846739	0.13168	4.241089	2.22E-05	0.002202
ENSMUSG00000079017	IFI27L2A	interferon, alpha-inducible protein 27 like 2A [Source:MGI Symbol;Acc:MGI:1924183]	12	103442167	103443680	-1	protein_coding	743.3463	0.9310099	0.220278	4.226527	2.37E-05	0.002335
ENSMUSG00000021133	4933426M11RIK	RIKEN cDNA 4933426M11 gene [Source:MGI Symbol;Acc:MGI:2444661]	12	80790532	80880832	1	protein_coding	2350.713	0.60713551	0.143961	4.217352	2.47E-05	0.002417
ENSMUSG00000052135	FOXO6	forkhead box O6 [Source:MGI Symbol;Acc:MGI:2676586]	4	120267079	120287349	-1	protein_coding	249.6928	-0.9602428	0.228324	-4.20561	2.60E-05	0.002531
ENSMUSG00000003934	EFNB3	ephrin B3 [Source:MGI Symbol;Acc:MGI:109196]	11	69554092	69560205	-1	protein_coding	551.0773	-0.8839343	0.210376	-4.20169	2.65E-05	0.002559
ENSMUSG00000031532	SARAF	store-operated calcium entry-associated regulatory factor [Source:MGI Symbol;Acc:MGI:1915137]	8	34154563	34170845	1	protein_coding	5022.844	0.47574902	0.114026	4.172277	3.02E-05	0.002896
ENSMUSG00000068745	MYBPHL	myosin binding protein H-like [Source:MGI Symbol;Acc:MGI:1916003]	3	108364911	108380056	1	protein_coding	9517.254	-0.6793494	0.163034	-4.16691	3.09E-05	0.002947
ENSMUSG00000024205	RPL36-PS2	ribosomal protein L36, pseudogene 2 [Source:MGI Symbol;Acc:MGI:3650081]	11	76601319	76601629	1	processed_pseudogene	133.687	-1.2505733	0.301561	-4.14699	3.37E-05	0.003196
ENSMUSG00000024087	CYP1B1	cytochrome P450, family 1, subfamily b, polypeptide 1 [Source:MGI Symbol;Acc:MGI:88590]	17	79706953	79715041	-1	protein_coding	636.8925	0.95275986	0.22998	4.142801	3.43E-05	0.003219
ENSMUSG00000029426	SCARB2	scavenger receptor class B, member 2 [Source:MGI Symbol;Acc:MGI:1196458]	5	92443873	92505608	-1	protein_coding	1804.685	0.66951914	0.161677	4.141081	3.46E-05	0.003219
ENSMUSG00000086607	4930511M06RIK	RIKEN cDNA 4930511M06 gene [Source:MGI Symbol;Acc:MGI:1922334]	18	57373455	57552397	-1	processed_transcript	27.70889	2.49574857	0.60276	4.140535	3.46E-05	0.003219
ENSMUSG00000040498	IGSF23	immunoglobulin superfamily, member 23 [Source:MGI Symbol;Acc:MGI:1917330]	7	19937305	19950743	-1	protein_coding	795.9056	0.67288127	0.16256	4.139282	3.48E-05	0.003219
ENSMUSG00000083899	GM12346	predicted gene 12346 [Source:MGI Symbol;Acc:MGI:3649810]	11	77293444	77295898	1	transcribed_processed_pse	401.0699	1.18222875	0.285692	4.138122	3.50E-05	0.003219
ENSMUSG00000012819	CDH23	cadherin 23 (otocadherin) [Source:MGI Symbol;Acc:MGI:1890219]	10	60302748	60696490	-1	protein_coding	75.66122	-1.4078012	0.34027	-4.1373	3.51E-05	0.003219
ENSMUSG00000006642	TCF23	transcription factor 23 [Source:MGI Symbol;Acc:MGI:1934960]	5	30968677	30977018	1	protein_coding	34.74371	2.25143782	0.546462	4.120026	3.79E-05	0.003451
ENSMUSG00000041773	ENC1	ectodermal-neural cortex 1 [Source:MGI Symbol;Acc:MGI:109610]	13	97241105	97253039	1	protein_coding	569.1644	0.79316947	0.192578	4.118691	3.81E-05	0.003451
ENSMUSG00000041329	ATP1B2	ATPase, Na+/K+ transporting, beta 2 polypeptide [Source:MGI Symbol;Acc:MGI:88109]	11	69599736	69605942	-1	protein_coding	617.127	-0.6711986	0.163253	-4.11139	3.93E-05	0.003542
ENSMUSG00000097417	GM26669	predicted gene, 26669 [Source:MGI Symbol;Acc:MGI:5477163]	12	80108034	80113000	1	lincRNA	83.62887	-1.3532621	0.329443	-4.10772	4.00E-05	0.003578
ENSMUSG00000030089	SLC41A3	solute carrier family 41, member 3 [Source:MGI Symbol;Acc:MGI:1918949]	6	90604725	90646412	1	protein_coding	2029.327	0.57880864	0.141063	4.1032	4.07E-05	0.003629
ENSMUSG00000076431	SOX4	SRY (sex determining region Y)-box 4 [Source:MGI Symbol;Acc:MGI:98366]	13	28948919	28953713	-1	protein_coding	567.3059	-0.6904086	0.168568	-4.09574	4.21E-05	0.003719
ENSMUSG00000020455	TRIM11	tripartite motif-containing 11 [Source:MGI Symbol;Acc:MGI:2137355]	11	58978093	58991458	1	protein_coding	845.8656	-0.6518907	0.159194	-4.09495	4.22E-05	0.003719
ENSMUSG00000001627	IFRD1	interferon-related developmental regulator 1 [Source:MGI Symbol;Acc:MGI:1316717]	12	40201567	40248504	-1	protein_coding	733.1164	0.76052643	0.185906	4.090924	4.30E-05	0.003763
ENSMUSG00000048120	ENTPD1	ectonucleoside triphosphate diphosphohydrolase 1 [Source:MGI Symbol;Acc:MGI:102805]	19	40612366	40741602	1	protein_coding	580.9444	0.63474606	0.155729	4.075967	4.58E-05	0.003991
ENSMUSG00000027332	IVD	isovaleryl coenzyme A dehydrogenase [Source:MGI Symbol;Acc:MGI:1929242]	2	118861954	118882909	1	protein_coding	5397.165	-0.4718904	0.115942	-4.07004	4.70E-05	0.004072
ENSMUSG00000030483	CYP2B10	cytochrome P450, family 2, subfamily b, polypeptide 10 [Source:MGI Symbol;Acc:MGI:88598]	7	25897620	25926624	1	protein_coding	102.3854	1.84509326	0.453611	4.067566	4.75E-05	0.004093
ENSMUSG00000020486	4-Sep	septin 4 [Source:MGI Symbol;Acc:MGI:1270156]	11	87568903	87590539	1	protein_coding	722.2211	-0.6826346	0.168277	-4.05662	4.98E-05	0.004234
ENSMUSG00000028776	TINAGL1	tubulointerstitial nephritis antigen-like 1 [Source:MGI Symbol;Acc:MGI:2137617]	4	130164454	130175122	-1	protein_coding	928.5215	0.76897499	0.189574	4.056336	4.98E-05	0.004234
ENSMUSG00000042622	MAFF	v-maf musculoaponeurotic fibrosarcoma oncogene family, protein F (avian) [Source:MGI Symbol;Acc:MGI:96910]	15	79347541	79359072	1	protein_coding	178.902	1.09501258	0.269979	4.055917	4.99E-05	0.004234
ENSMUSG00000069763	TMEM100	transmembrane protein 100 [Source:MGI Symbol;Acc:MGI:1915138]	11	90030348	90036508	1	protein_coding	695.0279	0.71727608	0.177573	4.03933	5.36E-05	0.004521
ENSMUSG00000026473	GLUL	glutamate-ammonia ligase (glutamine synthetase) [Source:MGI Symbol;Acc:MGI:95739]	1	153899944	153909723	1	protein_coding	5216.221	0.79256432	0.196451	4.034422	5.47E-05	0.004592
ENSMUSG00000067736	GM10222	predicted gene 10222 [Source:MGI Symbol;Acc:MGI:3642643]	1	24612407	24612700	-1	unprocessed_pseudogene	24342.9	-0.543165	0.134696	-4.03252	5.52E-05	0.004605
ENSMUSG00000030748	IL4RA	interleukin 4 receptor, alpha [Source:MGI Symbol;Acc:MGI:105367]	7	125552282	125579472	1	protein_coding	391.8779	0.68503416	0.170267	4.023295	5.74E-05	0.004765
ENSMUSG00000083678	GM12989	predicted gene 12989 [Source:MGI Symbol;Acc:MGI:3652245]	4	135715868	135716598	-1	processed_pseudogene	44.89586	-1.7482368	0.434712	-4.0216	5.78E-05	0.004774
ENSMUSG00000040659	EFHD2	EF hand domain containing 2 [Source:MGI Symbol;Acc:MGI:106504]	4	141858142	141874920	-1	protein_coding	2322.706	0.78783293	0.196288	4.013657	5.98E-05	0.004908
ENSMUSG00000022357	KLHL38	kelch-like 38 [Source:MGI Symbol;Acc:MGI:3045310]	15	58314573	58324169	-1	protein_coding	427.737	1.0061194	0.250737	4.012642	6.00E-05	0.004908
ENSMUSG00000044086	LMOD3	leiomodin 3 (fetal) [Source:MGI Symbol;Acc:MGI:2444169]	6	97238528	97252780	-1	protein_coding	775.0459	-0.6992916	0.174438	-4.00882	6.10E-05	0.004963
ENSMUSG00000007682	DIO2	deiodinase, iodothyronine, type II [Source:MGI Symbol;Acc:MGI:1338833]	12	90724554	90738438	-1	protein_coding	106.4131	1.26600362	0.316285	4.002728	6.26E-05	0.005067
ENSMUSG00000038417	FIG4	FIG4 homolog (S. cerevisiae) [Source:MGI Symbol;Acc:MGI:2143585]	10	41188172	41303241	-1	protein_coding	865.5734	-0.5669644	0.141895	-3.99566	6.45E-05	0.005194
ENSMUSG00000007888	CRLF1	cytokine receptor-like factor 1 [Source:MGI Symbol;Acc:MGI:1340030]	8	70493158	70504081	1	protein_coding	114.7085	1.61499388	0.404353	3.994019	6.50E-05	0.005204
ENSMUSG00000029177	CENPA	centromere protein A [Source:MGI Symbol;Acc:MGI:88375]	5	30666777	30674827	1	protein_coding	437.0977	-0.7664636	0.192023	-3.99152	6.57E-05	0.005232
ENSMUSG00000001555	FKBP10	FK506 binding protein 10 [Source:MGI Symbol;Acc:MGI:104769]	11	100415697	100424824	1	protein_coding	874.1375	0.60939925	0.152999	3.983032	6.80E-05	0.005396
ENSMUSG00000034390	CMIP	c-Maf inducing protein [Source:MGI Symbol;Acc:MGI:1921690]	8	117257019	117461503	1	protein_coding	758.4297	0.60966583	0.153295	3.977081	6.98E-05	0.0055
ENSMUSG00000039167	ELTD1	EGF, latrophilin seven transmembrane domain containing 1 [Source:MGI Symbol;Acc:MGI:2655562]	3	151437887	151545086	1	protein_coding	845.2197	0.665108	0.167275	3.976128	7.00E-05	0.0055
ENSMUSG00000026315	SERPINB8	serine (or cysteine) peptidase inhibitor, clade B, member 8 [Source:MGI Symbol;Acc:MGI:894657]	1	107590006	107610484	1	protein_coding	121.7598	1.04734091	0.263654	3.972411	7.11E-05	0.005559
ENSMUSG00000079018	LY6C1	lymphocyte antigen 6 complex, locus C1 [Source:MGI Symbol;Acc:MGI:96882]	15	75044018	75048830	-1	protein_coding	1276.765	0.67468942	0.169924	3.970525	7.17E-05	0.005576
ENSMUSG00000040219	TTC12	tetratricopeptide repeat domain 12 [Source:MGI Symbol;Acc:MGI:2444588]	9	49436963	49486225	-1	protein_coding	694.6869	-0.590424	0.148784	-3.96834	7.24E-05	0.0056
ENSMUSG00000026435	SLC45A3	solute carrier family 45, member 3 [Source:MGI Symbol;Acc:MGI:1922082]	1	131962967	131982969	1	protein_coding	328.927	0.73292833	0.185083	3.959993	7.50E-05	0.005772
ENSMUSG00000049001	NDNF	neuron-derived neurotrophic factor [Source:MGI Symbol;Acc:MGI:1915419]	6	65671590	65706930	1	protein_coding	276.7348	-0.7701138	0.194778	-3.9538	7.69E-05	0.005895
ENSMUSG00000026347	TMEM163	transmembrane protein 163 [Source:MGI Symbol;Acc:MGI:1919410]	1	127486546	127679548	-1	protein_coding	2183.321	-0.61773	0.156686	-3.94247	8.06E-05	0.006151
ENSMUSG00000015365	MOV10L1	Moloney leukemia virus 10-like 1 [Source:MGI Symbol;Acc:MGI:1891384]	15	88982909	89055152	1	protein_coding	315.3537	-0.8542054	0.217175	-3.93326	8.38E-05	0.006361
ENSMUSG00000081352	GM12389	predicted gene 12389 [Source:MGI Symbol;Acc:MGI:3650084]	4	40116816	40117233	-1	processed_pseudogene	38.13037	-2.2313284	0.567622	-3.93101	8.46E-05	0.006367
ENSMUSG00000044017	GPR133	G protein-coupled receptor 133 [Source:MGI Symbol;Acc:MGI:3041203]	5	129096750	129204599	1	protein_coding	456.8135	0.75952153	0.193225	3.93077	8.47E-05	0.006367
ENSMUSG00000001098	KCTD10	potassium channel tetramerisation domain containing 10 [Source:MGI Symbol;Acc:MGI:2141207]	5	114363567	114380515	-1	protein_coding	874.9747	0.5954324	0.151648	3.926424	8.62E-05	0.006452
ENSMUSG00000028383	HSDL2	hydroxysteroid dehydrogenase like 2 [Source:MGI Symbol;Acc:MGI:1919729]	4	59581563	59618689	1	protein_coding	2853.942	0.4862468	0.123946	3.923041	8.74E-05	0.006492
ENSMUSG00000097776	GM26795	predicted gene, 26795 [Source:MGI Symbol;Acc:MGI:5477289]	8	28593505	28595599	1	lincRNA	85.00174	-1.2819514	0.326803	-3.92271	8.76E-05	0.006492
ENSMUSG00000095562	GM21887	predicted gene, 21887 [Source:MGI Symbol;Acc:MGI:5434051]	X	170009659	170019281	1	protein_coding	387.3677	0.90877088	0.231785	3.920758	8.83E-05	0.006514
ENSMUSG00000020689	ITGB3	integrin beta 3 [Source:MGI Symbol;Acc:MGI:96612]	11	104608000	104670476	1	protein_coding	73.64802	1.370242	0.350245	3.912236	9.14E-05	0.006717
ENSMUSG00000031442	MCF2L	mcf.2 transforming sequence-like [Source:MGI Symbol;Acc:MGI:103263]	8	12873806	13020905	1	protein_coding	614.5761	0.77969807	0.19986	3.901226	9.57E-05	0.006998
ENSMUSG00000021904	SEMA3G	sema domain, immunoglobulin domain (Ig), short basic domain, secreted, (semaphorin) 3G [Source:MGI Symbol;Acc:MGI:3041242]	14	31217860	31230350	1	protein_coding	358.3736	0.7889308	0.202903	3.888213	0.000101	0.00735
ENSMUSG00000055116	ARNTL	aryl hydrocarbon receptor nuclear translocator-like [Source:MGI Symbol;Acc:MGI:1096381]	7	113207465	113314122	1	protein_coding	676.1236	0.60733865	0.156311	3.885449	0.000102	0.007389
ENSMUSG00000021390	OGN	osteoglycin [Source:MGI Symbol;Acc:MGI:109278]	13	49608071	49624500	1	protein_coding	1698.529	-0.5175012	0.133225	-3.8844	0.000103	0.007389
ENSMUSG00000073489	IFI204	interferon activated gene 204 [Source:MGI Symbol;Acc:MGI:96429]	1	173747293	173766943	-1	protein_coding	184.9551	0.92303692	0.237673	3.883641	0.000103	0.007389
ENSMUSG00000030889	VWA3A	von Willebrand factor A domain containing 3A [Source:MGI Symbol;Acc:MGI:3041229]	7	120739557	120805742	1	protein_coding	105.6214	1.20559764	0.310958	3.877042	0.000106	0.007558
ENSMUSG00000000149	GNA12	guanine nucleotide binding protein, alpha 12 [Source:MGI Symbol;Acc:MGI:95767]	5	140758408	140830431	-1	protein_coding	3557.174	0.52695471	0.135987	3.875038	0.000107	0.007586
ENSMUSG00000026489	ADCK3	aarF domain containing kinase 3 [Source:MGI Symbol;Acc:MGI:1914676]	1	180165238	180199602	-1	protein_coding	6848.175	-0.4233081	0.109423	-3.86853	0.000109	0.007757
ENSMUSG00000000889	DBH	dopamine beta hydroxylase [Source:MGI Symbol;Acc:MGI:94864]	2	27165233	27183200	1	protein_coding	160.6883	-1.2645201	0.327088	-3.86599	0.000111	0.007803
ENSMUSG00000025745	HADHA	hydroxyacyl-Coenzyme A dehydrogenase/3-ketoacyl-Coenzyme A thiolase/enoyl-Coenzyme A hydratase (trifunctional protein), alpha sub	5	30118301	30155162	-1	protein_coding	13107.28	0.4323444	0.111867	3.864814	0.000111	0.007807
ENSMUSG00000033016	NFATC1	nuclear factor of activated T cells, cytoplasmic, calcineurin dependent 1 [Source:MGI Symbol;Acc:MGI:102469]	18	80606205	80713071	-1	protein_coding	666.2086	0.56882309	0.147251	3.862938	0.000112	0.007832

ENSMUSG00000000805	CAR4	carbonic anhydrase 4 [Source:MGI Symbol;Acc:MGI:1096574]	11	84957786	84966044	1	protein_coding	211.3028	0.99667026	0.258104	3.8615	0.000113	0.007844
ENSMUSG00000031382	ASB11	ankyrin repeat and SOCS box-containing 11 [Source:MGI Symbol;Acc:MGI:1916104]	X	164436994	164459170	1	protein_coding	1351.767	0.59895643	0.15539	3.854526	0.000116	0.008036
ENSMUSG00000068922	MSTO1	misato homolog 1 (Drosophila) [Source:MGI Symbol;Acc:MGI:2385175]	3	88905107	88913999	-1	protein_coding	233.8907	-0.8220125	0.213377	-3.8524	0.000117	0.008071
ENSMUSG00000027875	HMGCS2	3-hydroxy-3-methylglutaryl-Coenzyme A synthase 2 [Source:MGI Symbol;Acc:MGI:101939]	3	98280435	98310738	1	protein_coding	597.0823	1.82955975	0.47543	3.848218	0.000119	0.008175
ENSMUSG00000056749	NFIL3	nuclear factor, interleukin 3, regulated [Source:MGI Symbol;Acc:MGI:109495]	13	52967209	52981073	-1	protein_coding	408.8574	0.77173584	0.200725	3.844745	0.000121	0.008254
ENSMUSG00000022893	ADAMTS1	a disintegrin-like and metallopeptidase (reprolysin type) with thrombospondin type 1 motif, 1 [Source:MGI Symbol;Acc:MGI:109249]	16	85793827	85803113	-1	protein_coding	783.5584	0.84933139	0.220974	3.843575	0.000121	0.008254
ENSMUSG00000056666	RETSAT	retinol saturase (all trans retinol 13,14 reductase) [Source:MGI Symbol;Acc:MGI:1914692]	6	72598475	72608425	1	protein_coding	1192.749	0.71392691	0.1858	3.842458	0.000122	0.008254
ENSMUSG00000059461	GM7331	predicted gene 7331 [Source:MGI Symbol;Acc:MGI:3644702]	X	162566857	162567243	1	processed_pseudogene	330.3787	-1.1973303	0.311669	-3.84167	0.000122	0.008254
ENSMUSG00000001211	AGPAT3	1-acylglycerol-3-phosphate O-acyltransferase 3 [Source:MGI Symbol;Acc:MGI:1336186]	10	78269178	78352489	-1	protein_coding	3474.067	0.48628654	0.126768	3.836039	0.000125	0.008384
ENSMUSG00000029804	HERC3	hect domain and RLD 3 [Source:MGI Symbol;Acc:MGI:1921248]	6	58831465	58920398	1	protein_coding	512.8131	-0.6355789	0.165736	-3.83488	0.000126	0.008384
ENSMUSG00000002997	PRKAR2B	protein kinase, cAMP dependent regulatory, type II beta [Source:MGI Symbol;Acc:MGI:97760]	12	31958476	32061296	-1	protein_coding	187.1492	1.27119252	0.331528	3.834347	0.000126	0.008384
ENSMUSG00000101389	MS4A4A	membrane-spanning 4-domains, subfamily A, member 4A [Source:MGI Symbol;Acc:MGI:3643932]	19	11375523	11392790	1	protein_coding	49.218	1.50511477	0.3926	3.833706	0.000126	0.008384
ENSMUSG00000053113	SOCS3	suppressor of cytokine signaling 3 [Source:MGI Symbol;Acc:MGI:1201791]	11	117966079	117970047	-1	protein_coding	238.5542	0.98987355	0.259224	3.818606	0.000134	0.008865
ENSMUSG00000034121	MKS1	Meckel syndrome, type 1 [Source:MGI Symbol;Acc:MGI:3584243]	11	87853215	87863803	1	protein_coding	164.8663	-0.9674406	0.253454	-3.81703	0.000135	0.008865
ENSMUSG00000057880	ABAT	4-aminobutyrate aminotransferase [Source:MGI Symbol;Acc:MGI:2443582]	16	8513429	8621568	1	protein_coding	1070.023	-0.564958	0.148016	-3.81688	0.000135	0.008865
ENSMUSG00000022215	FITM1	fat storage-inducing transmembrane protein 1 [Source:MGI Symbol;Acc:MGI:1915930]	14	55575617	55576952	1	protein_coding	845.0336	-0.5357407	0.140524	-3.81245	0.000138	0.008989
ENSMUSG00000048546	TOB2	transducer of ERBB2, 2 [Source:MGI Symbol;Acc:MGI:1888525]	15	81848270	81858326	-1	protein_coding	1265.076	0.52369803	0.137693	3.803373	0.000143	0.009278
ENSMUSG00000029135	FOSL2	fos-like antigen 2 [Source:MGI Symbol;Acc:MGI:102858]	5	32136472	32157831	1	protein_coding	698.6163	0.77323934	0.203345	3.8026	0.000143	0.009278
ENSMUSG00000074064	MLYCD	malonyl-CoA decarboxylase [Source:MGI Symbol;Acc:MGI:1928485]	8	119394878	119411102	1	protein_coding	1523.773	0.66042594	0.173802	3.799877	0.000145	0.009323
ENSMUSG00000041216	CLVS1	clavesin 1 [Source:MGI Symbol;Acc:MGI:1921688]	4	9269293	9451691	1	protein_coding	106.1342	-1.3255293	0.348912	-3.79904	0.000145	0.009323
ENSMUSG00000000290	ITGB2	integrin beta 2 [Source:MGI Symbol;Acc:MGI:96611]	10	77530252	77565708	1	protein_coding	241.4679	0.99071751	0.260826	3.798387	0.000146	0.009323
ENSMUSG00000069094	PDE7A	phosphodiesterase 7A [Source:MGI Symbol;Acc:MGI:1202402]	3	19223108	19311322	-1	protein_coding	686.3445	0.71988881	0.189751	3.793851	0.000148	0.009457
ENSMUSG00000026163	SPHKAP	SPHK1 interactor, AKAP domain containing [Source:MGI Symbol;Acc:MGI:1924879]	1	83254139	83408200	-1	protein_coding	215.1765	-0.9166117	0.242462	-3.78044	0.000157	0.009942
ENSMUSG00000021957	TKT	transketolase [Source:MGI Symbol;Acc:MGI:105992]	14	30548359	30574722	1	protein_coding	912.9249	-0.5718837	0.151374	-3.77795	0.000158	0.010002
ENSMUSG00000037594	BC022687	cDNA sequence BC022687 [Source:MGI Symbol;Acc:MGI:2443738]	12	112808975	112816245	1	protein_coding	249.6294	-0.7599795	0.201215	-3.77695	0.000159	0.010003
ENSMUSG00000074136	4930513N10RIK	RIKEN cDNA 4930513N10 gene [Source:MGI Symbol;Acc:MGI:2443064]	8	95806830	95821728	1	processed_transcript	185.368	-0.8915231	0.236541	-3.769	0.000164	0.010286
ENSMUSG00000066952	MYO1H	myosin 1H [Source:MGI Symbol;Acc:MGI:1914674]	5	114314941	114364576	1	protein_coding	146.3885	-1.2569514	0.333828	-3.76527	0.000166	0.010365
ENSMUSG00000032259	DRD2	dopamine receptor D2 [Source:MGI Symbol;Acc:MGI:94924]	9	49340662	49407214	1	protein_coding	48.95777	-1.6443716	0.436919	-3.76356	0.000168	0.010365
ENSMUSG00000007877	TCAP	titin-cap [Source:MGI Symbol;Acc:MGI:1330233]	11	98383811	98384953	1	protein_coding	10653.08	-0.6006989	0.159618	-3.76335	0.000168	0.010365
ENSMUSG00000024778	FAS	Fas (TNF receptor superfamily member 6) [Source:MGI Symbol;Acc:MGI:95484]	19	34290659	34327770	1	protein_coding	252.5742	0.87896154	0.233568	3.76319	0.000168	0.010365
ENSMUSG00000025885	MYO5B	myosin VB [Source:MGI Symbol;Acc:MGI:106598]	18	74440936	74771493	1	protein_coding	320.7373	0.73107128	0.194597	3.75685	0.000172	0.01059
ENSMUSG00000071342	LSMEM1	leucine-rich single-pass membrane protein 1 [Source:MGI Symbol;Acc:MGI:2685735]	12	40176386	40199315	-1	protein_coding	186.8731	-0.8695997	0.231973	-3.74872	0.000178	0.01087
ENSMUSG00000026113	INPP4A	inositol polyphosphate-4-phosphatase, type I [Source:MGI Symbol;Acc:MGI:1931123]	1	37299865	37410736	1	protein_coding	660.9459	0.7464241	0.19914	3.748241	0.000178	0.01087
ENSMUSG00000031385	PLXNB3	plexin B3 [Source:MGI Symbol;Acc:MGI:2154240]	X	73757090	73772514	1	protein_coding	174.1788	-0.9017892	0.240643	-3.74742	0.000179	0.01087
ENSMUSG00000045092	S1PR1	sphingosine-1-phosphate receptor 1 [Source:MGI Symbol;Acc:MGI:1096355]	3	115710433	115715055	-1	protein_coding	1437.759	0.5005142	0.133661	3.744654	0.000181	0.010948
ENSMUSG00000097461	GM26735	predicted gene, 26735 [Source:MGI Symbol;Acc:MGI:5477229]	13	28943048	28951671	1	lincRNA	441.1162	-0.8740833	0.233574	-3.7422	0.000182	0.011014
ENSMUSG00000021094	DHRS7	dehydrogenase/reductase (SDR family) member 7 [Source:MGI Symbol;Acc:MGI:1913625]	12	72650356	72664828	-1	protein_coding	1036.815	-0.5038545	0.134869	-3.73588	0.000187	0.011252
ENSMUSG00000026888	GRB14	growth factor receptor bound protein 14 [Source:MGI Symbol;Acc:MGI:1355324]	2	64912476	65024987	-1	protein_coding	1741.825	-0.5003159	0.134009	-3.73345	0.000189	0.011318
ENSMUSG00000029101	RGS12	regulator of G-protein signaling 12 [Source:MGI Symbol;Acc:MGI:1918979]	5	34949445	35039644	1	protein_coding	1165.46	-0.6189459	0.165996	-3.72869	0.000192	0.011491
ENSMUSG00000050440	HAMP	hepcidin antimicrobial peptide [Source:MGI Symbol;Acc:MGI:1933533]	7	30942371	30944017	-1	protein_coding	637.752	-0.820975	0.22048	-3.72358	0.000196	0.011683
ENSMUSG00000090145	UGT1A6B	UDP glucuronosyltransferase 1 family, polypeptide A6B [Source:MGI Symbol;Acc:MGI:3580629]	1	88103252	88219003	1	protein_coding	13.63933	3.78827164	1.018411	3.719785	0.000199	0.011779
ENSMUSG00000054252	FGFR3	fibroblast growth factor receptor 3 [Source:MGI Symbol;Acc:MGI:95524]	5	33721724	33737068	1	protein_coding	171.3433	0.86471105	0.232529	3.718728	0.0002	0.011779
ENSMUSG00000048142	NAT8L	N-acetyltransferase 8-like [Source:MGI Symbol;Acc:MGI:2447776]	5	33995984	34005916	1	protein_coding	296.195	-0.6977855	0.187643	-3.71868	0.0002	0.011779
ENSMUSG00000045975	C2CD2	C2 calcium-dependent domain containing 2 [Source:MGI Symbol;Acc:MGI:1891883]	16	97855210	97922633	-1	protein_coding	391.5209	0.67716758	0.182204	3.716532	0.000202	0.011836
ENSMUSG00000059895	PTP4A3	protein tyrosine phosphatase 4a3 [Source:MGI Symbol;Acc:MGI:1277098]	15	73723145	73758766	1	protein_coding	4451.655	0.58038076	0.156435	3.710039	0.000207	0.0121
ENSMUSG00000027809	ETFDH	electron transferring flavoprotein, dehydrogenase [Source:MGI Symbol;Acc:MGI:106100]	3	79603788	79629500	-1	protein_coding	6187.384	0.45643657	0.123201	3.704812	0.000212	0.012307
ENSMUSG00000032602	SLC25A20	solute carrier family 25 (mitochondrial carnitine/acylcarnitine translocase), member 20 [Source:MGI Symbol;Acc:MGI:1928738]	9	108662088	108684641	1	protein_coding	1759.781	0.57943132	0.156649	3.698907	0.000217	0.012551
ENSMUSG00000025221	KCNIP2	Kv channel-interacting protein 2 [Source:MGI Symbol;Acc:MGI:2135916]	19	45791839	45816061	-1	protein_coding	604.894	-0.6434977	0.174106	-3.69601	0.000219	0.012649
ENSMUSG00000024561	MBD1	methyl-CpG binding domain protein 1 [Source:MGI Symbol;Acc:MGI:1333811]	18	74268272	74282685	1	protein_coding	357.0327	0.72188534	0.195513	3.692266	0.000222	0.012761
ENSMUSG00000021868	PPIF	peptidylprolyl isomerase F (cyclophilin F) [Source:MGI Symbol;Acc:MGI:2145814]	14	25694154	25700468	1	protein_coding	1534.151	-0.6731861	0.182384	-3.69103	0.000223	0.012761
ENSMUSG00000035105	EGLN3	egl-9 family hypoxia-inducible factor 3 [Source:MGI Symbol;Acc:MGI:1932288]	12	54178981	54203860	-1	protein_coding	959.5032	0.66965732	0.181429	3.691019	0.000223	0.012761
ENSMUSG00000059498	FCGR3	Fc receptor, IgG, low affinity III [Source:MGI Symbol;Acc:MGI:95500]	1	171051174	171064935	-1	protein_coding	342.699	0.73825716	0.200064	3.69011	0.000224	0.012761
ENSMUSG00000028607	CPT2	carnitine palmitoyltransferase 2 [Source:MGI Symbol;Acc:MGI:109176]	4	107903981	107923610	-1	protein_coding	2659.088	0.52649963	0.142955	3.682966	0.000231	0.013077
ENSMUSG00000039844	RAPGEF1	Rap guanine nucleotide exchange factor (GEF) 1 [Source:MGI Symbol;Acc:MGI:104580]	2	29619720	29740978	1	protein_coding	2129.318	0.57350207	0.155932	3.677892	0.000235	0.013293
ENSMUSG00000064356	MT-ATP8	mitochondrially encoded ATP synthase 8 [Source:MGI Symbol;Acc:MGI:99926]	MT	7766	7969	1	protein_coding	15733.87	-0.4766198	0.129879	-3.66972	0.000243	0.013677
ENSMUSG00000058056	PALLD	palladin, cytoskeletal associated protein [Source:MGI Symbol;Acc:MGI:1919583]	8	61513246	61902690	-1	protein_coding	2647.578	0.65404646	0.178594	3.662206	0.00025	0.014035
ENSMUSG00000030087	KLF15	Kruppel-like factor 15 [Source:MGI Symbol;Acc:MGI:1929988]	6	90462576	90475238	1	protein_coding	904.0514	0.70210376	0.191961	3.657529	0.000255	0.014243
ENSMUSG00000070803	CITED4	Cbp/p300-interacting transactivator, with Glu/Asp-rich carboxy-terminal domain, 4 [Source:MGI Symbol;Acc:MGI:1861694]	4	120666572	120667820	1	protein_coding	959.4701	-0.8417128	0.230599	-3.65012	0.000262	0.014609
ENSMUSG00000021750	FAM107A	family with sequence similarity 107, member A [Source:MGI Symbol;Acc:MGI:3041256]	14	8296274	8318023	-1	protein_coding	132.846	1.01005363	0.277068	3.645504	0.000267	0.014822
ENSMUSG00000032609	KLHDC8B	kelch domain containing 8B [Source:MGI Symbol;Acc:MGI:1925517]	9	108447636	108461581	-1	protein_coding	357.4778	-0.6799005	0.186592	-3.64379	0.000269	0.014869
ENSMUSG00000001131	TIMP1	tissue inhibitor of metalloproteinase 1 [Source:MGI Symbol;Acc:MGI:98752]	X	20870166	20874735	1	protein_coding	166.4837	0.93574151	0.257144	3.638985	0.000274	0.015097
ENSMUSG00000078515	DDI2	DNA-damage inducible protein 2 [Source:MGI Symbol;Acc:MGI:1917244]	4	141677549	141723419	-1	protein_coding	853.6199	0.54175165	0.149101	3.633463	0.00028	0.015271
ENSMUSG00000025316	BANP	BTG3 associated nuclear protein [Source:MGI Symbol;Acc:MGI:1889023]	8	121949750	122029258	1	protein_coding	212.6209	0.98035158	0.269819	3.633372	0.00028	0.015271
ENSMUSG00000030214	PLBD1	phospholipase B domain containing 1 [Source:MGI Symbol;Acc:MGI:1914107]	6	136612070	136661928	-1	protein_coding	386.9141	0.78327451	0.215621	3.632643	0.000281	0.015271
ENSMUSG00000050022	AMZ1	archaelysin family metallopeptidase 1 [Source:MGI Symbol;Acc:MGI:2442258]	5	140724127	140761439	1	protein_coding	123.7832	1.42063491	0.391181	3.631658	0.000282	0.015271
ENSMUSG00000008932	SLC1A7	solute carrier family 1 (glutamate transporter), member 7 [Source:MGI Symbol;Acc:MGI:2444087]	4	107968332	108013532	1	protein_coding	89.90613	-1.0934503	0.301102	-3.6315	0.000282	0.015271
ENSMUSG00000027244	ATG13	autophagy related 13 [Source:MGI Symbol;Acc:MGI:1196429]	2	91674618	91710576	-1	protein_coding	1350.01	0.52431802	0.144411	3.630725	0.000283	0.015271
ENSMUSG00000021185	9030617O03RIK	RIKEN cDNA 9030617O03 gene [Source:MGI Symbol;Acc:MGI:2444813]	12	100779057	100896981	1	protein_coding	1139.869	-0.5252086	0.14478	-3.62762	0.000286	0.015374
ENSMUSG00000024164	C3	complement component 3 [Source:MGI Symbol;Acc:MGI:88227]	17	57203970	57228136	-1	protein_coding	9180.181	0.51363831	0.141605	3.627252	0.000286	0.015374
ENSMUSG00000038508	GDF15	growth differentiation factor 15 [Source:MGI Symbol;Acc:MGI:1346047]	8	70629393	70632456	-1	protein_coding	48.76347	1.84082569	0.507828	3.624899	0.000289	0.015463
ENSMUSG00000024413	NPC1	Niemann-Pick type C1 [Source:MGI Symbol;Acc:MGI:1097712]	18	12189693	12236400	-1	protein_coding	1025.945	0.70882931	0.195648	3.622974	0.000291	0.015526
ENSMUSG00000050974	GM9847	predicted pseudogene 9847 [Source:MGI Symbol;Acc:MGI:3642840]	12	14494561	14495157	1	protein_coding	13.8893	-3.9289351	1.084955	-3.62129	0.000293	0.015576
ENSMUSG00000050592	FAM78A	family with sequence similarity 78, member A [Source:MGI Symbol;Acc:MGI:2443569]	2	32050055	32084857	-1	protein_coding	649.2662	-0.6038696	0.166934	-3.61742	0.000298	0.015757
ENSMUSG00000000958	SLC7A7	solute carrier family 7 (cationic amino acid transporter, y+ system), member 7 [Source:MGI Symbol;Acc:MGI:1337120]	14	54369444	54409472	-1	protein_coding	1114.511	-0.5182493	0.143624	-3.60839	0.000308	0.016262
ENSMUSG00000029502	GOLGA3	golgi autoantigen, golgin subfamily a, 3 [Source:MGI Symbol;Acc:MGI:96958]	5	110176701	110226470	1	protein_coding	1251.145	0.52254454	0.14486	3.607246	0.000309	0.01628
ENSMUSG00000027408	CPXM1	carboxypeptidase X 1 (M14 family) [Source:MGI Symbol;Acc:MGI:1934569]	2	130390775	130397574	-1	protein_coding	313.6454	-0.7943461	0.220372	-3.60457	0.000313	0.016395
ENSMUSG00000047731	WBP1L	WW domain binding protein 1 like [Source:MGI Symbol;Acc:MGI:107577]	19	46599084	46657389	1	protein_coding	1787.038	0.45895018	0.127447	3.6011	0.000317	0.016539

ENSMUSG00000003665	HAS1	hyaluronan synthase1 [Source:MGI Symbol;Acc:MGI:106590]	17	17843328	17855188	-1	protein_coding	37.42205	1.7470238	0.485206	3.600585	0.000318	0.016539
ENSMUSG00000037206	ISLR	immunoglobulin superfamily containing leucine-rich repeat [Source:MGI Symbol;Acc:MGI:1349645]	9	58156265	58159221	-1	protein_coding	1049.667	-0.6113966	0.170141	-3.59347	0.000326	0.016942
ENSMUSG00000087331	1810021B22RIK	RIKEN cDNA 1810021B22 gene [Source:MGI Symbol;Acc:MGI:1916370]	15	89071196	89075852	-1	processed_transcript	79.48194	-1.2156135	0.339703	-3.57846	0.000346	0.017887
ENSMUSG00000019836	AMD-PS4	S-adenosylmethionine decarboxylase, pseudogene 4 [Source:MGI Symbol;Acc:MGI:3650439]	4	51253025	51254027	-1	processed_pseudogene	61.8406	1.35106348	0.378364	3.570805	0.000356	0.018271
ENSMUSG00000039063	ECHDC3	enoyl Coenzyme A hydratase domain containing 3 [Source:MGI Symbol;Acc:MGI:1915106]	2	6188465	6213033	-1	protein_coding	309.3283	-0.6518193	0.182551	-3.57061	0.000356	0.018271
ENSMUSG00000009687	FXYD5	FXYD domain-containing ion transport regulator 5 [Source:MGI Symbol;Acc:MGI:1201785]	7	31032722	31042481	-1	protein_coding	488.8673	0.68675672	0.192403	3.569367	0.000358	0.018271
ENSMUSG00000030317	TIMP4	tissue inhibitor of metalloproteinase 4 [Source:MGI Symbol;Acc:MGI:109125]	6	115245616	115251849	-1	protein_coding	315.625	0.93128946	0.26094	3.568973	0.000358	0.018271
ENSMUSG00000020682	MMP28	matrix metallopeptidase 28 (epilysin) [Source:MGI Symbol;Acc:MGI:2153062]	11	83440768	83463071	-1	protein_coding	181.4657	0.8188806	0.229463	3.568687	0.000359	0.018271
ENSMUSG00000046402	RBP1	retinol binding protein 1, cellular [Source:MGI Symbol;Acc:MGI:97876]	9	98422961	98446575	1	protein_coding	466.611	-0.5635998	0.158205	-3.56247	0.000367	0.01865
ENSMUSG00000103057	RP23-81C12.2		17	39843753	39844940	-1	TEC	324.3877	-0.9399828	0.264246	-3.55722	0.000375	0.018966
ENSMUSG00000028989	ANGPTL7	angiopoietin-like 7 [Source:MGI Symbol;Acc:MGI:3605801]	4	148495183	148500460	-1	protein_coding	87.74893	-1.242404	0.349523	-3.55457	0.000379	0.019098
ENSMUSG00000039879	HECA	headcase homolog (Drosophila) [Source:MGI Symbol;Acc:MGI:2685715]	10	17900466	17948067	-1	protein_coding	505.9666	0.91785947	0.25833	3.553046	0.000381	0.019148
ENSMUSG00000027187	CAT	catalase [Source:MGI Symbol;Acc:MGI:88271]	2	103453849	103485160	-1	protein_coding	2694.767	0.66542628	0.187431	3.550242	0.000385	0.019292
ENSMUSG00000024806	MLANA	melan-A [Source:MGI Symbol;Acc:MGI:108454]	19	29697921	29708448	1	protein_coding	686.305	-0.6081819	0.171356	-3.54922	0.000386	0.019306
ENSMUSG00000064369	MT-TE	mitochondrially encoded tRNA glutamic acid [Source:MGI Symbol;Acc:MGI:102488]	MT	14071	14139	-1	Mt_tRNA	3108.398	-0.4829619	0.136376	-3.54141	0.000398	0.019825
ENSMUSG00000021456	FBP2	fructose bisphosphatase 2 [Source:MGI Symbol;Acc:MGI:95491]	13	62836884	62858400	-1	protein_coding	99.37881	1.05681127	0.298667	3.538424	0.000403	0.019988
ENSMUSG00000044991	1110034G24RIK	RIKEN cDNA 1110034G24 gene [Source:MGI Symbol;Acc:MGI:1920997]	2	132686931	132751055	1	protein_coding	412.0224	0.92543497	0.261816	3.534673	0.000408	0.020211
ENSMUSG00000053964	LGALS4	lectin, galactose binding, soluble 4 [Source:MGI Symbol;Acc:MGI:107536]	7	28833854	28841708	1	protein_coding	859.4951	1.76783663	0.500552	3.531776	0.000413	0.020371
ENSMUSG00000046442	PPM1E	protein phosphatase 1E (PP2C domain containing) [Source:MGI Symbol;Acc:MGI:2444096]	11	87226906	87359023	-1	protein_coding	327.6559	0.76938324	0.218612	3.519401	0.000433	0.021279
ENSMUSG00000027510	RBM38	RNA binding motif protein 38 [Source:MGI Symbol;Acc:MGI:1889294]	2	173020498	173034734	1	protein_coding	2055.912	0.65971892	0.187576	3.51707	0.000436	0.021401
ENSMUSG00000005628	TMOD4	tropomodulin 4 [Source:MGI Symbol;Acc:MGI:1355285]	3	95124476	95129209	1	protein_coding	139.2547	-1.0095599	0.287165	-3.51561	0.000439	0.021413
ENSMUSG00000028957	PER3	period circadian clock 3 [Source:MGI Symbol;Acc:MGI:1277134]	4	151003652	151044665	-1	protein_coding	225.7311	-0.7393985	0.210353	-3.51504	0.00044	0.021413
ENSMUSG00000022220	ADCY4	adenylate cyclase 4 [Source:MGI Symbol;Acc:MGI:99674]	14	55769058	55784042	-1	protein_coding	686.2855	0.62614982	0.178163	3.514483	0.000441	0.021413
ENSMUSG00000101625	GM29371	predicted gene 29371 [Source:MGI Symbol;Acc:MGI:5580077]	1	87080171	87096418	1	lincRNA	11.10919	4.04297471	1.151176	3.51204	0.000445	0.021545
ENSMUSG00000026670	UAP1	UDP-N-acetylglucosamine pyrophosphorylase 1 [Source:MGI Symbol;Acc:MGI:1334459]	1	170141938	170174957	-1	protein_coding	1202.123	0.51974379	0.14811	3.509185	0.000449	0.021711
ENSMUSG00000014791	ELMO3	engulfment and cell motility 3 [Source:MGI Symbol;Acc:MGI:2679007]	8	105305601	105310623	1	protein_coding	98.57021	-1.0430401	0.297507	-3.50593	0.000455	0.021912
ENSMUSG00000026833	OLFM1	olfactomedin 1 [Source:MGI Symbol;Acc:MGI:1860437]	2	28192992	28230736	1	protein_coding	1848.904	-0.4708613	0.134348	-3.50479	0.000457	0.02194
ENSMUSG00000018574	ACADVL	acyl-Coenzyme A dehydrogenase, very long chain [Source:MGI Symbol;Acc:MGI:895149]	11	70010183	70015411	-1	protein_coding	10063.85	0.42489354	0.121401	3.499912	0.000465	0.022278
ENSMUSG00000046223	PLAUR	plasminogen activator, urokinase receptor [Source:MGI Symbol;Acc:MGI:97612]	7	24462500	24475873	1	protein_coding	58.71419	1.40081503	0.400782	3.495201	0.000474	0.022602
ENSMUSG00000022146	OSMR	oncostatin M receptor [Source:MGI Symbol;Acc:MGI:1330819]	15	6813577	6874969	-1	protein_coding	616.9124	0.59783239	0.17109	3.494251	0.000475	0.022602
ENSMUSG00000026471	MR1	major histocompatibility complex, class I-related [Source:MGI Symbol;Acc:MGI:1195463]	1	155127277	155146814	-1	protein_coding	546.9764	-0.5617933	0.160803	-3.49367	0.000476	0.022602
ENSMUSG00000026785	PKN3	protein kinase N3 [Source:MGI Symbol;Acc:MGI:2388285]	2	30077684	30091022	1	protein_coding	404.6916	0.64123581	0.184165	3.48186	0.000498	0.023532
ENSMUSG00000030116	MFAP5	microfibrillar associated protein 5 [Source:MGI Symbol;Acc:MGI:1354387]	6	122505845	122529290	1	protein_coding	852.8767	0.5352164	0.15374	3.4813	0.000499	0.023532
ENSMUSG00000060862	ZBTB40	zinc finger and BTB domain containing 40 [Source:MGI Symbol;Acc:MGI:2682254]	4	136979732	137048801	-1	protein_coding	177.2673	0.84058364	0.241758	3.476962	0.000507	0.023846
ENSMUSG00000013076	AMOTL1	angiomotin-like 1 [Source:MGI Symbol;Acc:MGI:1922973]	9	14541966	14615483	-1	protein_coding	1913.818	0.51725207	0.148889	3.474078	0.000513	0.024032
ENSMUSG00000037145	2210407C18RIK	RIKEN cDNA 2210407C18 gene [Source:MGI Symbol;Acc:MGI:1925604]	11	58608204	58616075	-1	protein_coding	158.771	2.74723666	0.791079	3.472772	0.000515	0.024079
ENSMUSG00000033863	KLF9	Kruppel-like factor 9 [Source:MGI Symbol;Acc:MGI:1333856]	19	23141226	23166911	1	protein_coding	1692.669	0.56065573	0.161569	3.470067	0.00052	0.024252
ENSMUSG00000024677	MS4A6B	membrane-spanning 4-domains, subfamily A, member 6B [Source:MGI Symbol;Acc:MGI:1917024]	19	11516512	11531256	1	protein_coding	120.0449	1.08913952	0.31423	3.466057	0.000528	0.024518
ENSMUSG00000021903	GALNT15	UDP-N-acetyl-alpha-D-galactosamine:polypeptide N-acetylgalactosaminyltransferase 15 [Source:MGI Symbol;Acc:MGI:1926004]	14	32028989	32062197	1	protein_coding	879.8357	0.79907516	0.230576	3.465566	0.000529	0.024518
ENSMUSG00000000275	TRIM25	tripartite motif-containing 25 [Source:MGI Symbol;Acc:MGI:102749]	11	88999376	89020293	1	protein_coding	605.2006	0.58217456	0.168124	3.46277	0.000535	0.024702
ENSMUSG00000026639	LAMB3	laminin, beta 3 [Source:MGI Symbol;Acc:MGI:99915]	1	193207699	193343878	1	protein_coding	659.4612	-0.5798158	0.167554	-3.46048	0.000539	0.024842
ENSMUSG00000037851	IARS	isoleucine-tRNA synthetase [Source:MGI Symbol;Acc:MGI:2145219]	13	49682100	49734267	1	protein_coding	1082.967	0.52258037	0.151123	3.45797	0.000544	0.025002
ENSMUSG00000008348	UBC	ubiquitin C [Source:MGI Symbol;Acc:MGI:98889]	5	125385965	125390202	-1	protein_coding	20145.78	0.39377274	0.113977	3.454844	0.000551	0.025221
ENSMUSG00000038615	NFE2L1	nuclear factor, erythroid derived 2,-like 1 [Source:MGI Symbol;Acc:MGI:99421]	11	96817414	96829968	-1	protein_coding	8299.543	0.39929499	0.115722	3.450458	0.00056	0.02556
ENSMUSG00000030109	SLC6A12	solute carrier family 6 (neurotransmitter transporter, betaine/GABA), member 12 [Source:MGI Symbol;Acc:MGI:95628]	6	121343076	121365775	1	protein_coding	10.00486	4.88418468	1.416944	3.446985	0.000567	0.025817
ENSMUSG00000065979	CPPED1	calcineurin-like phosphoesterase domain containing 1 [Source:MGI Symbol;Acc:MGI:2443300]	16	11803721	11909445	-1	protein_coding	838.8459	-0.4852903	0.140916	-3.44382	0.000574	0.026047
ENSMUSG00000024665	FADS2	fatty acid desaturase 2 [Source:MGI Symbol;Acc:MGI:1930079]	19	10064164	10101503	-1	protein_coding	161.4528	-0.8451394	0.246079	-3.43442	0.000594	0.026891
ENSMUSG00000072949	ACOT1	acyl-CoA thioesterase 1 [Source:MGI Symbol;Acc:MGI:1349396]	12	84009502	84017670	1	protein_coding	674.9719	2.10089046	0.611999	3.432834	0.000597	0.026972
ENSMUSG00000022041	CHRNA2	cholinergic receptor, nicotinic, alpha polypeptide 2 (neuronal) [Source:MGI Symbol;Acc:MGI:87886]	14	66140960	66152948	1	protein_coding	19.66653	2.45854983	0.716443	3.431607	0.0006	0.027018
ENSMUSG00000003617	CP	ceruloplasmin [Source:MGI Symbol;Acc:MGI:88476]	3	19957054	20009145	1	protein_coding	3324.836	0.52387944	0.152919	3.425853	0.000613	0.027519
ENSMUSG00000003032	KLF4	Kruppel-like factor 4 (gut) [Source:MGI Symbol;Acc:MGI:1342287]	4	55527143	55532466	-1	protein_coding	1004.61	0.57384063	0.16763	3.423258	0.000619	0.027705
ENSMUSG00000001018	SNAPIN	SNAP-associated protein [Source:MGI Symbol;Acc:MGI:1333745]	3	90488026	90491033	-1	protein_coding	872.0294	-0.4863998	0.142523	-3.41278	0.000643	0.028712
ENSMUSG00000032264	ZW10	zw10 kinetochore protein [Source:MGI Symbol;Acc:MGI:1349478]	9	49055581	49078772	1	protein_coding	408.2352	0.59438313	0.174334	3.409451	0.000651	0.028983
ENSMUSG00000064202	4430402I18RIK	RIKEN cDNA 4430402I18 gene [Source:MGI Symbol;Acc:MGI:1918036]	19	28893042	28967800	-1	protein_coding	310.2499	-0.6462161	0.189773	-3.40521	0.000661	0.029355
ENSMUSG00000068165	GM10233	predicted pseudogene 10233 [Source:MGI Symbol;Acc:MGI:3704447]	14	66044988	66045877	1	processed_pseudogene	502.5297	-0.8146448	0.239837	-3.39666	0.000682	0.030203
ENSMUSG00000039154	SHD	src homology 2 domain-containing transforming protein D [Source:MGI Symbol;Acc:MGI:1099461]	17	55970467	55976617	1	protein_coding	67.92441	-1.1378579	0.335423	-3.39231	0.000693	0.030603
ENSMUSG00000046805	MPEG1	macrophage expressed gene 1 [Source:MGI Symbol;Acc:MGI:1333743]	19	12460779	12465284	1	protein_coding	420.0413	0.77403522	0.228273	3.390835	0.000697	0.030682
ENSMUSG00000035992	FNIP1	folliculin interacting protein 1 [Source:MGI Symbol;Acc:MGI:2444668]	11	54438199	54518235	1	protein_coding	690.1953	0.64018807	0.189046	3.386416	0.000708	0.031095
ENSMUSG00000031710	UCP1	uncoupling protein 1 (mitochondrial, proton carrier) [Source:MGI Symbol;Acc:MGI:98894]	8	83290348	83298456	1	protein_coding	288.6121	3.92983987	1.161003	3.384867	0.000712	0.031185
ENSMUSG00000048442	SMIM5	small integral membrane protein 5 [Source:MGI Symbol;Acc:MGI:1913778]	11	115899966	115906269	1	protein_coding	177.9907	-0.7764377	0.229696	-3.38029	0.000724	0.031555
ENSMUSG00000057101	ZFP180	zinc finger protein 180 [Source:MGI Symbol;Acc:MGI:1923701]	7	24081924	24107698	1	protein_coding	482.2704	0.55056879	0.162899	3.379823	0.000725	0.031555
ENSMUSG00000027329	SPEF1	sperm flagellar 1 [Source:MGI Symbol;Acc:MGI:3513546]	2	131170261	131187282	-1	protein_coding	175.1581	0.81247904	0.240423	3.379374	0.000727	0.031555
ENSMUSG00000019254	PPP1R12C	protein phosphatase 1, regulatory (inhibitor) subunit 12C [Source:MGI Symbol;Acc:MGI:1924258]	7	4481520	4501680	-1	protein_coding	1728.974	0.44590914	0.132089	3.375833	0.000736	0.031877
ENSMUSG00000031698	MYLK3	myosin light chain kinase 3 [Source:MGI Symbol;Acc:MGI:2443063]	8	85324303	85386345	-1	protein_coding	12401.65	-0.3636562	0.107804	-3.3733	0.000743	0.032032
ENSMUSG00000037095	LRG1	leucine-rich alpha-2-glycoprotein 1 [Source:MGI Symbol;Acc:MGI:1924155]	17	56119680	56121946	-1	protein_coding	631.4656	0.87533388	0.259535	3.372706	0.000744	0.032032
ENSMUSG00000022579	GPIHBP1	GPI-anchored HDL-binding protein 1 [Source:MGI Symbol;Acc:MGI:1915703]	15	75596628	75599481	1	protein_coding	692.1477	0.54534905	0.161716	3.372259	0.000746	0.032032
ENSMUSG00000039109	F13A1	coagulation factor XIII, A1 subunit [Source:MGI Symbol;Acc:MGI:1921395]	13	36867178	37050244	-1	protein_coding	1078.662	0.667181	0.197964	3.370209	0.000751	0.032185
ENSMUSG00000044534	ACKR2	atypical chemokine receptor 2 [Source:MGI Symbol;Acc:MGI:1891697]	9	121898464	121911064	1	protein_coding	79.82314	1.11618377	0.331306	3.369042	0.000754	0.032235
ENSMUSG00000041959	S100A10	S100 calcium binding protein A10 (calpactin) [Source:MGI Symbol;Acc:MGI:1339468]	3	93555080	93564643	1	protein_coding	561.22	0.57227234	0.169978	3.36675	0.000761	0.032417
ENSMUSG00000037348	PAQR7	progestin and adipoQ receptor family member VII [Source:MGI Symbol;Acc:MGI:1919154]	4	134496697	134510235	1	protein_coding	432.8864	-0.570778	0.169593	-3.36558	0.000764	0.032468
ENSMUSG00000035849	KRT222	keratin 222 [Source:MGI Symbol;Acc:MGI:2442728]	11	99232761	99244085	-1	protein_coding	456.383	-0.574483	0.17102	-3.35915	0.000782	0.033049
ENSMUSG00000049537	TECRL	trans-2,3-enoyl-CoA reductase-like [Source:MGI Symbol;Acc:MGI:2444966]	5	83278145	83355195	-1	protein_coding	753.5367	0.48024569	0.142985	3.358723	0.000783	0.033049
ENSMUSG00000068854	HIST2H2BE	histone cluster 2, H2be [Source:MGI Symbol;Acc:MGI:2448415]	3	96221119	96223738	1	protein_coding	261.763	-0.6762315	0.201373	-3.35811	0.000785	0.033049
ENSMUSG00000021799	OPN4	opsin 4 (melanopsin) [Source:MGI Symbol;Acc:MGI:1353425]	14	34590618	34600142	-1	protein_coding	311.694	-0.6724909	0.200291	-3.35756	0.000786	0.033049
ENSMUSG00000030731	SYT3	synaptotagmin III [Source:MGI Symbol;Acc:MGI:99665]	7	44384102	44400187	1	protein_coding	80.43135	-1.1175844	0.33291	-3.35702	0.000788	0.033049
ENSMUSG00000020387	JADE2	jade family PHD finger 2 [Source:MGI Symbol;Acc:MGI:1924151]	11	51813455	51857653	-1	protein_coding	457.617	0.6729658	0.200872	3.350219	0.000807	0.033767
ENSMUSG00000045659	PLEKHA7	pleckstrin homology domain containing, family A member 7 [Source:MGI Symbol;Acc:MGI:2445094]	7	116123493	116308376	-1	protein_coding	1585.259	-0.5332441	0.159195	-3.34962	0.000809	0.033767

ENSMUSG00000079012	SERPINA3M	serine (or cysteine) peptidase inhibitor, clade A, member 3M [Source:MGI Symbol;Acc:MGI:98378]	12	104338486	104394257	1	protein_coding	9.770319	4.19987766	1.254342	3.348271	0.000813	0.033843
ENSMUSG00000028427	AQP7	aquaporin 7 [Source:MGI Symbol;Acc:MGI:1314647]	4	41033074	41048139	-1	protein_coding	406.1652	0.80129327	0.239449	3.346405	0.000819	0.033983
ENSMUSG00000026817	AK1	adenylate kinase 1 [Source:MGI Symbol;Acc:MGI:87977]	2	32621758	32635058	1	protein_coding	7125.748	-0.4107739	0.122935	-3.3414	0.000834	0.034511
ENSMUSG00000059974	NTM	neurotrimin [Source:MGI Symbol;Acc:MGI:2446259]	9	28994750	29963141	-1	protein_coding	78.1102	-1.1294112	0.338302	-3.33847	0.000842	0.034787
ENSMUSG00000040387	KLHL32	kelch-like 32 [Source:MGI Symbol;Acc:MGI:3612790]	4	24612554	24851124	-1	protein_coding	162.9144	-0.8532749	0.255711	-3.33687	0.000847	0.034898
ENSMUSG00000032715	TRIB3	tribbles homolog 3 (Drosophila) [Source:MGI Symbol;Acc:MGI:1345675]	2	152337422	152344032	-1	protein_coding	113.9669	0.93537554	0.280685	3.332477	0.000861	0.035362
ENSMUSG00000027359	SLC27A2	solute carrier family 27 (fatty acid transporter), member 2 [Source:MGI Symbol;Acc:MGI:1347099]	2	126552407	126588243	1	protein_coding	8.265761	4.59500161	1.37923	3.331571	0.000864	0.035386
ENSMUSG00000022546	GPT	glutamic pyruvic transaminase, soluble [Source:MGI Symbol;Acc:MGI:95802]	15	76696764	76699674	1	protein_coding	141.6509	-0.8739678	0.262442	-3.33014	0.000868	0.035478
ENSMUSG00000051550	ZFP579	zinc finger protein 579 [Source:MGI Symbol;Acc:MGI:1915740]	7	4983483	4996158	-1	protein_coding	2504.971	-0.4858899	0.146076	-3.32629	0.00088	0.03588
ENSMUSG00000038375	TRP53INP2	transformation related protein 53 inducible nuclear protein 2 [Source:MGI Symbol;Acc:MGI:1915978]	2	155381059	155389850	1	protein_coding	12032.16	0.39088749	0.117667	3.321979	0.000894	0.036345
ENSMUSG00000032515	CSRNP1	cysteine-serine-rich nuclear protein 1 [Source:MGI Symbol;Acc:MGI:2387989]	9	119971166	119977250	-1	protein_coding	324.4546	0.86837041	0.261675	3.318508	0.000905	0.036706
ENSMUSG00000031489	ADRB3	adrenergic receptor, beta 3 [Source:MGI Symbol;Acc:MGI:87939]	8	27225776	27229588	-1	protein_coding	77.28177	1.66086013	0.500741	3.316805	0.000911	0.036773
ENSMUSG00000058498	RNF207	ring finger protein 207 [Source:MGI Symbol;Acc:MGI:2684989]	4	152307019	152318993	-1	protein_coding	1877.48	-0.5061982	0.152626	-3.31659	0.000911	0.036773
ENSMUSG00000045201	LRRC3B	leucine rich repeat containing 3B [Source:MGI Symbol;Acc:MGI:2384996]	14	15357519	15438987	-1	protein_coding	1036.395	-0.5210069	0.15716	-3.31514	0.000916	0.03687
ENSMUSG00000057337	CHST3	carbohydrate (chondroitin 6/keratan) sulfotransferase 3 [Source:MGI Symbol;Acc:MGI:1858224]	10	60181532	60219260	-1	protein_coding	36.83955	1.63765146	0.494459	3.312009	0.000926	0.037191
ENSMUSG00000018405	MRM1	mitochondrial rRNA methyltransferase 1 homolog (S. cerevisiae) [Source:MGI Symbol;Acc:MGI:2443470]	11	84813061	84819515	-1	protein_coding	599.3934	-0.5284618	0.159677	-3.30957	0.000934	0.037423
ENSMUSG00000092344	HSPE1-PS3	heat shock protein 1 (chaperonin 10), pseudogene 3 [Source:MGI Symbol;Acc:MGI:1935163]	5	25391608	25391884	1	processed_pseudogene	27.6001	1.87248915	0.566109	3.307648	0.000941	0.037559
ENSMUSG00000030852	TACC2	transforming, acidic coiled-coil containing protein 2 [Source:MGI Symbol;Acc:MGI:1928899]	7	130577484	130764784	1	protein_coding	3022.294	0.55121755	0.166675	3.307143	0.000943	0.037559
ENSMUSG00000035021	BAZ1A	bromodomain adjacent to zinc finger domain 1A [Source:MGI Symbol;Acc:MGI:1309478]	12	54892989	55014348	-1	protein_coding	156.2555	0.77726759	0.235076	3.306448	0.000945	0.037559
ENSMUSG00000049303	SYT12	synaptotagmin XII [Source:MGI Symbol;Acc:MGI:2159601]	19	4445908	4477447	-1	protein_coding	93.22858	1.02783014	0.311354	3.301161	0.000963	0.038169
ENSMUSG00000001473	TUBB6	tubulin, beta 6 class V [Source:MGI Symbol;Acc:MGI:1915201]	18	67390717	67402748	1	protein_coding	545.2531	0.74017835	0.224271	3.300368	0.000966	0.038169
ENSMUSG00000034771	TLE2	transducin-like enhancer of split 2, homolog of Drosophila E(spl) [Source:MGI Symbol;Acc:MGI:104635]	10	81574561	81590845	1	protein_coding	334.4021	-0.6937779	0.210246	-3.29984	0.000967	0.038169
ENSMUSG00000035681	KCNC2	potassium voltage gated channel, Shaw-related subfamily, member 2 [Source:MGI Symbol;Acc:MGI:96668]	10	112271123	112466304	1	protein_coding	82.90627	1.15302452	0.349644	3.297713	0.000975	0.038364
ENSMUSG00000030513	PCSK6	proprotein convertase subtilisin/kexin type 6 [Source:MGI Symbol;Acc:MGI:102897]	7	65861734	66050340	1	protein_coding	1083.311	-0.4696401	0.142585	-3.29376	0.000989	0.038812
ENSMUSG00000078866	GM14420	predicted gene 14420 [Source:MGI Symbol;Acc:MGI:3652255]	2	177464733	177478129	1	protein_coding	243.8264	0.74167176	0.225461	3.289574	0.001003	0.039297
ENSMUSG00000040423	RC3H1	RING CCCH (C3H) domains 1 [Source:MGI Symbol;Acc:MGI:2685397]	1	160906418	160974978	1	protein_coding	951.5427	0.62720474	0.190953	3.28461	0.001021	0.039898
ENSMUSG00000027082	TFPI	tissue factor pathway inhibitor [Source:MGI Symbol;Acc:MGI:1095418]	2	84432855	84476775	-1	protein_coding	1935.689	-0.4388183	0.133633	-3.28375	0.001024	0.039922
ENSMUSG00000030168	ADIPOR2	adiponectin receptor 2 [Source:MGI Symbol;Acc:MGI:93830]	6	119353150	119417704	-1	protein_coding	1816.87	0.42936411	0.130816	3.282209	0.00103	0.040043
ENSMUSG00000026414	TNNT2	troponin T2, cardiac [Source:MGI Symbol;Acc:MGI:104597]	1	135836354	135852260	1	protein_coding	99608.23	-0.428645	0.130666	-3.28047	0.001036	0.040193
ENSMUSG00000031960	AARS	alanyl-tRNA synthetase [Source:MGI Symbol;Acc:MGI:2384560]	8	111033144	111057664	1	protein_coding	1619.04	0.4678313	0.142648	3.279613	0.00104	0.040217
ENSMUSG00000018740	SLC25A35	solute carrier family 25, member 35 [Source:MGI Symbol;Acc:MGI:1919248]	11	68968131	68974365	1	protein_coding	130.8309	-0.8664934	0.264787	-3.27242	0.001066	0.041071
ENSMUSG00000041782	LAD1	ladinin [Source:MGI Symbol;Acc:MGI:109343]	1	135818598	135833342	1	protein_coding	616.1427	-0.6544874	0.200007	-3.27232	0.001067	0.041071
ENSMUSG00000037434	SLC30A1	solute carrier family 30 (zinc transporter), member 1 [Source:MGI Symbol;Acc:MGI:1345281]	1	191906767	191913247	1	protein_coding	273.0815	0.63483051	0.194075	3.271064	0.001071	0.041153
ENSMUSG00000042190	CMKLR1	chemokine-like receptor 1 [Source:MGI Symbol;Acc:MGI:109603]	5	113612354	113650426	-1	protein_coding	394.3193	0.71900774	0.219892	3.269815	0.001076	0.041183
ENSMUSG00000023886	SMOC2	SPARC related modular calcium binding 2 [Source:MGI Symbol;Acc:MGI:1929881]	17	14279506	14404790	1	protein_coding	573.5246	-0.5139865	0.157227	-3.26907	0.001079	0.041183
ENSMUSG00000005087	CD44	CD44 antigen [Source:MGI Symbol;Acc:MGI:88338]	2	102811141	102901665	-1	protein_coding	533.4283	0.57373131	0.175516	3.268823	0.00108	0.041183
ENSMUSG00000002504	SLC9A3R2	solute carrier family 9 (sodium/hydrogen exchanger), member 3 regulator 2 [Source:MGI Symbol;Acc:MGI:1890662]	17	24639282	24650305	-1	protein_coding	1398.184	0.56063426	0.171576	3.26756	0.001085	0.041269
ENSMUSG00000022309	ANGPT1	angiopoietin 1 [Source:MGI Symbol;Acc:MGI:108448]	15	42424727	42676977	-1	protein_coding	2559.607	-0.6507025	0.199206	-3.26649	0.001089	0.041327
ENSMUSG00000001349	CNN1	calponin 1 [Source:MGI Symbol;Acc:MGI:104979]	9	22099281	22109630	1	protein_coding	150.8241	1.08850645	0.333642	3.262501	0.001104	0.041813
ENSMUSG00000026418	TNNI1	troponin I, skeletal, slow 1 [Source:MGI Symbol;Acc:MGI:105073]	1	135779434	135810989	1	protein_coding	44.39199	-1.3881835	0.425873	-3.25962	0.001116	0.04214
ENSMUSG00000024525	IMPA2	inositol (myo)-1(or 4)-monophosphatase 2 [Source:MGI Symbol;Acc:MGI:2149728]	18	67289253	67319146	1	protein_coding	164.8603	0.87948425	0.270115	3.255966	0.00113	0.042524
ENSMUSG00000032717	MDFI	MyoD family inhibitor [Source:MGI Symbol;Acc:MGI:107687]	17	47815328	47834691	-1	protein_coding	214.2216	-0.7485984	0.229935	-3.2557	0.001131	0.042524
ENSMUSG00000002032	TMEM25	transmembrane protein 25 [Source:MGI Symbol;Acc:MGI:1918937]	9	44793776	44799307	-1	protein_coding	102.4984	-0.9923105	0.305153	-3.25184	0.001147	0.043004
ENSMUSG00000024869	NUDT8	nudix (nucleoside diphosphate linked moiety X)-type motif 8 [Source:MGI Symbol;Acc:MGI:1913637]	19	4000580	4005816	1	protein_coding	1700.846	-0.4164647	0.128143	-3.25001	0.001154	0.043181
ENSMUSG00000020250	TXNRD1	thioredoxin reductase 1 [Source:MGI Symbol;Acc:MGI:1354175]	10	82859206	82897724	1	protein_coding	1432.946	0.47392839	0.145857	3.249277	0.001157	0.043191
ENSMUSG00000061815	RUFY4	RUN and FYVE domain containing 4 [Source:MGI Symbol;Acc:MGI:3588214]	1	74125541	74148223	1	protein_coding	109.1562	0.95904293	0.295399	3.246606	0.001168	0.043496
ENSMUSG00000024331	DSC2	desmocollin 2 [Source:MGI Symbol;Acc:MGI:103221]	18	20030633	20059554	-1	protein_coding	1983.891	-0.5145184	0.158589	-3.24435	0.001177	0.04374
ENSMUSG00000021871	PNP	purine-nucleoside phosphorylase [Source:MGI Symbol;Acc:MGI:97365]	14	50944302	50965237	1	protein_coding	566.8785	0.5397625	0.166659	3.238724	0.001201	0.044508
ENSMUSG00000050666	VSTM4	V-set and transmembrane domain containing 4 [Source:MGI Symbol;Acc:MGI:2444633]	14	32856756	32939487	1	protein_coding	318.1208	-0.5968703	0.184408	-3.23668	0.001209	0.044725
ENSMUSG00000036752	TUBB4B	tubulin, beta 4B class IVB [Source:MGI Symbol;Acc:MGI:1915472]	2	25222160	25224702	-1	protein_coding	4657.025	0.50188808	0.155552	3.226505	0.001253	0.046238
ENSMUSG00000022665	CCDC80	coiled-coil domain containing 80 [Source:MGI Symbol;Acc:MGI:1915146]	16	45093402	45127924	1	protein_coding	5438.048	0.45267664	0.140427	3.22358	0.001266	0.046442
ENSMUSG00000031530	DUSP4	dual specificity phosphatase 4 [Source:MGI Symbol;Acc:MGI:2442191]	8	34807297	34819894	1	protein_coding	56.5403	1.47299187	0.456962	3.223443	0.001267	0.046442
ENSMUSG00000046982	TSHZ1	teashirt zinc finger family member 1 [Source:MGI Symbol;Acc:MGI:1346031]	18	84011627	84086404	-1	protein_coding	1114.045	0.52304736	0.162273	3.223265	0.001267	0.046442
ENSMUSG00000100658	F730311O21RIK	RIKEN cDNA F730311O21 gene [Source:MGI Symbol;Acc:MGI:3643355]	1	132342023	132349626	1	lincRNA	32.23496	1.63539481	0.508276	3.217536	0.001293	0.04727
ENSMUSG00000078937	CPT1B	carnitine palmitoyltransferase 1b, muscle [Source:MGI Symbol;Acc:MGI:1098297]	15	89416405	89425863	-1	protein_coding	10358.31	0.37021043	0.115156	3.214868	0.001305	0.047565
ENSMUSG00000067780	PI15	peptidase inhibitor 15 [Source:MGI Symbol;Acc:MGI:1934659]	1	17601901	17630939	1	protein_coding	207.6018	0.96731879	0.300929	3.214439	0.001307	0.047565
ENSMUSG00000020593	LPIN1	lipin 1 [Source:MGI Symbol;Acc:MGI:1891340]	12	16535669	16589770	-1	protein_coding	3638.814	0.43396882	0.135109	3.211985	0.001318	0.047864
ENSMUSG00000072675	DUXBL2	doubl homeobox B-like 2 [Source:MGI Symbol;Acc:MGI:3710620]	14	26118349	26129763	1	protein_coding	7.191287	-5.2265359	1.629137	-3.20816	0.001336	0.048395
ENSMUSG00000064368	MT-ND6	mitochondrially encoded NADH dehydrogenase 6 [Source:MGI Symbol;Acc:MGI:102495]	MT	13552	14070	-1	protein_coding	84450.83	-0.3876156	0.120859	-3.20717	0.001341	0.048453
ENSMUSG00000075232	AMD1	S-adenosylmethionine decarboxylase 1 [Source:MGI Symbol;Acc:MGI:88004]	10	40287458	40302188	-1	protein_coding	3474.168	0.40461644	0.126283	3.204036	0.001355	0.048871
ENSMUSG00000059923	GRB2	growth factor receptor bound protein 2 [Source:MGI Symbol;Acc:MGI:95805]	11	115644045	115708597	-1	protein_coding	1885.065	-0.4124877	0.128827	-3.20188	0.001365	0.049128
ENSMUSG00000033685	UCP2	uncoupling protein 2 (mitochondrial, proton carrier) [Source:MGI Symbol;Acc:MGI:109354]	7	100493337	100502020	1	protein_coding	12340.5	0.47293407	0.14789	3.197872	0.001384	0.049703

Supplemental Table 2. Significantly (FWER-p <0.05) enriched KEGG cellular processes of downregulated genes in obese versus lean mouse atria.

Pathway	P_{adj}	NES	Leading Edge Genes
KEGG_OXIDATIVE_PHOSPHORYLATION	0	-5.490638	*COX4I1, NDUFA2, ATP5F1, NDUFB4, COX8A, UQCRQ, ATP5D, NDUFS7, UQCRFS1, ATP6V1H, NDUFC2, ATP5J2, NDUFB9, NDUFA1, NDUFB6, NDUFA3, NDUFB8, SDHC, COX7A2, NDUFS4, NDUFB5, NDUFB2, COX4I2, NDUFA8, NDUFA4L2, COX10, NDUFA7, ATP6V1E2, UQCR11, CYC1, ATP5B, NDUFS2, ATP5E, NDUFB7, NDUFA11, ATP5G3, ATP6V1D, COX5A, ATP5A1, NDUFS8, ATP5G1, NDUFS3, SDHB, ATP6V0E2, NDUFA6, UQCRC2, COX5B, SDHD, COX6B1, UQCRH, COX7A2L, NDUFC1, COX17, NDUFA10, COX6C, SDHA, PPA2, UQCRC1, NDUFA9, NDUFV1, NDUFB3, NDUFV3, COX15, ATP5O, LHPP, ATP5C1, ATP6V1G1, NDUFS1, COX6A2, NDUFV2, ATP5H, UQCRB, NDUFA4, COX7A1, COX7B, ATP6V1E1, COX7C*
KEGG_PARKINSONS_DISEASE	0	-5.0884438	*COX4I1, NDUFA2, ATP5F1, NDUFB4, COX8A, UQCRQ, PARK7, ATP5D, NDUFS7, UQCRFS1, NDUFC2, NDUFB9, NDUFA1, NDUFB6, VDAC3, NDUFA3, CASP3, NDUFB8, SDHC, PINK1, COX7A2, NDUFS4, NDUFB5, NDUFB2, COX4I2, NDUFA8, NDUFA4L2, NDUFA7, UQCR11, CYC1, ATP5B, NDUFS2, ATP5E, SLC18A2, NDUFB7, ATP5G3, COX5A, ATP5A1, NDUFS8, ATP5G1, NDUFS3, SDHB, NDUFA6, UQCRC2, COX5B, SDHD, COX6B1, UQCRH, COX7A2L, NDUFC1, NDUFA10, VDAC1, SNCAIP, COX6C, SDHA, UQCRC1, NDUFA9, VDAC2, NDUFV1, NDUFB3, NDUFV3, ATP5O, ATP5C1, NDUFS1, COX6A2, NDUFV2, PARK2, HTRA2, ATP5H, UQCRB, NDUFA4, COX7A1, COX7B, COX7C*
KEGG_HUNTINGTONS_DISEASE	0	-4.163713	*COX4I1, NDUFA2, ATP5F1, DNALI1, NDUFB4, COX8A, UQCRQ, ATP5D, NDUFS7, AP2S1, SOD2, UQCRFS1, NDUFC2, TBPL1, POLR2H, NDUFB9, NDUFA1, NDUFB6, VDAC3, NDUFA3, CASP3, NDUFB8, SDHC, COX7A2, NDUFS4, NDUFB5, NDUFB2, COX4I2, NDUFA8, NDUFA4L2, AP2A1, DCTN2, NDUFA7, IFT57, DNAH1, UQCR11, POLR2G, CYC1, HDAC1, ATP5B, NDUFS2, ATP5E, TFAM, NDUFB7, ATP5G3, CREB3L2, POLR2J, COX5A, ATP5A1, NDUFS8, ATP5G1, NDUFS3, SDHB, NDUFA6, UQCRC2, DNAL4, COX5B, SDHD, COX6B1, UQCRH, POLR2L, COX7A2L, NDUFC1, POLR2D, NDUFA10, VDAC1, COX6C, SDHA, UQCRC1, NDUFA9, VDAC2, NDUFV1, NDUFB3, NDUFV3, HDAC2, ATP5O, ATP5C1, NDUFS1, COX6A2, NDUFV2, ATP5H, UQCRB, NDUFA4, COX7A1, COX7B, COX7C*
KEGG_ALZHEIMERS_DISEASE	0	-4.145941	*ITPR3, COX4I1, NDUFA2, ATP5F1, NDUFB4, COX8A, UQCRQ, ATP5D, NDUFS7, UQCRFS1, NDUFC2, NDUFB9, NDUFA1, NDUFB6, NDUFA3, CASP3, APH1A, NDUFB8, SDHC, CALM1, COX7A2, NDUFS4, NDUFB5, NDUFB2, COX4I2, NDUFA8, NDUFA4L2, NDUFA7, PPP3R1, UQCR11, CYC1, ATP5B, NDUFS2, ATP5E, NDUFB7, ATP5G3, COX5A, ATP5A1, NDUFS8, PSENEN, NCSTN, ATP5G1, NDUFS3, SDHB, NDUFA6, GAPDH, UQCRC2, COX5B, SDHD, COX6B1, UQCRH, CASP7, COX7A2L, NDUFC1, NDUFA10, COX6C, SDHA, UQCRC1, NDUFA9, FADD, NDUFV1, HSD17B10, NDUFB3, NDUFV3, ATP5O, GRIN2C, ATP5C1, NDUFS1, COX6A2, NDUFV2, CALM2, ATP5H, CALM3, MME, UQCRB, NDUFA4, COX7A1, COX7B, CACNA1D, APOE, BACE2, COX7C*
KEGG_VALINE_LEUCINE_AND_ISOLEUCINE_DEGRADATION	0	-3.6559122	*ACADSB, PCCA, MUT, HIBADH, AUH, HIBCH, HADH, ALDH2, HMGCL, MCEE, IL4I1, HSD17B10, BCAT2, ACAD8, BCKDHB, ALDH7A1, DBT, ACAT1, OXCT1, BCKDHA, MCCC2, ECHS1, MCCC1, PCCB, ABAT, IVD, ALDH1B1*
KEGG_CITRATE_CYCLE_TCA_CYCLE	0	-2.920196	*ACLY, ACO1, IDH3A, DLD, OGDH, MDH1, DLAT, PDHA1, SDHC, SUCLG1, MDH2, IDH3B, CS, SDHB, SDHD, ACO2, IDH3G, SDHA, SUCLA2, DLST, SUCLG2, PDHB, IDH2*
KEGG_CARDIAC_MUSCLE_CONTRACTION	0	-2.856165	*CACNG7, ATP1B4, COX7B2, CACNB4, CACNA2D3, COX4I1, TNNC1, COX8A, UQCRQ, UQCRFS1, CACNA2D1, CACNB2, CACNG6, COX7A2, COX4I2, UQCR11, MYL2, CYC1, MYH6, COX5A, MYL3, UQCRC2, COX5B, COX6B1, UQCRH, COX7A2L, TPM1, COX6C, UQCRC1, COX6A2, CACNB1, UQCRB, COX7A1, COX7B, CACNA1D, MYH7, TNNI3, TNNT2, ATP1B2, CACNA2D2, COX7C*
KEGG_PYRUVATE_METABOLISM	0	-2.597899	*MDH1, DLAT, PDHA1, HAGH, GLO1, ME2, MDH2, ACYP1, LDHB, ALDH2, GRHPR, ACYP2, ACSS2, LDHD, ALDH7A1, ME3, ACAT1, ACACA, PDHB, HAGHL, ALDH1B1*
KEGG_PROPANOATE_METABOLISM	0	-2.5466135	*PCCA, MUT, SUCLG1, HIBCH, LDHB, ALDH2, MCEE, SUCLA2, ACSS2, ALDH7A1, ACAT1, SUCLG2, ACACA, ECHS1, PCCB, ABAT, ALDH1B1*
KEGG_RIBOSOME	0	-2.4936714	*RPL10A, RPL18, RPS27L, RPL22L1, RPS6, RPLP0, RPL3, RPL15, RPS9, RSL24D1, RPS23, RPS29, RPS18, RPS8, RPS5, RPL37A, RPL8, RPL23, RPL35A, RPL6, RPS26, RPL13, RPL39, RPL24, RPL7, RPL19, RPL32, RPS20, RPL17, RPS25, RPL36AL, RPS11, RPL36, RPS15, RPL31, RPL26, RPL13A, RPL4, RPL34, RPL9, RPL7A, RPL41, RPS10, RPS16, RPS17, RPL14, RPL21, RPL36A, UBA52, RPS21, RPS27, RPS4X, RPS27A, RPS24, RPL5, RPL38, RPS13, RPL35, RPL11, RPL22, MRPL13, RPS7, RPL23A, RPL30*
KEGG_BUTANOATE_METABOLISM	0	-2.1046991	*HADH, ALDH2, HMGCL, AKR1B10, ALDH7A1, BDH1, ACAT1, PDHB, OXCT1, ECHS1, ABAT, ALDH1B1*
KEGG_GLYOXYLATE_AND_DICARBOXYLATE_METABOLISM	0.00967118	-2.0233512	*MTHFD1, MTHFD1L, PGP, ACO1, GLYCTK, MDH1, HYI, MDH2, CS, ACO2, GRHPR, AFMID*
KEGG_METABOLISM_OF_XENOBIOTICS_BY_CYTOCHROME_P450	0.00391389	-1.9639288	*GSTO2, GSTO1, GSTM2, GSTA2, GSTM1, GSTA1, GSTP1, GSTA3, GSTZ1, GSTA4, GSTM4, ADH5, EPHX1, GSTK1, GSTM5, GSTT2, ALDH3B2, GSTT1*
KEGG_PYRIMIDINE_METABOLISM	0.00801603	-1.8938818	*POLR1A, TK2, NT5E, RRM2, RRM2B, NT5C2, POLR2K, TYMS, NME7, POLE3, UCK1, UMPS, NME5, POLR2E, POLR2B, ZNRD1, PRIM2, POLR3GL, DPYD, POLR2I, POLR2C, CTPS2, NME1, NME6, TXNRD2, RRM1, PNPT1, UPB1, POLR2H, DCTD, NT5M, POLR3B, POLE, ENTPD3, POLA2, PRIM1, DCK, POLR2G, POLR3K, AK3, NT5C, NUDT2, POLR2J, TYMP, NT5C3, DTYMK, UCKL1, POLD2, POLR2L, POLR3G, POLR2D, POLA1, POLE4, POLR1E, ENTPD5, NME4, DUT, NME2, DHODH*
KEGG_NUCLEOTIDE_EXCISION_REPAIR	0.00809717	-1.8762724	*ERCC8, GTF2H3, PCNA, ERCC4, RPA1, RPA2, RAD23A, GTF2H1, RFC2, POLE, ERCC2, MNAT1, ERCC3, POLD2, RFC3, DDB2, RFC5, CETN2, GTF2H2, POLE4, CCNH, RBX1, GTF2H5, CDK7*
KEGG_PORPHYRIN_AND_CHLOROPHYLL_METABOLISM	0.00787402	-1.8569026	*ALAS1, HMBS, BLVRA, UROD, BLVRB, PPOX, COX10, EARS2, FECH, ALAD, COX15, HMOX2, MMAB*
KEGG_GLUTATHIONE_METABOLISM	0.0417495	-1.6805501	*GSTO2, GSTO1, RRM1, ANPEP, GSTM2, GSTA2, LAP3, GSTM1, GSTA1, GSTP1, GSTA3, GSTZ1, GGT1, GSTA4, GSTM4, GSTK1, GGT7, GSTM5, GGCT, IDH2, GSTT2, GSTT1*
KEGG_PEROXISOME	0.03541667	-1.673484	*MVK, DECR2, PEX11G, PRDX5, HMGCL, ABCD1, HACL1, PMVK, PEX19, PIPOX, DDO, NUDT19, GSTK1, PXMP2, ACSL6, PEX2, PECR, PHYH, NOS2, IDH2*

KEGG_ALANINE_ASPARTATE_AND_GLUTAMATE_METABOLISM	0.05555556	-1.6059155	*IL4I1, ASL, ADSSL1, DDO, ALDH4A1, GOT2, GPT, ABAT, ACY3*
KEGG_DRUG_METABOLISM_CYTOCHROME_P450	0.05660377	-1.5764015	*GSTO2, GSTO1, GSTM2, GSTA2, GSTM1, GSTA1, MAOB, GSTP1, GSTA3, GSTZ1, GSTA4, GSTM4, ADH5, GSTK1, GSTM5, GSTT2, ALDH3B2, GSTT1*

P_{adj} values are adjusted for multiple testing. NES, nominal enrichment score.

Supplemental Table 3. Significantly (FWER-p <0.05) enriched KEGG cellular processes of upregulated genes in obese versus lean mouse atria.

**Pathway**	**P_{adj}**	NES	**Leading Edge Genes**
KEGG_PATHWAYS_IN_CANCER	0	3.922683	*CDKN1A, WNT9B, PTGS2, COL4A1, FAS, FGFR3, EGLN3, ZBTB16, COL4A2, EGLN1, CBLB, STAT3, LAMC1, HSP90AA1, HSP90B1, CCDC6, TGFBR2, CHUK, NFKBIA, FGF23, HSP90AB1, MAX, FZD1, SMAD4, FGF10, E2F3, LAMC2, NCOA4, PIK3R5, CDK6, PIK3CD, STK36, TGFB3, PPARG, RASSF1, BCR, ITGA2B, AXIN2, FZD9, PGF, WNT4, MYC, SOS2, ITGB1, WNT9A, AKT3, NRAS, DVL1, BIRC2, CSF3R, TGFB2, IGF1R, FZD4, FZD7, LAMC3, BCL2L1, HIF1A, SMAD3, PIK3CG, FGF1, NFKB2, GLI2, CTBP2, DVL2, IKBKB, TRAF6, XIAP, PIK3CB, CASP8, VEGFA, BIRC3, RASSF5, RXRB, JUN, CRK, SPI1, E2F1, KRAS, TRAF2, CREBBP, CEBPA, CRKL, FGF21, GSK3B, PML, MDM2, EPAS1, RUNX1, FGF2, WNT2, DVL3, WNT2B, PTEN, EP300, PDGFB, CDKN1B, PLD1, FN1, PIK3CA, RET, RXRA, RARA, FIGF, ARNT, PTCH1, DAPK2, IKBKG, PLCG1, ITGAV, LAMA5, RAC2, FGFR1, RAD51, DAPK1, BIRC5, CYCS*
KEGG_PPAR_SIGNALING_PATHWAY	0	3.7852383	*ANGPTL4, CD36, CPT1A, FABP4, ACSL1, HMGCS2, CPT2, UCP1, AQP7, SLC27A2, CPT1B, ACADL, ACOX1, PCK1, PDPK1, DBI, FABP3, LPL, PPARG, NR1H3, OLR1, SORBS1, CYP8B1, EHHADH, SLC27A1, ACSL3, PCK2, ME1, RXRB, SLC27A6, SLC27A, RXRA, ACSL5, PLTP, ACOX3, ADIPOQ*
KEGG_FOCAL_ADHESION	**0**	3.5590246	*ACTN1, COL4A1, ITGB3, RAPGEF1, COL4A2, COL5A2, ITGA10, VWF, CAPN2, LAMC1, FLT1, PDGFC, PDPK1, ROCK2, ITGA1, ITGA9, FLT4, FLNB, FLNA, LAMC2, ITGB5, PIK3R5, PIK3CD, PAK2, MYLK, ITGA2B, THBS3, COL11A2, PGF, CCND2, SOS2, ITGB1, AKT3, BIRC2, IGF1R, LAMC3, PIK3CG, MYLK2, XIAP, PIK3CB, VAV1, VEGFA, BIRC3, JUN, ITGA5, CRK, ARHGAP5, COL1A1, CRKL, TNR, ITGB4, PAK3, GSK3B, FLNC, MYL9, PARVA, PTEN, PDGFB, ITGA8, FN1, PIK3CA, PPP1CC, FIGF, KDR, ACTN4, ITGA7, PARVG, TNXB, ITGAV, LAMA5, TLN1, RAC2, COL3A1, CAV2, LAMB2, COL5A1, PPP1R12A, COL11A1, PAK7, ZYX, COL6A3, PAK4, THBS1, RAP1B, COL5A3, ACTN2, CCND1*
KEGG_COMPLEMENT_AND_COAGULATION_CASCADES	0	3.4308343	*C4B, SERPINE1, C3, PLAUR, F13A1, C5AR1, PLAT, VWF, THBD, C1QB, C1QC, CFB, C1QA, PROS1, C6, C3AR1, C7, CD55, MASP1, SERPING1, BDKRB2, SERPIND1, F2R, CFH, F8, F10, F5*
KEGG_REGULATION_OF_ACTIN_CYTOSKELETON	*0*	*3.1357987*	*ITGAM, ACTN1, ITGB3, GNA12, ITGB2, FGFR3, MSN, ITGA10, ARHGEF12, NCKAP1L, ARHGEF6, ITGAL, PDGFC, ROCK2, FGF23, IQGAP1, ITGA1, ITGA9, ARPC1B, CD14, FGF10, ITGB5, PIK3R5, PIK3CD, PAK2, FGD3, ARHGEF7, MYLK, ITGA2B, SOS2, ITGB1, MYH9, NRAS, SSH3, PIK3CG, FGF1, MYLK2, BDKRB2, PIK3CB, VAV1, WASL, ARPC5, LIMK1, ITGA5, CRK, KRAS, SSH1, ENAH, CRKL, FGF21, ITGB4, PAK3, CYFIP1, MYL9, F2R, NCKAP1, ARHGEF1, FGF2, PDGFB, ITGA8, MYH14, CSK, FN1, PIK3CA, GNG12, PPP1CC, ITGAE, IQGAP2, BAIAP2, ACTN4, ITGA7, ITGAV, RAC2, FGFR1, GIT1*
KEGG_SMALL_CELL_LUNG_CANCER	0	3.123023	*PTGS2, COL4A1, COL4A2, LAMC1, CHUK, NFKBIA, MAX, APAF1, E2F3, LAMC2, PIK3R5, CDK6, PIK3CD, ITGA2B, MYC, ITGB1, AKT3, BIRC2, LAMC3, BCL2L1, PIK3CG, IKBKB, TRAF6, XIAP, PIK3CB, BIRC3, RXRB, E2F1, TRAF2, PTEN, CDKN1B, FN1, PIK3CA, RXRA, IKBKG, ITGAV, LAMA5, CYCS, TRAF5, LAMB2*
KEGG_CHRONIC_MYELOID_LEUKEMIA	0	3.0990667	*CDKN1A, PTPN11, CBLB, TGFBR2, GAB2, CHUK, NFKBIA, SMAD4, E2F3, PIK3R5, CDK6, PIK3CD, TGFB3, BCR, MYC, SOS2, AKT3, NRAS, TGFB2, BCL2L1, SMAD3, PIK3CG, CTBP2, IKBKB, PIK3CB, CRK, E2F1, KRAS, CRKL, MDM2, RUNX1*
KEGG_ECM_RECEPTOR_INTERACTION	0	2.9438941	*CD36, COL4A1, ITGB3, CD44, COL4A2, COL5A2, ITGA10, VWF, LAMC1, ITGA1, ITGA9, LAMC2, ITGB5, DAG1, SDC4, ITGA2B, THBS3, COL11A2, ITGB1, CD47, LAMC3, ITGA5, COL1A1, TNR, ITGB4, HSPG2, AGRN, ITGA8, FN1, ITGA7, TNXB, ITGAV, LAMA5, COL3A1, LAMB2, COL5A1, COL11A1, GP5, COL6A3, THBS1, COL5A3*
KEGG_CHEMOKINE_SIGNALING_PATHWAY	**0**	2.7417037	*GRK5, CCL8, ADCY6, ADCY4, PTK2B, RASGRP2, GNGT2, STAT3, PLCB4, ARRB1, CCR1, ROCK2, CHUK, NFKBIA, JAK3, CXCL12, FOXO3, CXCL13, PIK3R5, PIK3CD, GNG4, ADRBK2, SOS2, PPBP, NCF1, CCL5, AKT3, NRAS, PREX1, CXCL10, PIK3CG, PARD3, PRKX, IKBKB, STAT2, PIK3CB, VAV1, CCR7, GNG13, WASL, CRK, DOCK2, KRAS, ADCY3, ADCY5, CCR5, CRKL, GSK3B, XCR1, CXCL16, CCR2, CX3CL1, ADRBK1, CSK, ADCY7, PIK3CA, GNG12*
KEGG_CYTOKINE_CYTOKINE_RECEPTOR_INTERACTION	0	2.7333863	*INHBB, CCL8, FAS, OSMR, CSF2RB, INHBA, FLT1, IL2RG, EDAR, TGFBR2, IL18RAP, CCR1, PDGFC, CXCL12, TNFRSF1B, CXCL13, IL11, FLT4, TNFRSF1A, IL1R1, TGFB3, IL7R, IL17RA, IL1B, PPBP, CCL5, CSF3R, TGFB2, CXCL10, IL10RA, IL6ST, BMPR1B, IL18R1, CCR7, VEGFA, TNFRSF12A, BMPR1A, TNFSF14, CCR5, ACVRL1, IL21R, XCR1, IFNGR1, EPOR, IL12A, CXCL16, CCR2, CX3CL1, OSM, PDGFB, TNFRSF19, IL12RB1, RELT, TNFRSF11A, FIGF, KDR, BMP7, PLEKHO2, IL20RA, IL9R, IL4, TNFSF18, CCL2, IL17RB, ACVR2B, IL22RA2, TGFBR1, CCR3, CD40, CD40LG, CCL3, PF4, IL2, TNFSF8, MPL, CXCR3, IL3RA, IL7, IL23R, FLT3, CXCL9, EGFR, LTB, CCL25, IL12B, HGF, CCL19*
KEGG_LYSOSOME	0	2.6376307	*CTSS, SCARB2, NPC1, LAPTM5, CTSC, ATP6V0A4, AP3D1, IDUA, ATP6V0D1, SLC11A1, ACP5, CD68, CLTC, GBA, NAPSA, ATP6V0A2, AP3S1, NAGPA, LAMP1, CTSD, CTSH, CLTB, ACP2, CD63, CTSB, HYAL1, AP1S3, ATP6V0A1, TCIRG1, GALC, AP4E1, PSAP, IGF2R, HEXA, GNS, ABCA2, AP1B1, LIPA, LAMP2, ARSA, ATP6V0D2, CTNS, CLN5, AP1S1, CTSZ, GUSB, PLA2G15, CLN3, AP1G1, AP1M1, GGA1, CTSO, NPC2, ATP6V0B, AP3B1, AP4M1, ATP6AP1, GAA, IDS, GNPTAB, SLC11A2, CTSA, SMPD1, GGA3, CLTA, MAN2B1, AP1M2, DNASE2B, LAMP3*
KEGG_APOPTOSIS	0	2.6122968	*PRKAR2B, FAS, CSF2RB, CAPN2, IRAK3, RIPK1, CHUK, NFKBIA, APAF1, TNFRSF1A, IL1R1, PIK3R5, PIK3CD, IL1B, AKT3, BIRC2, BCL2L1, PIK3CG, PRKX, IKBKB, XIAP, PIK3CB, CASP8, BIRC3, TRAF2, MYD88, IRAK2, PIK3CA, IKBKG, PRKAR2A, DFFA, IRAK4, CYCS, CFLAR, NGF*
KEGG_RENAL_CELL_CARCINOMA	0	2.6106722	*PTPN11, EGLN3, RAPGEF1, EGLN1, PIK3R5, PIK3CD, TGFB3, PAK2, PGF, SOS2, AKT3, FLCN, NRAS, TGFB2, HIF1A, PIK3CG, PIK3CB, VEGFA, JUN, CRK, KRAS, CREBBP, CRKL, PAK3, EPAS1, EP300, PDGFB, PIK3CA, FIGF, ARNT*
KEGG_RIG_I_LIKE_RECEPTOR_SIGNALING_PATHWAY	0	2.4787922	*TRIM25, RIPK1, CHUK, NFKBIA, TBK1, ISG15, IRF7, DDX58, AZI2, CXCL10, IKBKB, TRAF6, CYLD, CASP8, DDX3Y, TRAF2, DDX3X, MAPK14, IL12A, RNF125, TMEM173, MAP3K1, IKBKG, ATG12, MAVS, IL12B, MAPK11, OTUD5, MAPK10, DAK, TANK, DHX58*
KEGG_JAK_STAT_SIGNALING_PATHWAY	**0**	2.4483607	*PTPN11, SOCS3, OSMR, CSF2RB, CBLB, STAT3, IL2RG, SOCS7, PIM1, JAK3, IL11, PIK3R5, PIK3CD, IL7R, MYC, CCND2, SOS2, AKT3, CSF3R, IL10RA, BCL2L1, IL6ST, PIK3CG, STAT4, STAT2, PIK3CB, IL13RA2, CREBBP, IL21R, IFNGR1, EPOR, IL12A, STAM, EP300, OSM, PIK3CA, IL12RB1, SOCS4, IL20RA, IL9R, IL4, CISH, SPRY4, IL22RA2, CCND1, JAK1, IL2, MPL, IL3RA, IL7, IL23R, SOCS5, STAT6, IL12B, STAT1, JAK2, PRLR, IL22RA1, PIK3R1, IL12RB2, PIAS1, IFNAR1, CCND3, PTPN6, IL10, TPO*

KEGG_VIRAL_MYOCARDITIS	0	2.4342818	*ITGB2, ITGAL, DAG1, ICAM1, SGCG, MYH11, CD55, MYH9, EIF4G1, EIF4G2, CASP8, PRF1, MYH13, MYH14, CD80, ABL2, DMD, RAC2, SGCD, CYCS, BID, CD40, CD40LG, CD28, CCND1, ACTG1, MYH3*
KEGG_CIRCADIAN_RHYTHM_MAMMAL	0	2.4189248	*ARNTL, NPAS2, CLOCK, BHLHE40, CSNK1E, PER1, CRY1, PER2, CRY2, NR1D1*
KEGG_INSULIN_SIGNALING_PATHWAY	**0**	2.4168446	*LIPE, PRKAR2B, SOCS3, RAPGEF1, FBP2, IRS2, CBLB, ACACB, PHKA1, PCK1, PDPK1, PPARGC1A, PTPRF, INSR, PIK3R5, PIK3CD, GCK, PDE3B, HK3, MKNK2, SOS2, AKT3, NRAS, PPP1R3A, SORBS1, INPP5D, TSC1, IRS1, PIK3CG, PTPN1, PRKX, IKBKB, PIK3CB, PCK2, CRK, KRAS, CRKL, GSK3B*
KEGG_FATTY_ACID_METABOLISM	0.00210526	2.4163308	*CPT1A, ALDH9A1, ACSL1, HADHA, CPT2, ACADVL, CPT1B, ACADL, ACOX1, ECI2, ACAA2, HADHB, ECI1, EHHADH, ACSL3, ALDH3A2, ADH7, ACSL5, ACOX3*
KEGG_ADIPOCYTOKINE_SIGNALING_PATHWAY	0.002079	2.4028049	*CD36, CPT1A, PTPN11, ACSL1, SOCS3, ADIPOR2, CPT1B, IRS2, STAT3, ACACB, PCK1, PPARGC1A, CHUK, NFKBIA, TNFRSF1B, TNFRSF1A, AKT3, IRS1, IKBKB, ACSL3, PCK2, RXRB, TRAF2*

P_{adj} values are adjusted for multiple testing. NES, nominal enrichment score.

Supplemental Table 4. Cardiac electrophysiological parameters determined with in vivo EP study in obese NLRP3$^{-/-}$ mice

	NLRP3$^{-/-}$ (n=10)
**Basic EKG Parameters**	
BCL (ms)	129±3
PR_{100ms} (ms)	52±7*
QRS_{100ms} (ms)	29±3
**Sinus node function**	
$SNRT_{80ms}$ (ms)	182±37
**AV node characteristics**	
Wenckebach cycle length (ms)	78±6
**Atrial characteristics**	
$AERP_{100ms}$ (ms)	28±6
**Ventricular characteristics**	
$VERP_{100ms}$ (ms)	27±3*
**Atrial Arrhythmias**	
Inducibility (%)	40*
Burden (s)	8±17
Frequency (#)	4±5

Data are mean±SD; asterisk (*) indicates P<0.05 compared to obese WT (Table 2) using two-tailed unpaired Student's t-test. Subscript denotes PCL where appropriate. BCL, basic cycle length; PR_{100ms}, PR interval duration; QRS_{100ms}, QRS duration; SNRT, sinus node recovery time; AVERP, AV nodal effective refractory period; $AERP_{100ms}$, atrial effective refractory period; $VERP_{100ms}$, ventricular effective refractory period.

Supplemental Table 5. Cardiac electrophysiological parameters determined with in vivo EP study in lean SGK1 CA mice

	SGK1 CA (n=5)
**Basic EKG Parameters**	
BCL (ms)	118±8
PR_{100ms} (ms)	52±6*
QRS_{100ms} (ms)	29±2
**Sinus node function**	
$SNRT_{80ms}$ (ms)	162±20
**AV node characteristics**	
Wenckebach cycle length (ms)	78±3
**Atrial characteristics**	
$AERP_{100ms}$ (ms)	28±6
**Ventricular characteristics**	
$VERP_{100ms}$ (ms)	29±5
**Atrial Arrhythmias**	
Inducibility (%)	20
Burden (s)	23±50
Frequency (#)	3±5

Data are mean±SD; asterisk (*) indicates $P<0.05$ compared to lean WT (Table 2) by two-tailed unpaired Student's t-test. Subscript denotes PCL where appropriate. BCL, basic cycle length; PR_{100ms}, PR interval duration; QRS_{100ms}, QRS duration; SNRT, sinus node recovery time; AVERP, AV nodal effective refractory period; $AERP_{100ms}$, atrial effective refractory period; $VERP_{100ms}$, ventricular effective refractory period.

Supplemental Table 6. Optical mapping derived action potential durations, conduction velocity, and AP upstroke velocity in lean WT and SGK1 CA mice

	WT	SGK1 CA
**Right Atrium**	**N=4**	**N=7**
CV (m/s)	0.58±0.08	0.76±0.14
APD50 (ms)	6.9±0.3	8.4±0.89**
APD70 (ms)	11.3±0.5	13.5±1.2**
APD90 (ms)	14.4±0.6	16.4±1.2*
dV/dt (%/ms)	37.8±2.4	32.9±2.6
**Left Atrium**	**N=4**	**N=7**
CV (m/s)	0.78±0.19	0.86±0.35
APD50 (ms)	7.3±0.6	6.9±0.8
APD70 (ms)	12.3±1.0	12.1±1.6
APD90 (ms)	15.3±1.0	15.1±1.7
dV/dt (%/ms)	34.9±4.0	36.7±2.7

Data are mean±SD; double asterisk (**) indicates $P<0.01$; asterisk (*) indicates $P<0.05$ compared to WT; two-tailed unpaired Student's t-test. PCL 100ms for all measurements. CV, conduction velocity; APD50, action potential duration at 50% repolarization; APD90, action potential duration at 90% repolarization; dV/dt, AP upstroke velocity from 20-80% depolarization calculated as percent per ms.

Supplemental Table 7. Cardiac electrophysiological parameters determined with in vivo EP study in WT and SGK1 CA mice fed HFD for 6 weeks

	WT (n=10)	SGK1 CA (n=14)
**Basic EKG Parameters**		
BCL (ms)	123.8±14.2	123.3±14.6
PR_{100ms} (ms)	49.4±4.1	64.3±19.5*
QRS_{100ms} (ms)	27.2±3.5	30.6±4.5
**Sinus node function**		
$SNRT_{80ms}$ (ms)	187.2±43.7	173.6±39.3
**AV node characteristics**		
Wenckebach cycle length (ms)	75.9±4.2	85.1±5.4***
**Atrial characteristics**		
$AERP_{100ms}$ (ms)	21.0±5.7	25.9±7.5
**Ventricular characteristics**		
$VERP_{100ms}$ (ms)	28.0±8.6	32.0±5.4
**Atrial Arrhythmias**		
Inducibility (%)	50	71.4
Burden (s)	11.8±20.5	88.3±156.4
Frequency (#)	3.4±3.7	9.8±6.8*

Data are mean±SD; asterisk (*) indicates P<0.05 compared to WT and triple asterisk (***) indicated P<0.001. Two-tailed unpaired Student's t-test used for all comparisons except inducibility (Fisher exact) and burden/frequency (Mann-Whitney). Subscript denotes PCL where appropriate. BCL, basic cycle length; PR_{100ms}, PR interval duration; QRS_{100ms}, QRS duration; SNRT, sinus node recovery time; AVERP, AV nodal effective refractory period; $AERP_{100ms}$, atrial effective refractory period; $VERP_{100ms}$, ventricular effective refractory period.

Supplemental Table 8. Optical mapping derived action potential durations, conduction velocity, and AP upstroke velocity in WT and SGK1 CA mice fed HFD for 6 weeks

	WT	SGK1 CA
**Right Atrium**	**N=8**	**N=7**
CV (m/s)	0.56±0.12	0.67±0.15
APD50 (ms)	9.3±1.3	11.1±2.8
APD70 (ms)	14.6±1.8	19.4±4.9*
APD90 (ms)	17.2±2.2	24.4±6.9*
dV/dt (%/ms)	34.0±3.3	33.6±5.4
**Left Atrium**	**N=6**	**N=6**
CV (m/s)	0.65±0.14	0.88±0.15*
APD50 (ms)	7.2±1.6	9.1±1.2*
APD70 (ms)	12.4±2.8	16.0±1.9*
APD90 (ms)	15.6±3.3	20.1±2.6*
dV/dt (%/ms)	36.1±3.1	37.7±3.0

Data are mean±SD; asterisk (*) indicates $P<0.05$ compared to WT. PCL 100ms for all measurements by two-tailed unpaired Student's t-test. CV, conduction velocity; APD50, action potential duration at 50% repolarization; APD90, action potential duration at 90% repolarization; dV/dt, AP upstroke velocity from 20-80% depolarization calculated as percent per ms.

Supplemental Table 9: Primer sequences for genes of interest assayed by quantitative PCR

Gene	*Forward Sequence*	*Reverse Sequence*
Col1A1	ATTCCCGTTCGAGTACGGAA	CTCGATCTCGTTGGATCCCT
CTGF	CTGCAGACTGGAGAAGCAGA	GATGCACTTTTTGCCCTTCTT
a-SMA	GTCCCAGACATCAGGGAGTAA	TCGGATACTTCAGCGTCAGGA
TGF-B	GTCACTGGAGTTGTACGGCA	GGGCTGATCCCGTTGATTTC
Cx40	GGTCCACAAGCACTCCACAG	CTGAATGGTATCGCACCGGAA
Cx43	AGTTCCACCACTTTGGCGTG	GTGGAGTAGGCTTGGACCTT
NLRP3	TTTTCACAACTCGCCCAAGG	AAGCTAGAAGTGAGGCAGCA
IL-1B	GGCTCATCTGGGATCCTCTC	TCATCTTTTGGGGTCCGTCA
B-actin	GGCTGTATTCCCCTCCATCG	CCAGTTGGTAACAATGCCATGT
CCL2	CAGCCAGATGCAGTTAACGC	GCCTACTCATTGGGATCATCTTG
CCL5	CAGCAGCAAGTGCTCCAATC	CACACACTTGGCGGTTCCTT
SGK1	GGGTGCCAAGGATGACTTTA	CTCGGTAAACTCGGGATCGA
TIMP1	CTATCCCTTGCAAACTGGAGA	ACCTGATCCGTCCACAAACA
MMP2	CGGAGATCTGCAAACAGGACA	CGCCAAATAAACCGGTCCTT
MMP9	GCGTGTCTGGAGATTCGACTT	TATCCACGCGAATGACGCT
CXCL10	GACGGTCCGCTGCAACTG	CTTCCCTATGGCCCTCATTCT
CCL6	GCTGGCCTCATACAAGAAATGG	GCTTAGGCACCTCTGAACTCTC
VCAM1	CCCAGGTGGAGGTCTACTCA	CAGGATTTTGGGAGCTGGTA

Supplemental Table 10: Antibodies used for immunoblotting.

Antibody	*Species*	*Dilution*	*Product Code*	*Source*
vinculin	Mouse	1:2000	V9264	Sigma-Aldrich
phospho-SGK1 (S422)	Rabbit	1:750	ab55281	Abcam
SGK1	Rabbit	1:1000	ab59337	Abcam
phospho-NDRG1 (T346)	Rabbit	1:1000	5482	CST
NDRG1	Rabbit	1:1000	9408	CST
pGSK3β (S9)	Rabbit	1:1000	9336	CST
GSK3β	Rabbit	1:1000	9315	CST
NaV1.5	Rabbit	1:1000	14421	CST
CTGF	Mouse	1:1000	ab209780	Abcam
Cx40	Rabbit	1:1000	36-4900	Invitrogen
Cx43	Mouse	1:1000	SAB42009780	Sigma-Aldrich
NLRP3	Mouse	1:500	MA5-16274	Thermo-Fisher
Caspase 1	Rabbit	1:1000	2225	CST
p-NFκB	Rabbit	1:750	3033	CST
NFκB	Rabbit	1:1000	8242	CST
Hemagluttinin	Rabbit	1:2000	ab182009	Abcam
GAPDH	Rabbit	1:2000	ab9385	Abcam
